# Fullerene-Perylenediimide (C_60_-PDI) Based Systems: An Overview and Synthesis of a Versatile Platform for Their Anchor Engineering

**DOI:** 10.3390/molecules27196522

**Published:** 2022-10-02

**Authors:** Aurel Diacon, Oksana Krupka, Piétrick Hudhomme

**Affiliations:** 1 Univ. Angers, CNRS, MOLTECH-Anjou, SFR MATRIX, F-49000 Angers, France; 2 Department of Bioresources and Polymer Science, Faculty of Chemical Engineering and Biotechnologies, University Politehnica of Bucharest, 1-7 Gh. Polizu Street, 011061 Bucharest, Romania; 3 Department of Chemistry, Taras Shevchenko National University of Kyiv, 60 Volodymyrska, 01033 Kyiv, Ukraine

**Keywords:** fullerene, perylenediimide, electron/energy transfer, organic photovoltaics, photosynthesis

## Abstract

An overview of the different covalent bonding synthetic strategies of two electron acceptors leading to fullerene-perylenediimide (C_60_-PDI)-based systems, essentially dyads and triads, is presented, as well as their more important applications. To go further in the development of such electron and photoactive assemblies, an original aromatic platform 5-benzyloxy-3-formylbenzoic acid was synthesized to graft both the PDI dye and the fullerene C_60_. This new C_60_-PDI dyad exhibits a free anchoring phenolic function that could be used to attach a third electro- and photoactive unit to study cascade electron and/or energy transfer processes or to obtain unprecedented side-chain polymers in which the C_60_-PDI dyads are attached as pendant moieties onto the main polymer chain. This C_60_-PDI dyad was fully characterized, and cyclic voltammetry showed the concomitant reduction process onto both C_60_ and PDI moieties at identical potential. A quasi-quantitative quenching of fluorescence was demonstrated in this C_60_-PDI dyad, and an intramolecular energy transfer was suggested between these two units. After deprotection of the benzyloxy group, the free hydroxyl functional group of the platform was used as an anchor to reach a new side-chain methyl methacrylate-based polymer in which the PDI-C_60_ dyad units are located as pendants of the main polymer chain. Such polymer which associates two complementary acceptors could find interesting applications in optoelectronics and in particular in organic solar cells.

## 1. Introduction

Photosynthetic and photovoltaic processes harvest energy from sunlight, but they operate in distinctly different ways to produce biomass or chemical fuels in the case of natural photosynthesis and electrical current in the case of photovoltaics [1]. The possibility to generate energy and/or electron transfer and long-lived charge-separated states using fullerenes as electron acceptors has rapidly shown their great advantages to be used in artificial photosynthetic models and organic molecular electronics including solar cells [2]. This story began with the discovery in 1985 by H. Kroto et al. of fullerene C_60_ [3], and this allotropic form of carbon then sparked the imagination of organic chemists who used its nano-sized football geometry as a new Lego^®^ block. The large-scale production in the early 1990s was the first breakthrough [4,5] that allowed the development of chemical methodologies to functionalize C_60_. This has made C_60_ a fascinating molecule for building molecular assemblies with remarkable physical and chemical properties, leading to a wide range of interesting applications, from materials to medicinal sciences [6,7]. In particular, its electrochemical properties with six reversible single-electron reduction waves [8], combined with interesting photophysical properties, make C_60_ a unique electron acceptor to study photoinduced electron transfer processes.

In particular, the ability of a pi-conjugated polymer to efficiently transfer electrons to C_60_, giving rise to long-lived charge-separated states [9], has suggested that fullerene derivatives could be used for photovoltaic applications [10]. This was the starting point for the explosion of interest in their use as materials in the active layer of organic solar cells (OSCs). The synthesis by F. Wudl and coll. in 1995 of a functionalized C_60_ derivative, named [6,6]-phenyl-C_61_-butyric acid methyl ester PC_61_BM) [11], paved the way for the great development of organic photovoltaics (OPVs) using the polymer/fullerene bulk-heterojunction (BHJ) concept [12]. The construction of BHJ devices consists in mixing together donor and acceptor materials to form a bicontinuous interpenetrating network and they have attracted much attention as they offer the possibility to achieve high power conversion efficiencies (PCE). Thanks to their geometrical tridimensional architecture, good electron mobility, excellent electron properties, and good miscibility with conductive polymers, fullerene derivatives were considered for two decades as the spearhead of the acceptors for the development of OPVs [13]. However, despite these promising characteristics, fullerene-based OSCs exhibited serious drawbacks, including poor light-absorption properties, limited energy-level tunability, and significant morphological and/or photochemical instability [14]. To overcome these limitations, tremendous efforts have been directed toward the synthesis of non-fullerene acceptors (NFAs), especially small molecular acceptors (SMAs), and their development in related OSCs [15,16,17,18,19]. Their main advantage is their easily tunable energy levels and better absorption properties giving significantly higher open-circuit voltages (V_OC_) and photocurrents (J_SC_), respectively, than conventional fullerene-based devices. Last, SMAs with proper molecular design can also be more chemically stable, thus extending the device lifetime of OSCs [20].

On the front of applications involving fullerenes under the influence of light irradiation, energy and electron transfer processes are essential photochemical events in photosynthesis [21]. The conversion of solar energy into chemical energy is initiated by the light-harvesting of various chromophores, so-called antenna complexes, and the funneling of the produced exciton by energy transfer to a specific site, known as the reaction center, in which a subsequent electron transfer to an electron acceptor can occur [22,23]. The mimicry of natural photosynthesis by artificial systems holds the promise for cheap, environmentally friendly energy generation. Consequently, the incorporation of artificial light-harvesting antennas into molecular electron donor–acceptor systems was regarded as one of the most interesting strategies for the design of optoelectronic materials in photon-energy conversion. In this area, the covalent bonding of a light-harvester, capable to act as an electron donor, to C_60_ has emerged as an active field of research to reach dyads in which intramolecular electronic interactions dominate generating a long-lived charge-separated state [24].

Among these materials capable to act as light-harvesting absorbers, the iconic perylenediimide (PDI) presents many advantages since this photoactive molecule exhibits high chemical, thermal and photochemical stability, wide and intense optical absorption in the visible to near-infrared spectral window, high photoluminescence quantum yield, and excellent charge transport properties [25,26]. These PDI derivatives are of great interest to be studied as alternative electron acceptors to fullerenes in OPVs due to their tunable absorption in the visible range, inexpensive synthesis, and photochemical stability [27,28,29,30]. Moreover, the idea of covalently linking the fullerene with PDI quickly became obvious in order to create dyad systems that were rapidly considered as light-harvesting fullerenes in the search for photoinduced electron and/or energy transfer processes. These systems present high potential to be used in OSCs [31] or as organic triplet photosensitizers with potential applications in photocatalysis, photooxidation, and photodynamic therapy or for artificially mimicking the photosynthesis phenomenon [32].

This presentation aims to highlight the different synthetic strategies elaborated to build C_60_-PDI-based systems and their potential for applications in materials chemistry (Section 2). Moreover, for the development of such electron and photoactive assemblies, an original aromatic platform of 5-benzyloxy-3-formylbenzoic acid has been synthesized to graft both the PDI dye and the fullerene C_60_ (Section 3). This new C_60_-PDI dyad exhibits a free anchoring phenolic function that could be used to attach a third electro- and photoactive unit to study cascade electron and/or energy transfer processes or to obtain unprecedented side-chain polymers in which the C_60_-PDI dyads are attached as pendant moieties onto the main polymer chain.

## 2. State-of-the-Art of Covalently Linked C_60_-PDI Based Systems

**Figure 1:**
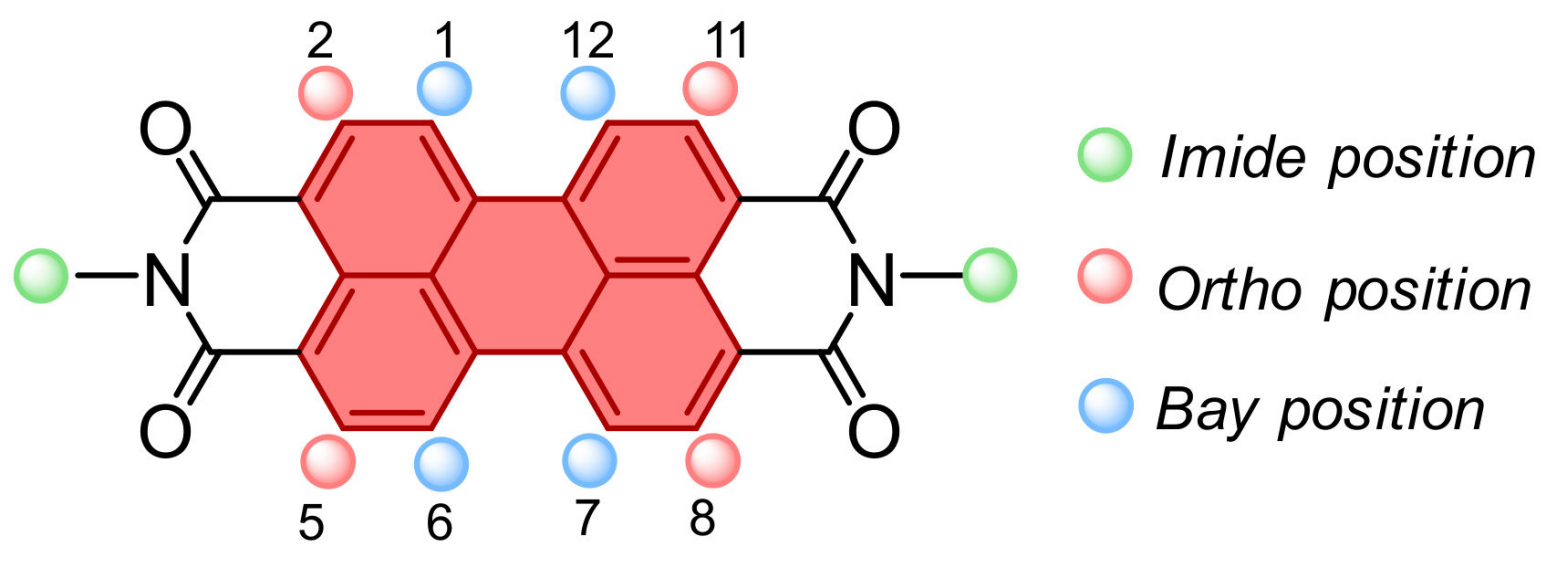
Strategies for functionalization of perylenediimide (PDI) scaffold.

This overview focuses on the different synthetic methodologies that have been used so far to graft the PDI unit onto C_60_. The perylene scaffold has three different types of functionalizable positions. The 3,4,9, and 10 positions known as the *peri* positions correspond to the diimide functions for PDI derivatives (Figure 1) [33]. The introduction of substituents at this imide nitrogen atoms significantly improves the solubility of the corresponding materials. The other positions 1,6,7, and 12, called *bay* positions, and 2,5,8, and 11 *ortho* positions play a key role in tuning their optical and electronic properties. Considering the recent development of functionalization on *ortho* positions, to our knowledge no C_60_-PDI dyad has been reported to date considering this possibility. Therefore, we will only consider the development of strategies to attach C_60_ to the imide position or the bay region of the PDI core.

Among the different strategies available for the functionalization of C_60_ fullerene, it should be noted beforehand that most of the examples described here involve the [2 + 1] and [3 + 2] cycloadditions, more commonly known as the Bingel [34] and Prato [35] reactions, respectively.

To carry out the Prato reaction, an amino acid derivative, most often N-methylglycine (sarcosine), reacts with an aldehyde in refluxing toluene (chlorobenzene or *o*-dichlorobenzene can also be used) to generate an azomethine ylide which interacts with a 6:6-double bond in a 1,3-dipolar cycloaddition to yield the N-methylpyrrolidine derivative.

The Bingel reaction can be carried out on α-halomalonate in the presence of a base to firstly generate the corresponding anion which adds to C_60_ via an addition–elimination process by intramolecular displacement of the halide to give the corresponding methanofullerene. This nucleophilic cyclopropanation is improved by directly using the malonate from which the α-halomalonate is produced in situ in the presence of iodine or CBr_4_ and 1,8-diazabicyclo [5.4.0]undec-7-ene (DBU) acting as a base. The reaction using iodine was initially developed by J.-F. Nierengarten and F. Diederich [36], whereas the reaction with CBr_4_ is known as the Bingel–Hirsch reaction [37].

### 2.1. Grafting C_60_ at the Imide PDI Scaffold

**Scheme 1:**
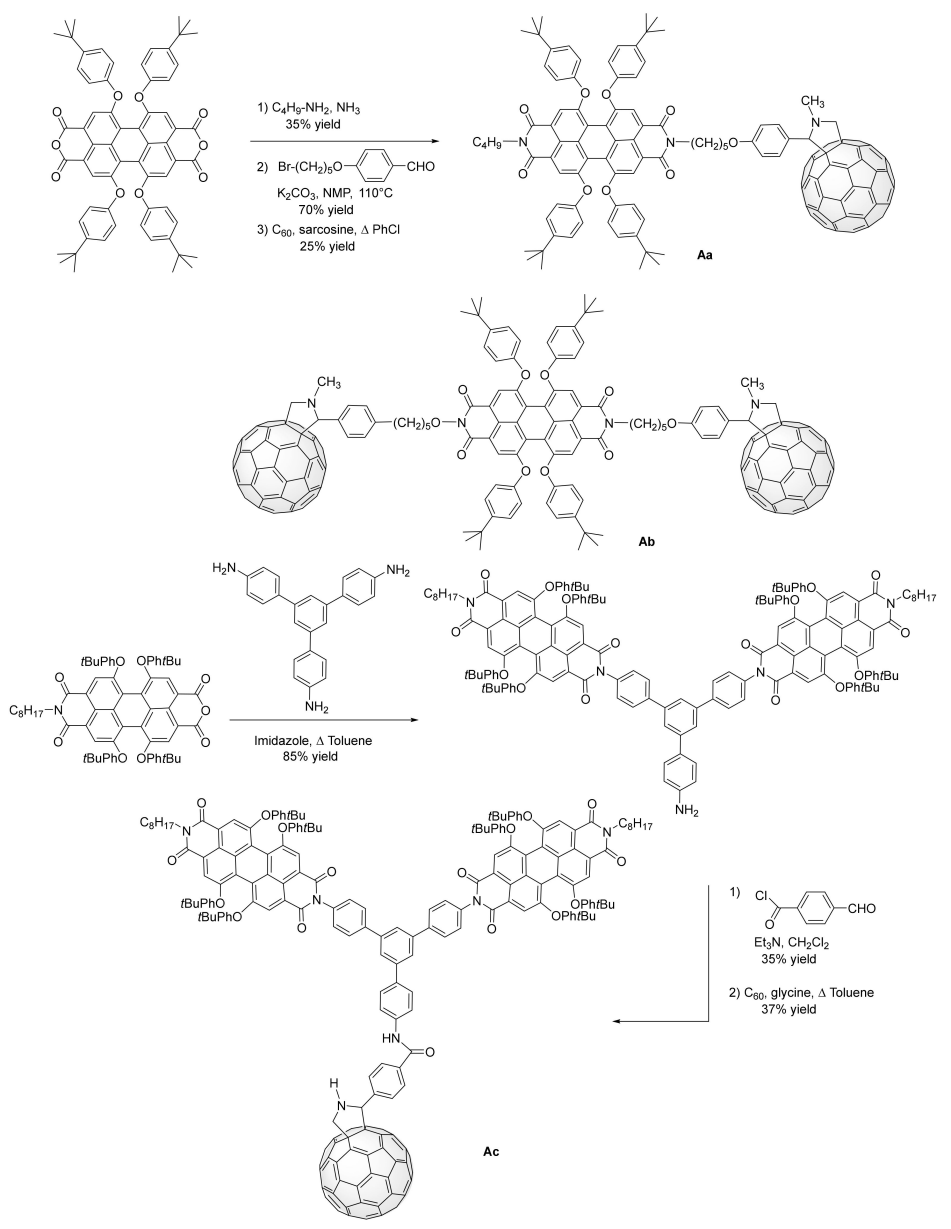
Synthesis of PDI-C_60_ dyad **Aa** and C_60_-PDI-C_60_ dumbbell **Ab** by H. Tian and coll., triad PDI-C_60_-PDI **Ac** by D. Zhu and coll.

Over the past two decades, electro- and photoactive C_60_-PDI dyads have been synthesized by attaching C_60_ to the imide position. Unlike triad access, which can utilize both symmetrically substituted positions of the PDI motif, the key step for dyad syntheses requires desymmetrization of the PDI scaffold. Starting with 1,6,7,12-tetrakis(4-*tert*-butylphenoxy)perylene dianhydride, the reaction of ammonia and *n*-butylamine afforded the asymmetric PDI derivative. After alkylation to introduce the aldehyde linker, the Prato reaction with C_60_ and sarcosine was used by H. Tian and coll. to obtain dyad **Aa** (Scheme 1) [38]. Electrochemical and photophysical studies in solution revealed that there is no significant ground-state electronic interaction between both PDI and C_60_ partners. These studies have shown that singlet-singlet energy transfer from the PDI unit to C_60_ occurs predominantly in dyad **Aa**. A photovoltaic device using this dyad confirmed that the efficiency of the photoinduced electron transfer is negligible compared to energy transfer. Using this methodology, symmetrical C_60_-PDI-C_60_ dumbbell **Ab** was prepared and found to exhibit good solubility, high thermal stability, and broad visible-light absorption [39]. Photophysical studies confirmed the presence of a photoinduced energy transfer from the PDI moiety to C_60_.

Using this highly soluble perylene dianhydride, D. Zhu and coll. developed a synthetic route to reach PDI-C_60_-PDI triad **Ac** [40]. Firstly, the unsymmetrically substituted PDI is classically synthesized from the corresponding *N*,*N*′-dialkyl-1,6,7,12-tetrakis(4-*tert*-butylphenoxy)PDI which is hydrolyzed in basic medium leading to a mixture of the perylene monoanhydride and the perylene bisanhydride that are separated by column chromatography [41]. The imidization of the perylene monoanhydride using 1,3,5-tris(4-aminophenyl)benzene [42] was followed by the reaction of the remaining amino functionality with 4-formylbenzoyl chloride. Finally, 1,3-dipolar cycloaddition to C_60_ in the presence of glycine afforded triad **Ac** for which the strong quenching of the PDI fluorescence was suggested to result from an intramolecular photoinduced charge-transfer process within the triad.

**Scheme 2:**
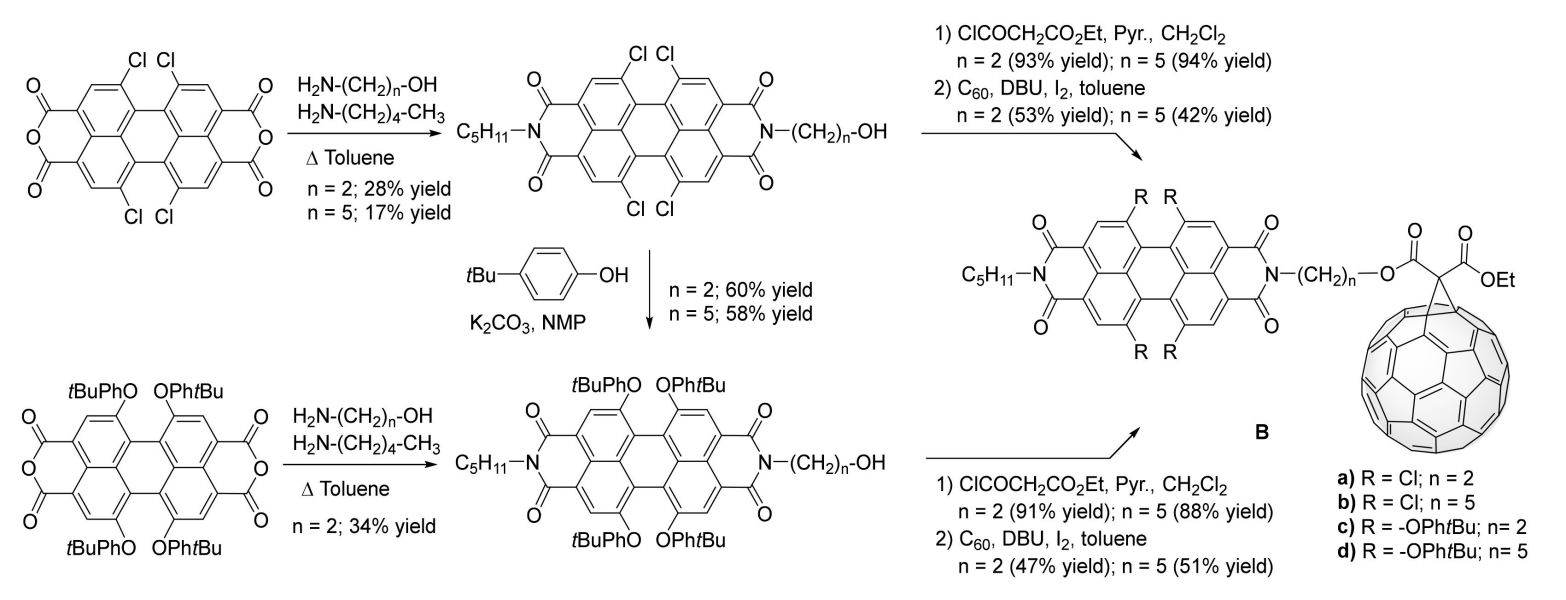
Synthesis of PDI-C_60_ dyads **Ba–d** developed by P. Hudhomme and coll.

Our group, in collaboration with R. M. Williams, was interested in the concept of super-absorbing fullerenes obtained by linking a dye molecule to fullerene C_60_ as new systems exhibiting efficient light-harvesting properties. The objective was to consider that the dye could act as an antenna by absorption of sunlight inducing an intramolecular energy transfer towards the fullerene, playing the role of energy receptor. The corresponding solar cells should be designed considering that inside the photoactive layer the following combination of events could occur: (i) self-assembly into an interpenetrating nanoscopic network, (ii) an energy transfer from PDI towards fullerene C_60_ with the dye acting as a light-harvesting antenna, and (iii) a selective electron transfer between the p-type polymer donor such as poly(3-hexylthiophene) (P3HT) to the C_60_ unit. One of the objectives was to demonstrate that the nature of the substituents on the PDI bay region strongly influences the electronic properties of these dyads for their subsequent use in photovoltaic devices. Furthermore, an interesting feature in the design of these C_60_-PDI dyads was to demonstrate that the distance between PDI and C_60_ moieties and their mutual orientation could be crucial parameters for the energy transfer rate and could play an important role in the electronic interaction between the two partners. The key step in the preparation of dissymmetrical PDI was achieved by direct condensation of 1,6,7,12-tetrachloroperylene dianhydride with two aliphatic amine derivatives of similar reactivity such as pentan-1-amine and 2-aminoethanol or 5-aminopentan-1-ol in stoichiometric ratio and in refluxing toluene [43,44]. After separation from the two symmetric compounds, the subsequent introduction of *tert*-butylphenoxy groups could be carried out on the asymmetric alcohol using an aromatic nucleophilic substitution of chlorine atoms with 4-*tert*-butylphenol in the presence of K_2_CO_3_. Transformation of the alcohol to the malonate functionality was performed using ethyl malonyl chloride in the presence of pyridine. The cyclopropanation was carried out by reaction with C_60_ in the presence of iodine and DBU to give corresponding C_60_-PDI dyads **Ba–d** in satisfactory yields (Scheme 2) [45,46].

Electronic properties being tuned by an appropriate substitution of the PDI bay region, the first reduction unambiguously occurs on the PDI moiety in the case of dyads **Ba** and **Bb**, but the reduction potentials of the PDI moiety are shifted to more negative values when chlorine atoms were substituted by *tert*-butylphenoxy groups. Consequently, the C_60_ moiety appears to be the favored electron acceptor inside dyads **Bc** and **Bd**. The electron-withdrawing inductive effect of the chlorine atoms stabilizes the anion radical PDI^−^˙, whereas the electron-donating mesomeric effect of phenoxy groups appears predominant [47]. The photophysics of C_60_-PDI systems is also governed by their bay substituents and the distance between the chromophores that mainly determine the presence of energy or electron transfer. It was shown in solvents of different polarity that C_60_-PDI systems containing chlorine atoms (**Ba,b**) or *tert*-butylphenoxy groups (**Bc,d**) as bay substituents display an efficient singlet-singlet energy transfer from the PDI unit to C_60_, thus acting as light-harvesting antenna to the C_60_ playing the role of energy acceptor [46,48,49]. The excited fullerene unit thus created (^1^C_60_) displays its characteristic spin-orbit coupling and converts to the triplet state with efficiency close to unity. This ^3^C_60_ can then act as an intramolecular triplet sensitizer populating the triplet state of the PDI unit. Such PDI compounds that have very high triplet quantum yields and present intramolecular triplet sensitization could yield systems that are very suited for singlet oxygen production for photodynamic therapy applications, especially since the compounds absorb strongly in the 500–700 nm region. The potential use of these light-harvesting fullerenes in OSCs was estimated with their incorporation in BHJ using P3HT as the pi-conjugated donor polymer. The role of a light-harvesting antenna grafted onto C_60_ was demonstrated thanks to the presence of an efficient energy transfer from the PDI towards fullerene C_60_. Moreover, the photovoltaic behavior was in agreement with electrochemical data which showed that PDI should be favored to play the role of acceptor instead of C_60_ in dyad **Bc** with a higher efficiency compared to the device using dyad **Ba** [50].

**Scheme 3:**
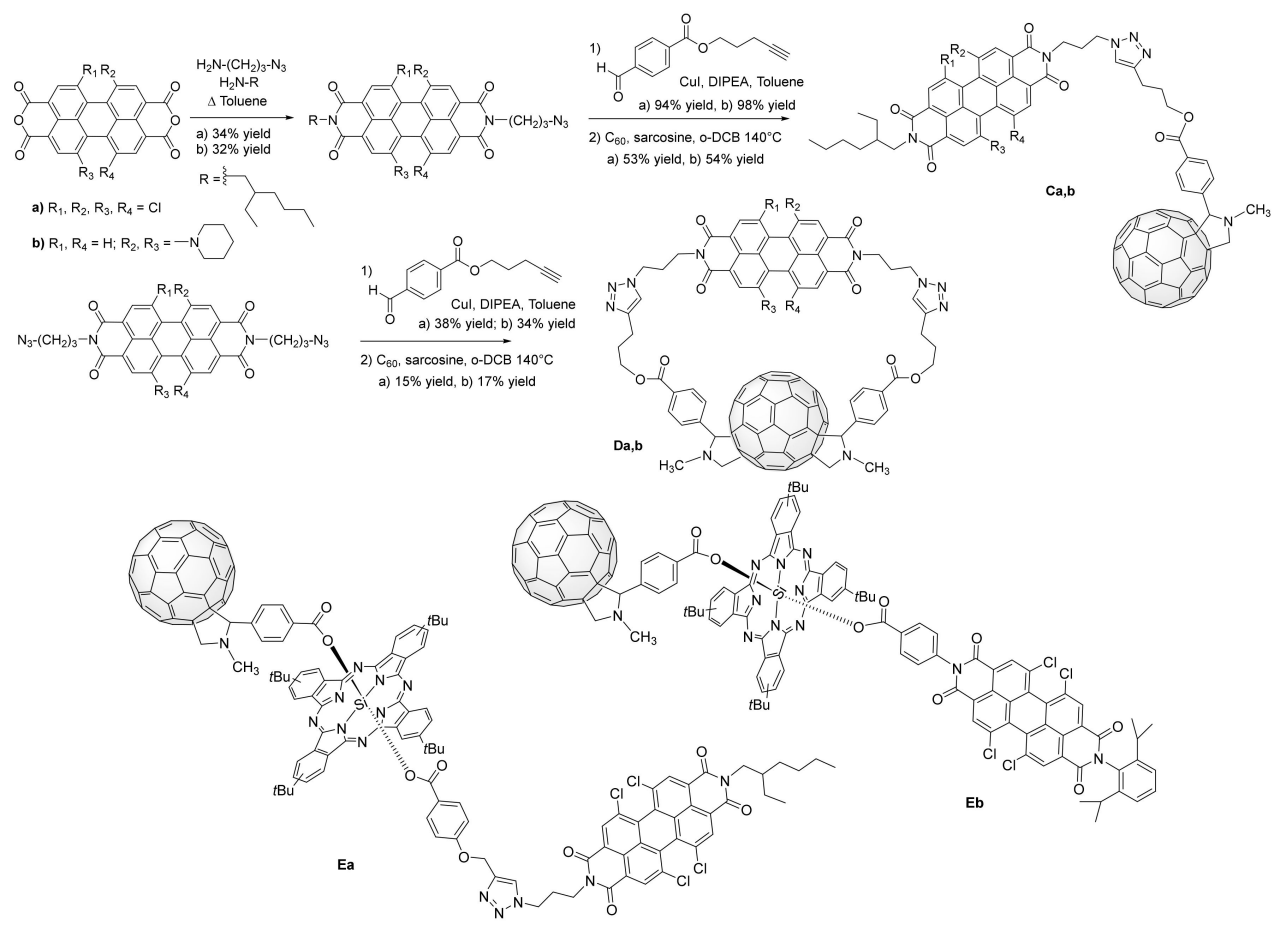
Synthesis of linear PDI-C_60_ dyads **C**, macrocyclic PDI-C_60_ dyad **D** and extension to multichromophoric PDI-SiPc-C_60_ triads **Ea** and **Eb** by A. Sastre Santos and coll.

This strategy for bay-PDI desymmetrization, using the statistical imidization reaction with two different amines on the perylene dianhydride [43,44,51], was applied by A. Sastre Santos and coll. to prepare dyad **C** [52]. Introduction of the formyl derivative using a copper-catalyzed azide-alkyne cycloaddition was followed by the Prato 1,3-dipolar cycloaddition to afford linear dyads **Ca** and **Cb** (Scheme 3). Starting from the double condensation of 3-azido-1-propylamine, the PDI diazide derivative was used as a precursor of macrocyclic dyad **Da** and **Db** obtained as a mixture of regioisomeric C_60_ cycloadducts. It was noted that compound **Da** was accompanied with the symmetrical linear C_60_-PDI-C_60_ triad. Photophysical studies showed a photoinduced energy transfer from ^1^PDI* to C_60_ which was followed by intersystem crossing from ^1^C_60_* to ^3^C_60_* in the case of PDICl_4_-C_60_ dyads that is, in particular, one order of magnitude faster in the cyclic dyad **D** compared to the linear one **C**. Using these intermediates and synthetic strategies, the electron donor silicon phthalocyanine (SiPc) was incorporated between the two electron acceptors of the multichromophoric PDI-SiPc-C_60_ triad **Ea** [53]. An ultrafast singlet-singlet energy transfer from ^1^PDI* to SiPc ultimately led to the two PDI-SiPc^+^-C_60_^−^ and PDI.^−^SiPc^+^-C_60_ charge-separated states. Compared to the triad **Eb**, it was shown that the spacer length plays an important role in governing the excited state energy and the electron transfer events [54].

**Scheme 4:**
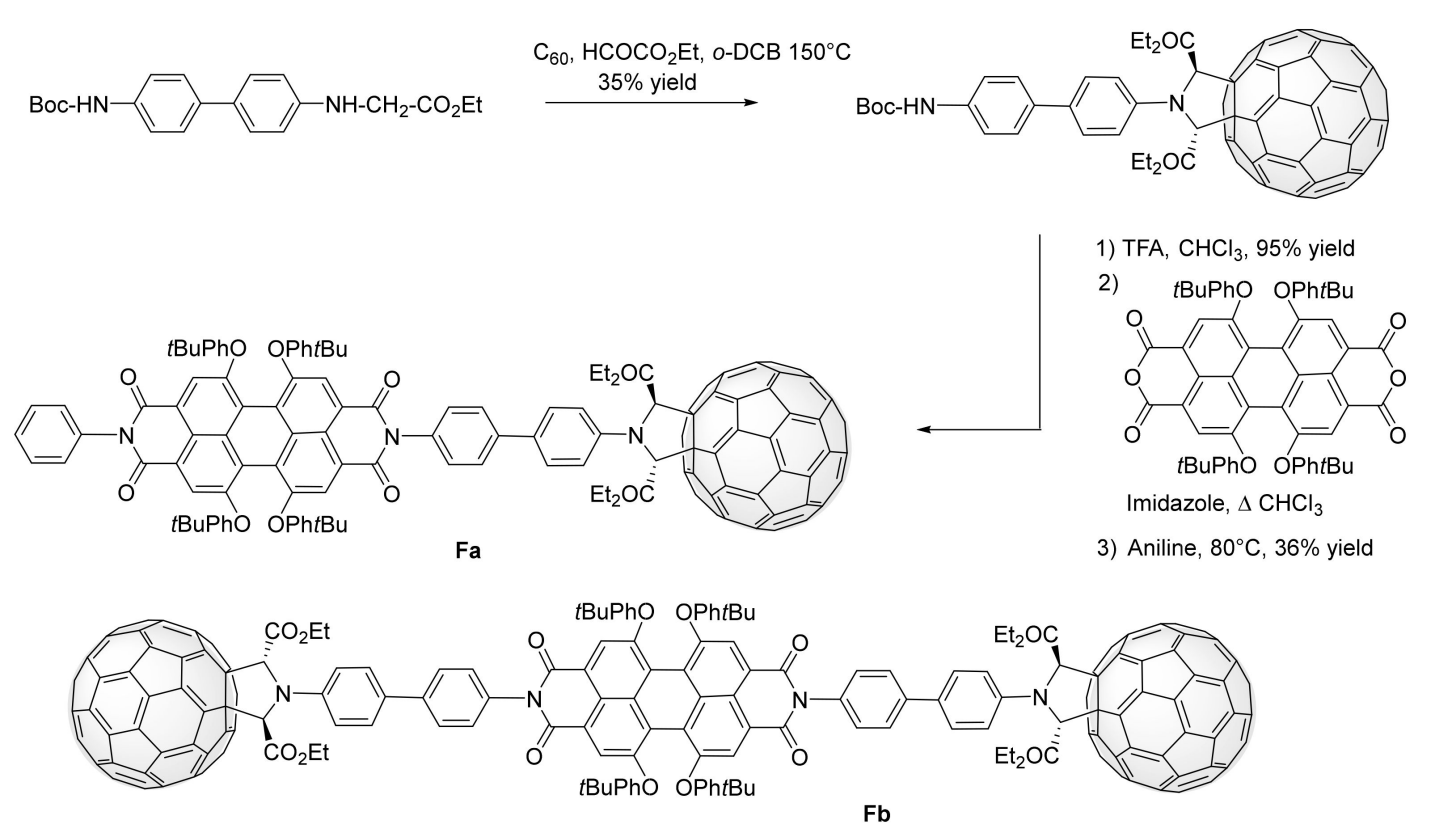
Introduction of a rigid spacer in C_60_-PDI dyad **Fa** and PDI-C_60_-PDI triad **Fb** by G.-W. Wang and coll.

To prevent through-space interaction and with the aim of possibly enhancing the energy transfer process, both C_60_ and PDI partners were linked by a rigid biphenyl spacer allowing a fixed spatial distance between them. The synthesis of dyad **Fa** carried out by X.-F. Wang, G.-W Wang and coll. involves a 1,3-dipolar cycloaddition of the azomethine ylide generated in situ from the condensation of the glycinate group with C_60_ (Scheme 4) [55]. Triad **Fb** was prepared by imidization of the perylene dianhydride in 44% yield. In fact, an efficient intramolecular energy transfer from PDI antenna to C_60_ is followed by an intrinsic intersystem crossing of C_60_ leading to the production of C_60_ triplet. This property of a light-harvesting antenna combined with an intersystem crossing was used as an efficient photooxidation system for the transformation of 1,5-dihydroxynaphthalene into juglone, showing that the C_60_-PDI dyad could be used as a triplet photosensitizer. The presence of two C_60_ units showed a 1.3-fold increase in the efficiency of **Fb** compared to that of **Fa**.

**Scheme 5:**
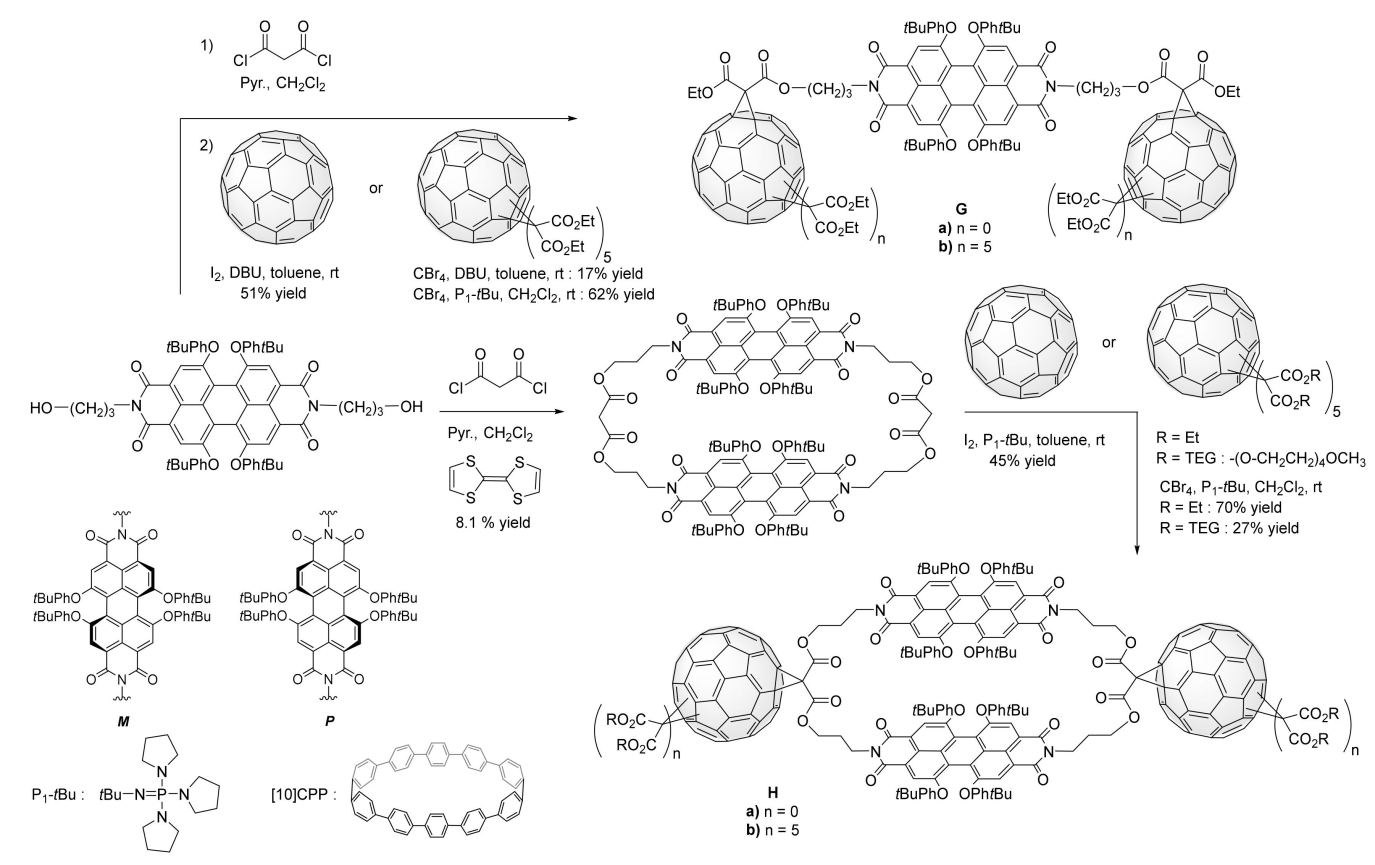
Dumbbell-like molecules **G** and PDI-cyclophanes functionalized with C_60_ **H** by A. Hirsch and coll.

Very recently, A. Hirsch and coll. described the synthesis of dumbbell-like molecules and PDI-cyclophanes functionalized with fullerenes (Scheme 5) [56,57]. Starting from the PDI dialcohol, the bis-malonate was prepared using dimalonyl chloride in the presence of pyridine as a base. Whereas the experimental conditions were using the couple iodine and DBU for cyclopropanation of C_60_ with the corresponding malonate, the typical Bingel-Hirsch conditions (CBr_4_/DBU) were used when using C_60_ pentakisadducts [58]. Nevertheless, in the latter case, to overcome the low yield to afford dumbbell **G**, DBU was efficiently replaced by the Schwesinger phosphazene base (P_1_-*t*Bu) for the in situ generation of the α-bromomalonate intermediate [59]. On the other hand, the preparation of the cyclophane was carried out from the PDI dialcohol with malonyl dichloride. The Ziegler–Ruggli principle, taking place under high dilution conditions, was applied to avoid polymerization reactions and to promote intramolecular ring closure. Additionally, the electron-donor tetrathiafulvalene (TTF) was added to the mixture to favor a template-controlled synthesis thanks to the promotion of a pre-arranged sandwich-like structure. To carry out in a satisfactory way the cyclopropanation affording assemblies **H**, the P_1_-*t*Bu base was associated either with iodine in toluene for **Ha** using pristine C_60_ or CBr_4_ in CH_2_Cl_2_ in the case of C_60_ pentakisadducts **Hb** bearing ethyl or tetraethylene glycol (TEG) groups.

Bay-substituted PDI are twisted due to the steric hindrance of their substituents with the possible existence of two (*M*/*P*) atropoisomers (Scheme 5) [60]. In the case of 4-*tert*-butylphenoxy groups in the 1,6,7,and 12 positions, the *M* *↔ P* interconversion is known to be fast at room temperature [61]. Whereas fast atropoisomerization was observed in ^1^H-NMR spectra for the linear PDI-bismalonate, this was shown to be slow at the NMR timescale for the PDI-cyclophane. The resulting diastereoisomers *MM*/*PP* and *MP*/*PM* were found in a 10:1 ratio, respectively, in C_2_D_2_Cl_4_ at −15 °C as a result of attractive π−π interactions between the two PDI moieties [56]. It was noted that the co-facial π−π stacking arising in the PDI-cyclophane-(C_60_)_2_ **Hb** bearing ethyl groups leads to a complete diastereoselectivity. Moreover, the physico-chemical properties are also governed by these π−π-interactions between PDI units in the cyclophane and the bulky tetrakis(4-*tert*-butylphenoxy) substituents suppress the tendency towards aggregation [62]. On the other hand, starting from dumbbell-like molecule **Ga** and PDI-cyclophane-(C_60_)_2_ **Ha**, pseudorotaxanes with [10]cycloparaphenylene ([10]CPP) were investigated using NMR and fluorescence spectroscopies, but also the calorimetric (ITC) method, to determine the binding stoichiometries as well as the thermodynamic and kinetic parameters. It was observed that the formation of 1:1 or 1:2 complexes in *o*-DCB depends on the stoichiometry of added [10]CPP, thus demonstrating that the rigidity of **Ha** has no influence on the supramolecular interaction with [10]CPP [57].

**Scheme 6:**
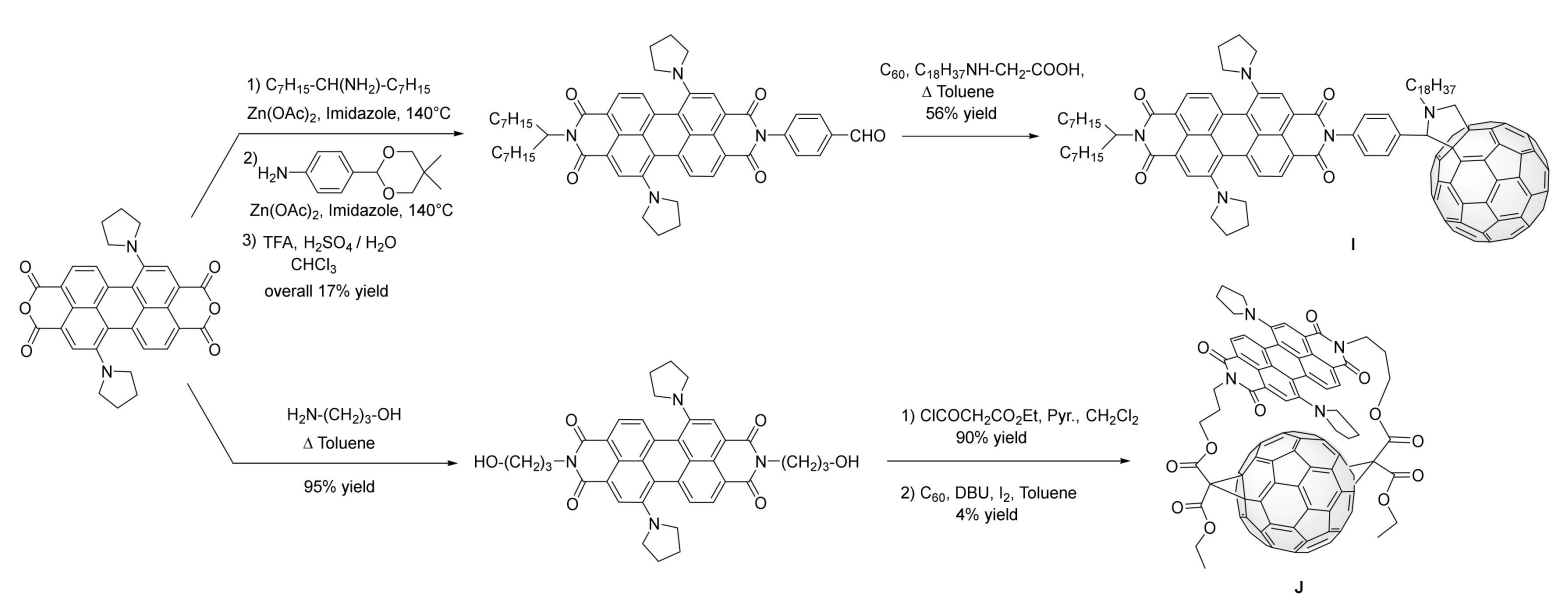
Linear and double-bridged 1,7-pyrrolidinylPDI-C_60_ dyads **I** and **J**, respectively, prepared by N. V. Tkachenko with H. Imahori, A. Sastre Santos and coll.

The introduction of pyrrolidinyl groups on the bay positions allows the PDI core to act as a donor and consequently undergo an electron transfer towards C_60_. In this way, electron-rich PDI-C_60_ dyad **I** was synthesized by N. V. Tkachenko, H. Imahori and coll. using the desymmetrization strategy applied on 1,7-dipyrrolidinyl perylene dianhydride. After a three-step synthesis leading to a PDI aldehyde, the following 1,3-dipolar cycloaddition afforded PDI-C_60_ dyad **I** (Scheme 6) [63]. Photo-induced electron transfer was shown to occur from the PDI excited singlet state to C_60,_ generating the corresponding charge-separated state in polar solvents (benzonitrile, pyridine, and *o*-DCB). In contrast, singlet-singlet energy transfer takes place from the PDI to C_60_ in nonpolar solvent (toluene), followed by an intersystem crossing to the C_60_ excited triplet state and a subsequent triplet-triplet energy transfer to yield the PDI excited triplet state [64]. N. V. Tkachenko, A. Sastre Santos and coll. prepared 1,7-dipyrrolidinyl PDI-C_60_ double-bridged dyad **J** using a modified Bingel cyclopropanation [65]. This dyad undergoes an electron transfer in polar (benzonitrile) and nonpolar (toluene) solvents showing that these dyads behave as artificial photosynthetic models in terms of charge separation.

**Scheme 7:**
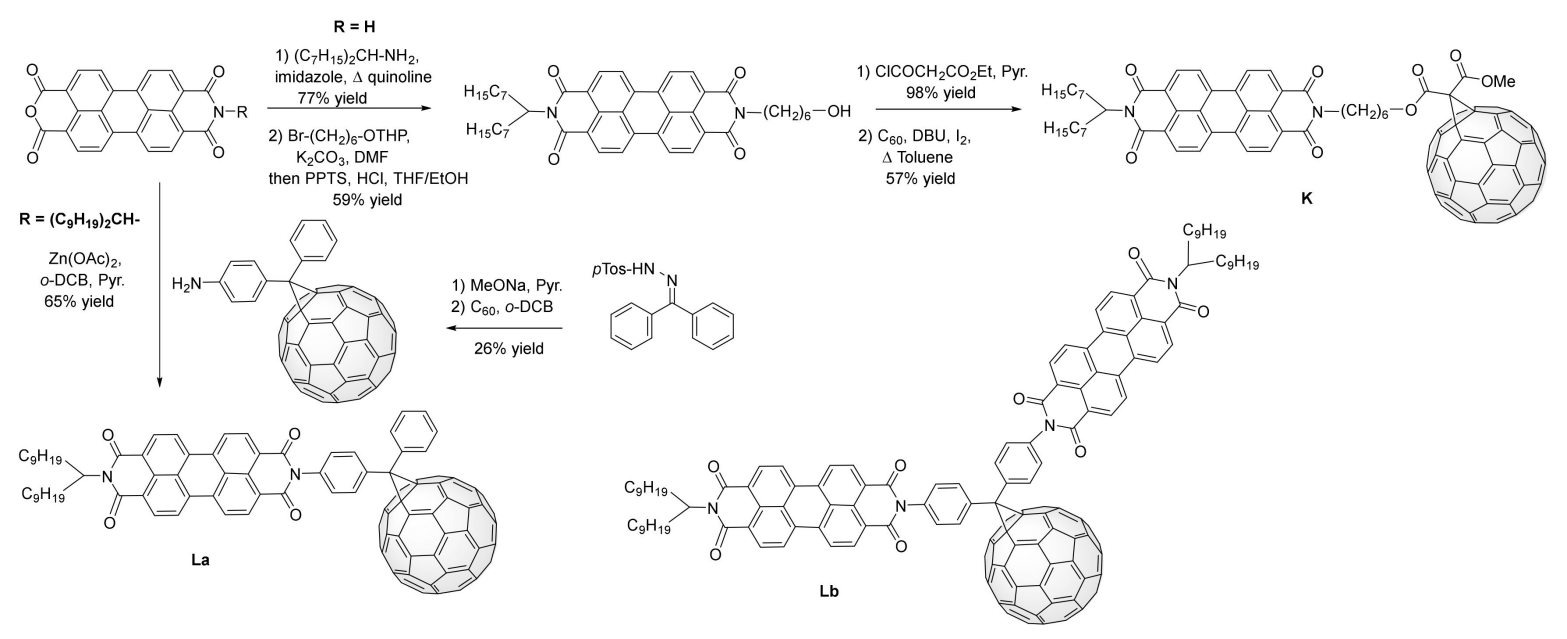
Synthetic strategies for unsubstituted PDI-C_60_ dyad **K** by J. Köhler and coll, dyad **La** and triad **Lb** by J.L. Segura, N. Martín and coll.

For PDI unsubstituted on the bay region, the alternative for desymmetrization consists in the preparation from perylene dianhydride (PTCDA) of the PDI monoanhydride derivative [66]. This strategy was used by J. Köhler and coll. to prepare C_60_-PDI dyad **K** for which a near 100% energy transfer occurs from PDI to C_60_. The fullerene in turn undergoes an intersystem crossing followed by a triplet energy back transfer to the antenna with an efficiency of at least 20% [67]. The initial synthesis of the key dissymmetrical PDI intermediate was obtained by imidization on the anhydride function, followed by alkylation with protected 6-bromohexanol. The deprotected alcohol function was esterified and the resulting malonate was submitted to a final modified Bingel cycloaddition to give dyad **K** (Scheme 7).

On the contrary, J. L. Segura, N. Martín and coll. have used the Bamford–Stevens reaction to prepare diphenylmethanofullerene-PDI dyad **La** and corresponding triad **Lb** [68]. This cycloaddition reaction is known to transform tosylhydrazones into alkenes under strong basic conditions [69]. It was firstly applied in fullerene chemistry for the synthesis of famous PC_61_BM [11] and [PC_71_BM [70] fullerene derivatives. The critical step corresponds to the transformation of a *p*-toluenesulfonylhydrazide into a diazoalcane intermediate in basic medium using sodium methoxide in pyridine. In a one-pot procedure, trapping with C_60_ in refluxing *o*-DCB via a 1,3-dipolar cycloaddition afforded the open 5:6-fulleroid, as the kinetic product, which was not isolated. The latter was converted in situ into the 6:6-closed methanofullerene as the more stable compound. The perfect match between the excitation and the absorption spectrum strongly suggests a quantitative energy transfer for dyad **La** and triad **Lb** for which a quenching of fluorescence, 99% and 95%, respectively, was observed.

### 2.2. Grafting C_60_ onto Bay-PDI Backbone

**Scheme 8:**
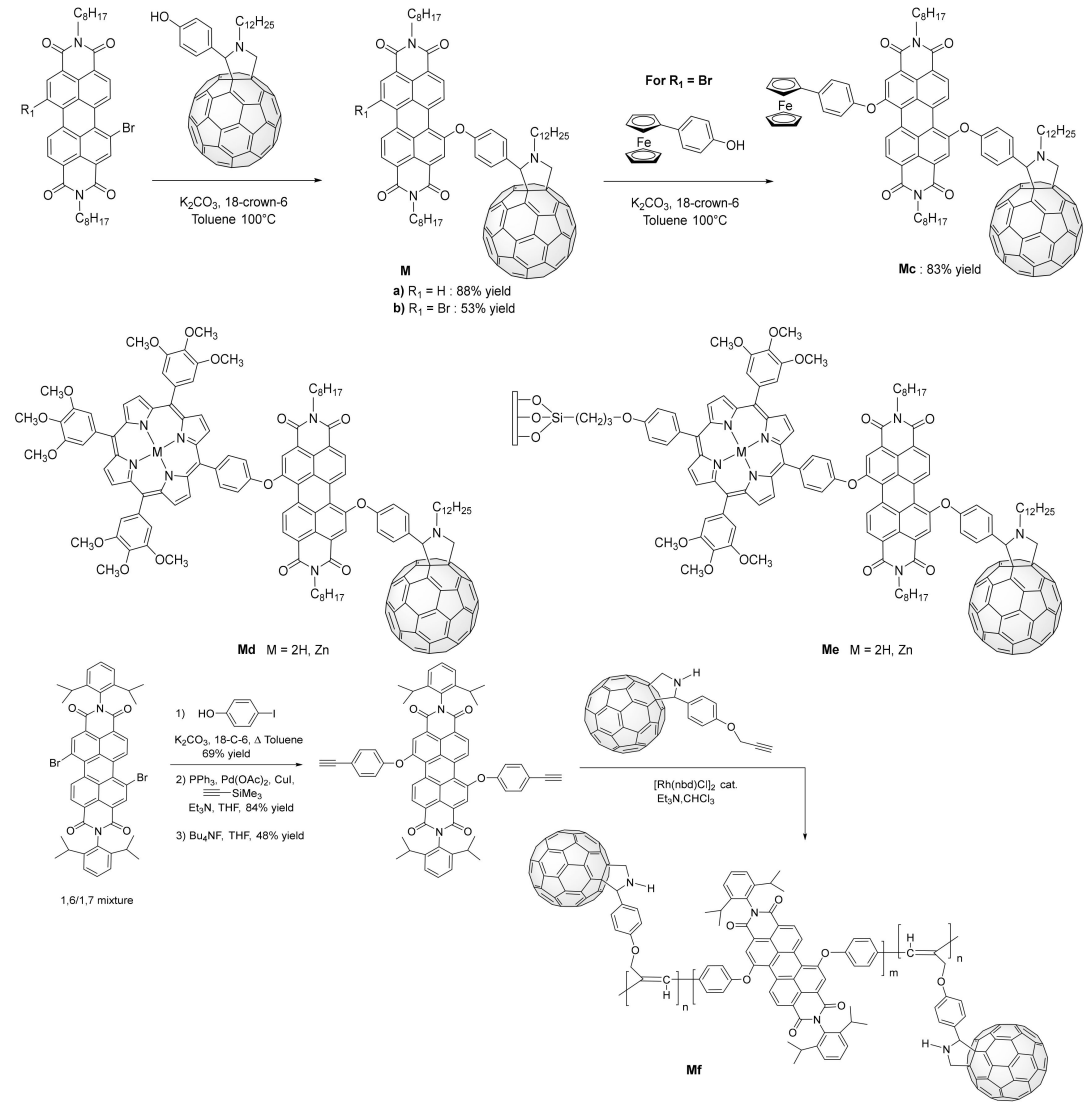
Bay-PDI grafting of C_60_ derivative using aromatic nucleophilic substitution for dyads **Ma,b,c** and extension to the preparation of triads **Md,e** and conjugated polyacetylene **Mf** bearing PDI units and pendant C_60_ acceptors by D. Zhu and coll.

The introduction of C_60_ on the bay PDI region was initiated by D. Zhu and coll. with preparation of dyads **Ma** and **Mb** by nucleophilic substitution using a fulleropyrrolidine bearing a phenol group onto 1-bromoPDI or 1,7-dibromoPDI in the presence of K_2_CO_3_ and 18-crown-6 (Scheme 8) [71,72]. After this first moderately selective substitution, the free bromine atom from **Mb** was substituted to introduce a ferrocene or zinc porphyrin unit giving corresponding triads **Mc** and **Md** with which photovoltaic devices were fabricated. It should be noted that in the Fc-PDI-C_60_ triad, a N-type cascade electron transfer from the ferrocene (Fc) to the PDI and then from the PDI unit to C_60_ was proposed [73]. As an extension of this work, self-assembled monolayers (SAMs) of porphyrin-PDI-C_60_ triad **Me** were constructed on the indium tin oxide (ITO) surface as photoelectrochemical systems. The incorporation of the PDI as an accessory pigment was intended to complement the absorption of the porphyrin taking advantage that light absorption of PDI and porphyrin occurs in complementary regions, giving extended absorption in the visible spectral window [74].

The dibromination of PTCDA classically yields a mixture of 1,6/1,7-dibromo analog in a 1/3 ratio [75]. From this starting material, conjugated polyacetylenes **Mf** bearing PDI units and pendant C_60_ acceptors were synthesized by D. Zhu and coll [76]. The nucleophilic substitution of the two bromine atoms using 4-iodophenol was subsequently followed by pallado-catalyzed coupling with trimethylsilylethyne in trimethylamine, then desilylation of protecting groups. The acetylene-containing monomers were copolymerized in the presence of 2-(4-ethynyloxyphenyl)-3,4-fulleropyrrolidine [77] with transition metal catalyst [Rh(nbd)Cl]_2_, this rhodium catalyst being reported to be effective for the polymerization of monosubstituted acetylenes affording high molecular weight stereoregular polymers with the cis C=C backbone. Interestingly, the photocurrent measurement showed that the films of this polyacetylene could produce steady and rapid cathodic photocurrent responses, indicating that efficient charge transfer took place in this polymer.

**Scheme 9:**
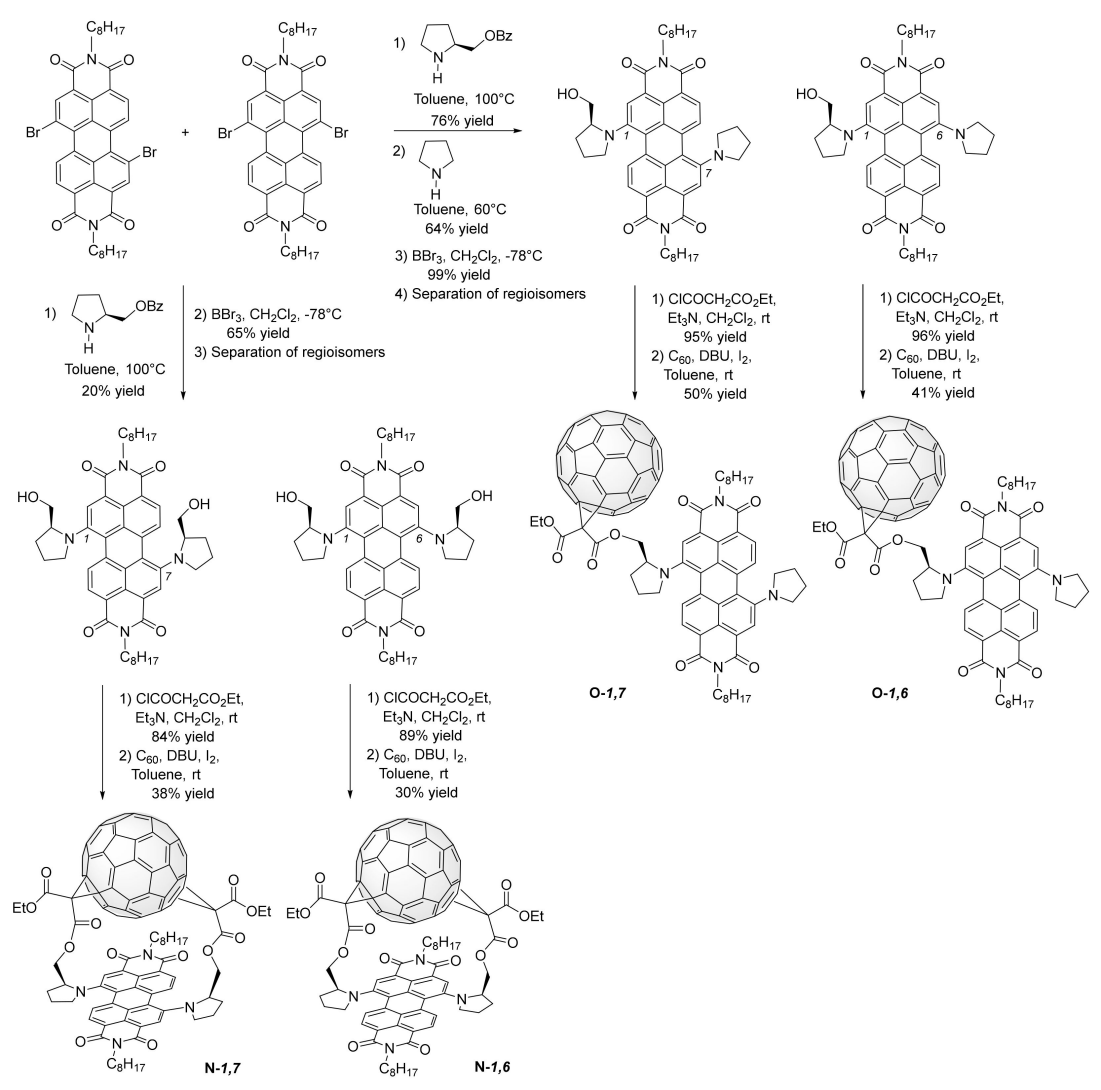
Synthesis of 1,6 and 1,7-regioisomers of single and double-bridged pyrrolidino-substituted PDI-C_60_ dyads **N** and **O**, respectively, by R.K. Dubey, H. Lemmetyinen and coll.

Starting from a mixture of 1,6/1,7 regioisomers of a dibromoPDI derivative, R.K. Dubey, H. Lemmetyinen and coll. conducted an original study of single and double-bridged pyrrolidino-substituted PDI-C_60_ dyads (Scheme 9) [78]. The first aromatic nucleophilic substitution using 2-(benzyloxymethyl)pyrrolidine (constructed from *L*-Proline) in toluene at 100 °C was found to be an efficient method for bay-PDI desymmetrization. A second reaction with pyrrolidine at 60 °C was followed by deprotection of the hydroxyl group by using BBr_3_. The 1,6- and 1,7-regioisomers could then be successfully separated by column chromatography. Each regioisomer was submitted to esterification with ethyl malonyl chloride and finally, C_60_ was linked using the modified Bingel reaction leading to single-bridged dyads **N-1,6** and **N-1,7** for this last crucial step. A similar synthetic methodology was applied to access double-bridged dyads **O-1,6** and **O-1,7** starting from a PDI backbone substituted by two (hydroxymethyl)pyrrolidine groups in relative bay positions. In contrast to the conventional C_60_ attachment at the imide position of dipyrrolidinyl-substituted PDIs presented for dyads **I** and **J** [63], it was clearly demonstrated that these mono- and bis-bay-functionalized PDIs have the advantage of keeping the two interacting moieties significantly much closer together. Due to the electron-donating character of pyrrolidinyl groups, single and double-bridged dyads **N** and **O** exhibited photoinduced electron transfer in a polar medium, and for single-bridged dyads even in a nonpolar medium. A detailed comparative study showed interesting differences in the physical properties of the 1,6- and 1,7-regioisomers for which different orientations of the donor-acceptor moieties were highlighted. Specifically, the single-bridged dyad **N-1,7** displayed highly efficient photoinduced electron transfer in a nonpolar medium, making it attractive for applications in solid OPV and optoelectronics. A P3HT:dyad **N-1,7**-based blend revealed a charge transfer process in thin film taking place firstly from P3HT to the PDI moiety to attain in a second time a charge-separated state P3HT^+^/PDI-C_60_^−^ before a recombination via hole transfer to the PDI moiety and finally a charge recombination to the ground state [79].

**Scheme 10:**
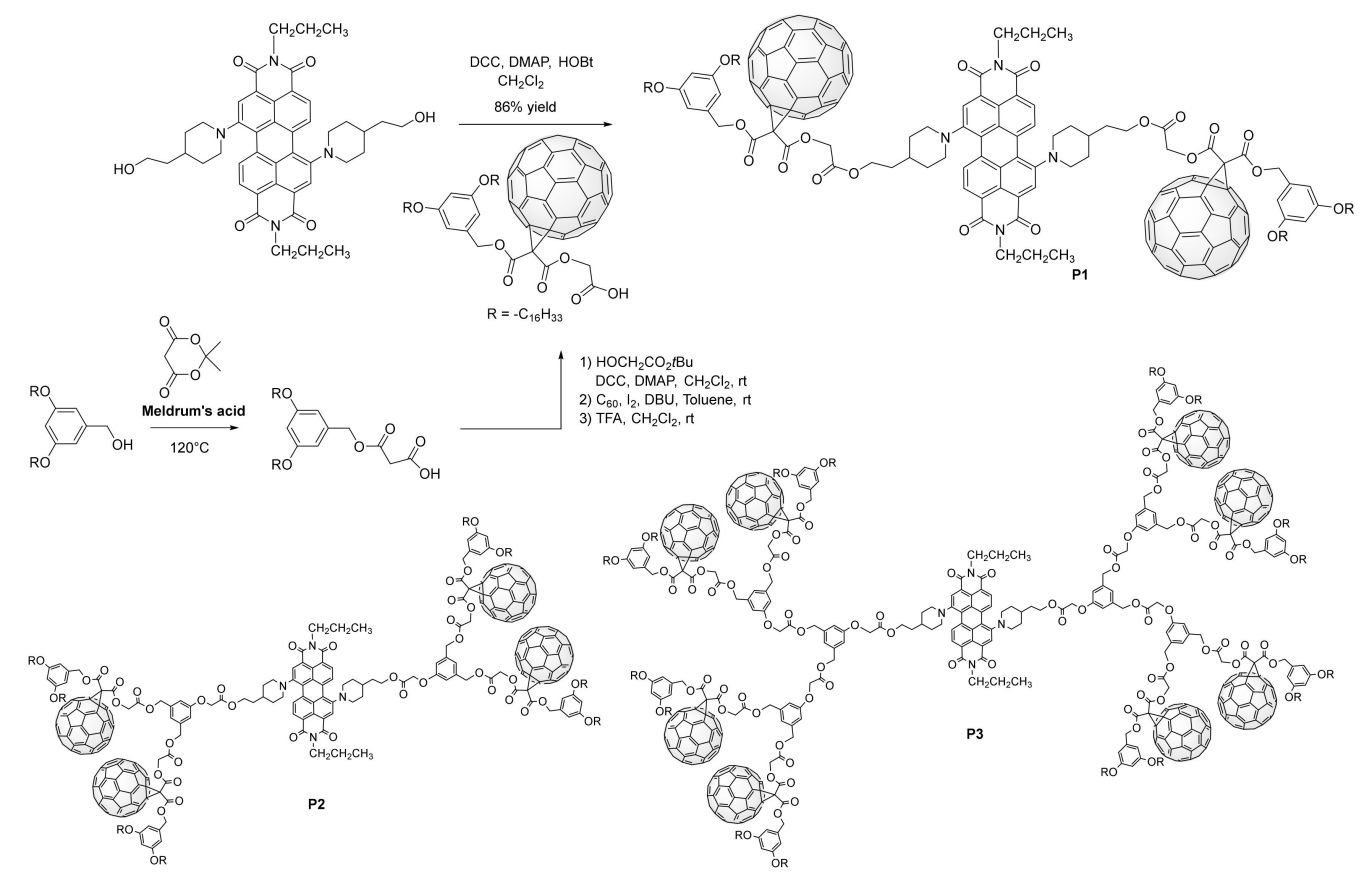
Synthesis of dendritic branches **P_1–3_** with peripheral fullerene subunits by J.-F. Nierengarten, N. Armaroli and coll.

One of the main advantages of the modified Bingel reaction [36,37] which uses malonate as starting material concerns the installation of ester moieties that allows additional chemical transformations. Thus, the key step in the synthesis of asymmetric malonate compounds is the use of Meldrum’s acid ring-opening with an alcohol to reach a monoesterified malonic acid derivative. This synthetic methodology was applied by J.-F. Nierengarten, N. Armaroli and coll. to synthesize three generations of fullerodendrimers with a PDI core **P_1–3_** (Scheme 10) [80]. Starting from the PDI dialcohol building block obtained by the nucleophilic substitution from 1,7-dibromoPDI [81], the esterification with fullerodendrons [82,83] bearing a carboxylic acid function led to the fullerodendrimers. Electrochemical studies showed that the oxidation of the dendrimers is always centered on the PDI core, while the first reduction always corresponds to fullerene units. Moreover, photophysical properties evidenced that a photosensitization process occurs within the dendrimers **P_1–3_** via photoinduced energy transfer leading to long-lived triplet states centered on a dendrimer core and capable of sensitizing singlet oxygen.

**Scheme 11:**
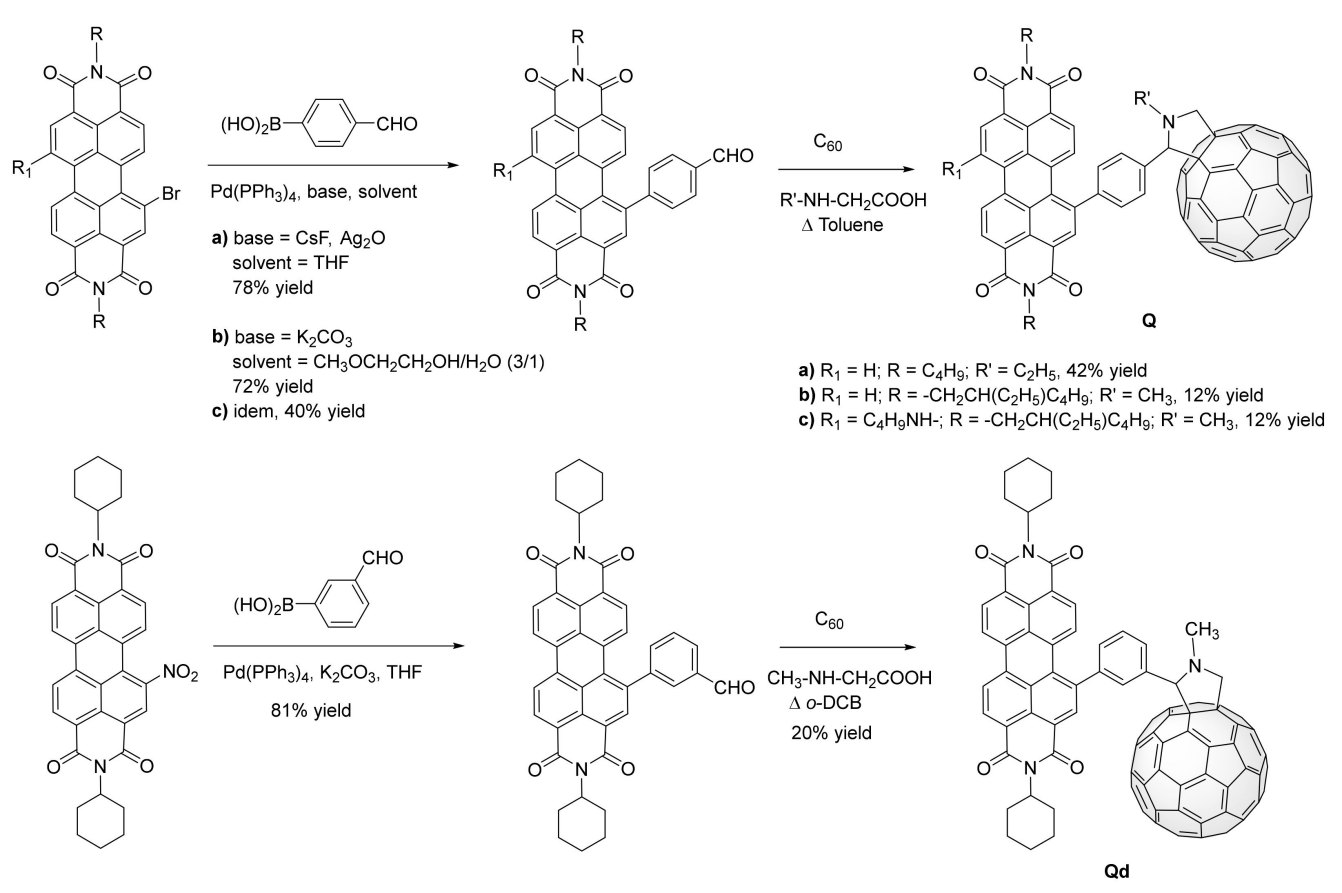
Suzuki–Miyaura coupling (SMC) and Prato reactions used for synthesis of dyad **Qa** by N. Champness and coll., dyads **Qb,c** by J. Zhao and coll. from bromoPDI, and dyad **Qd** by P. Hudhomme and coll. from nitroPDI.

Bromination at the PDI bay position gives access to one of the most valuable starting materials which, especially through palladium-catalyzed Suzuki-Miyaura, Stille or Sonogashira coupling reactions allows the preparation of substituted PDIs by building carbon–carbon (C-C) single bonds. Thus, Suzuki–Miyaura coupling (SMC) and Prato reactions were combined for the synthesis of dyad **Qa** by N.R. Champness and coll. (Scheme 11) [84]. Starting from mono-bromoPDI, an SMC reaction using 4-formylphenyl boronic acid in the presence of CsF, Ag_2_O, Pd(PPh_3_)_4_ in THF gave the corresponding aldehyde intermediate in 78% yield. Subsequent Prato reaction using C_60_ and *N*-ethylglycine in refluxing toluene afforded dyad **Qa** in 42% yield. It should be noted that a mixture of 1,6/1,7-dibromoPDI was used to attain the corresponding triad C_60_-PDI-C_60_. J. Zhao and coll. described the synthesis of dyads **Qb** and **Qc** using a similar strategy and described their photophysical properties for use as singlet oxygen photosensitizers for photooxidation of 1,5-dihydroxynaphthalene [85]. More recently, our group has demonstrated the possibility to use mono-nitroPDI and bis-nitroPDI for creating C-C single bonds using the SMC coupling reaction [86,87,88,89], and consequently dyad **Qd** was prepared by this original strategy [90].

## 3. Synthesis of a Platform for New C_60_-PDI Assemblies

**Figure 2:**
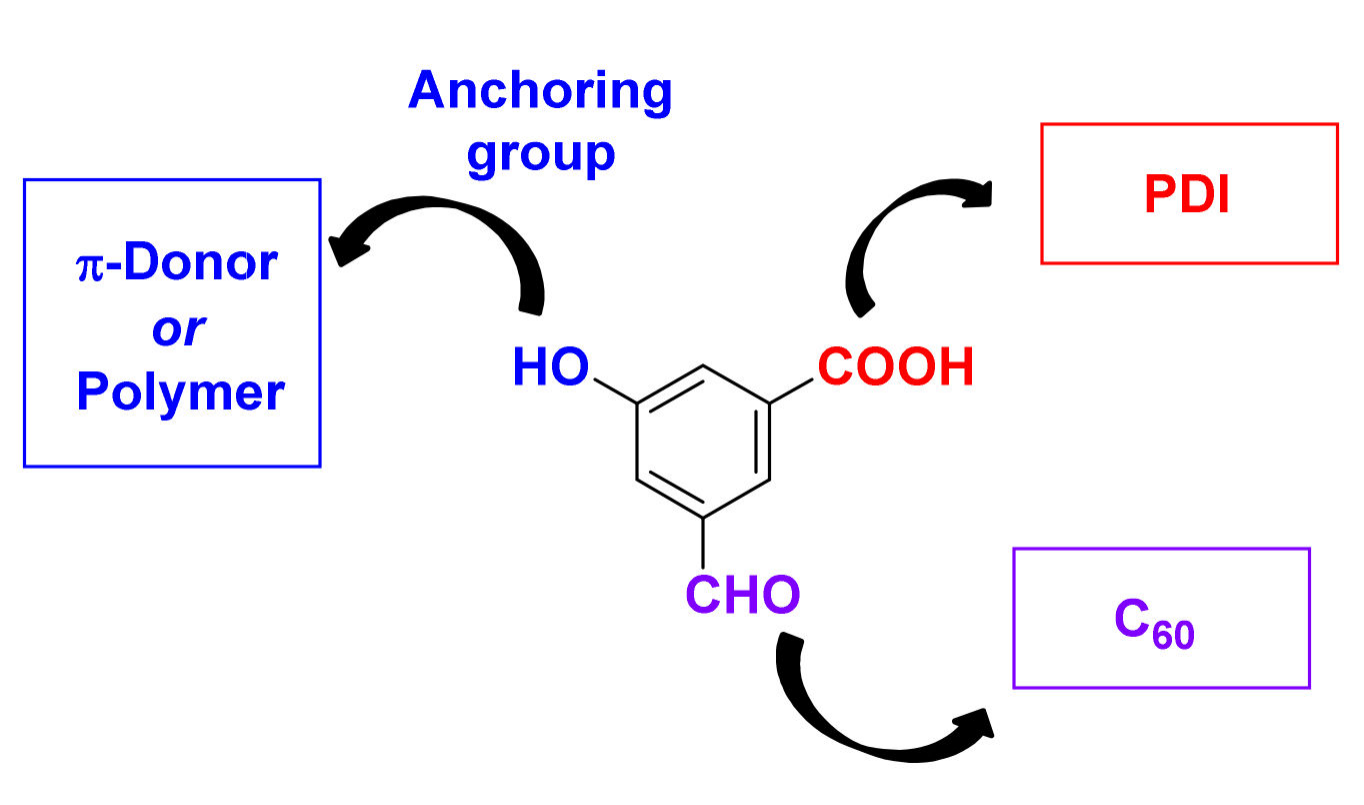
Presentation of the 3-formyl-5-hydroxybenzoic acid platform for incorporating C_60_ using Prato reaction, PDI moiety by esterification and the free phenol anchoring group for the versatile attachment of an electron donor or a polymerizable group.

While different C_60_-PDI dyads were developed in order to exploit their light-harvesting capabilities, to the best of our knowledge, no strategy has been investigated so far to introduce these chromophores onto a multifunctional platform allowing a post-functionalization. Consequently, we designed the 3-formyl-5-hydroxybenzoic acid as a platform incorporating functional groups for the introduction of both C_60_ and PDI acceptors, and a free anchoring group that can be used for the attachment of an electron donor or otherwise a polymerizable group according to the intended application (Figure 2). The design of this platform with substituents in the 1,3,5 positions was justified to minimize steric hindrance and to avoid possible mesomeric effects between the different groups.

**Scheme 12:**
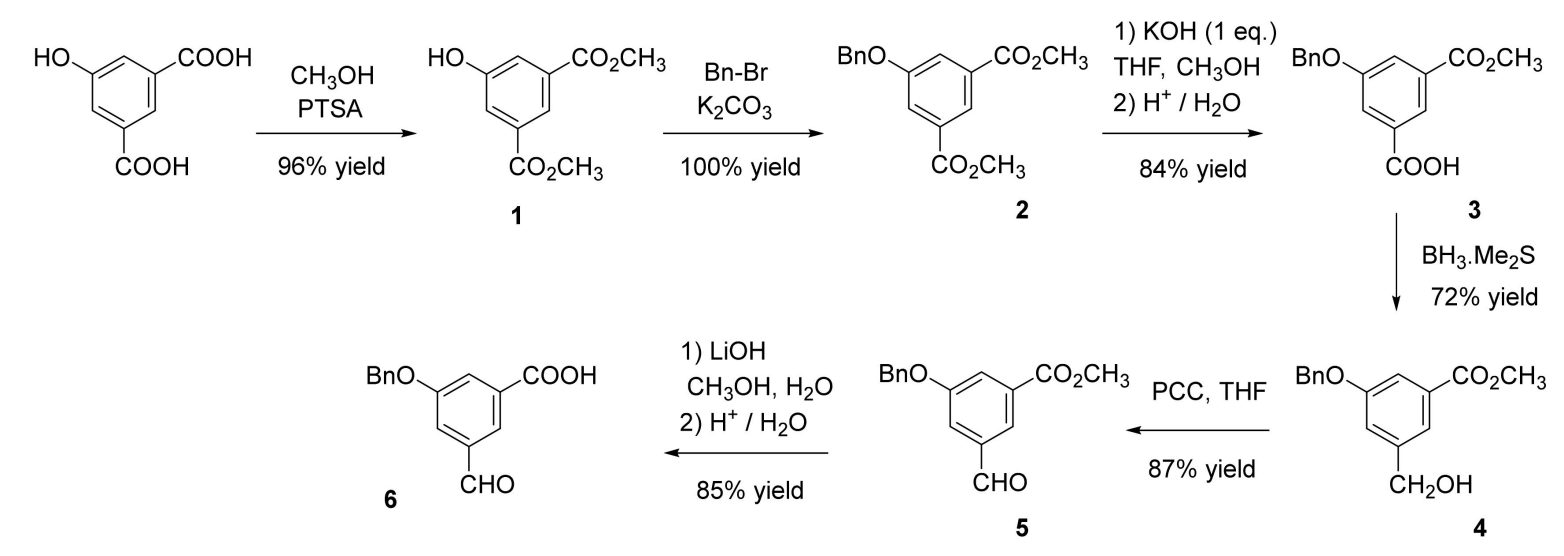
Synthesis of the benzyl protected 3-formyl-5-hydroxybenzoic acid derivative.

Building block 5-benzyloxy-3-formylbenzoic acid derivative **6** was synthesized in 6 steps in an overall 43% yield by improved modifications of a reported route (29% overall yield) for which experimental procedures were not described (Scheme 12) [91]. Esterification of commercially available 5-hydroxyisophthalic acid into diester **1** was followed by the protection of the phenol functionality into the benzyl group [92]. Monohydrolysis of resulting compound **2** using one equivalent of KOH in THF-MeOH [93] resulted after acidic treatment to key monoacid **3**. The selective reduction of the carboxylic group using the borane dimethylsulfide complex was followed by the oxidation step of primary alcohol **4** using PCC to obtain methyl 3-(benzyloxy)-5-formylbenzoate **5**. It is noteworthy to indicate that attempts for selective monoreduction of dimethyl isophthalate **2** into monoaldehyde **5** using DiBAl-H were unsuccessful. At last, treatment of compound **5** with LiOH in MeOH-H_2_O followed by acidic work-up afforded targeted building block **6**.

**Scheme 13:**
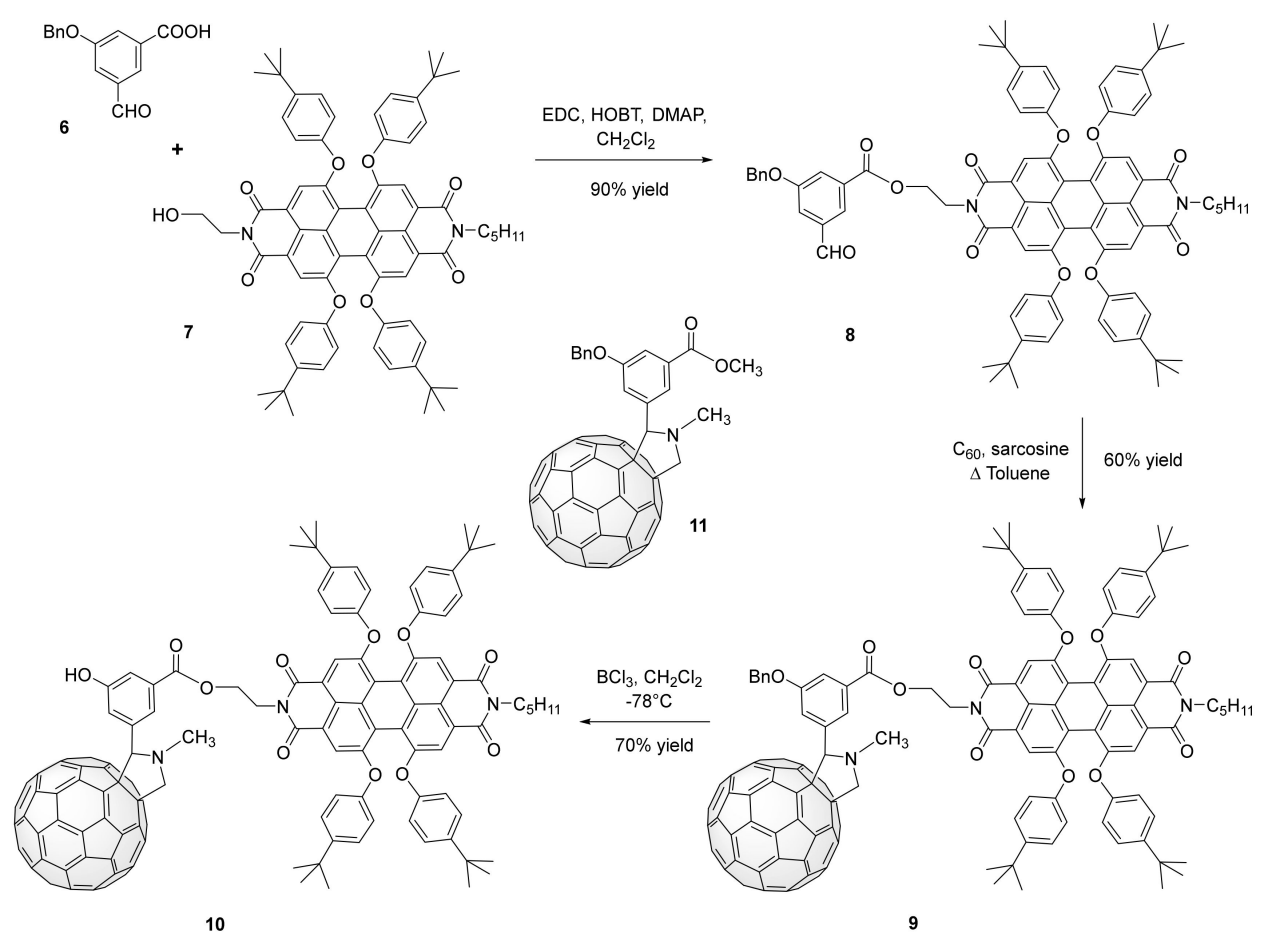
Synthesis of C_60_-PDI dyad **10**.

Both PDI and C_60_ units were grafted onto platform **6** by using the acid and formyl groups, respectively (Scheme 13). A Steglich type esterification [94] with PDI derivative **7**, prepared according to our previous report in 64% yield [48], and according to an esterification procedure, we have already experimented with fullerene chemistry [95], was achieved affording compound **8** in 90% yield. The subsequent 1,3-dipolar cycloaddition reaction was carried out using C_60_ and sarcosine in refluxing toluene to give, after purification by silica gel column chromatography, dyad **9** in satisfying 60% yield. Several methods were investigated for the deprotection of the benzyl group. Whereas classical hydrogenation using H_2_/Pd-C was unsuccessful, the reaction carried out using 47% solution HBr in the ionic liquid 1-butyl-3-methylimidazolium tetrafluoroborate (BMIM-BF_4_) [96] afforded dyad **10** in 21% yield. The best result was obtained by treating compound **9** with a solution of BCl_3_ [97] in CH_2_Cl_2_ at −78 °C leading to dyad **10** in 70% yield.

Dyads **9** and **10** were characterized by ^1^H and ^13^C NMR, MALDI-TOF mass spectrometry and for dyad **10** 2D-NMR analyses consisting of COSY, HMQC, and HMBC experiments were investigated (see Supplementary Materials). For dyad **9**, variable temperature NMR spectra recorded in CDCl_3_ showed a modification of the shape of one of the two characteristic perylene singlet signals when the temperature was increased to 328 K. Indeed, the ^1^H NMR spectrum at 298 K of dyad **10** showed for the perylene protons a well-defined singlet for two protons and a broad singlet for the two others. When recorded at 328 K, the two expected singlets at 8.20 and 8.24 ppm were correctly observed. Concerning the three protons of the aromatic platform, only one of these protons at 7.38 ppm was observed in the spectrum at 298 K. This phenomenon could result from the presence of both fullerene and PDI substituents around this aromatic platform. On the contrary, the spectrum recorded at 328 K showed a well-defined doublet of doublet at 7.40 ppm (^3^*J* = 1.5 and 2.4 Hz) accompanied by two broad singlets at 7.47 and 7.83 ppm. It is reasonable to consider that both protons correspond to those which are closer to bulky C_60_.

**Figure 3:**
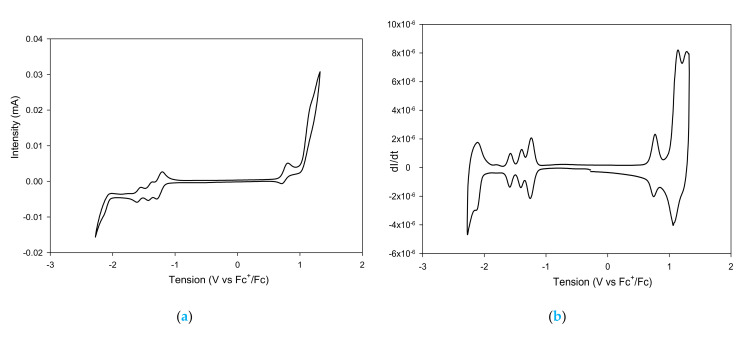
Cyclic voltammogram (**a**) and deconvoluted cyclic voltammogram (**b**) of dyad C_60_-PDI **9** (C = 5 × 10^−4^ M), Pt electrodes, CH_2_Cl_2_/*n*-Bu_4_NPF_6_ (0.1 M).

Electrochemical properties of dyad **9**, PDI **8**, and fulleropyrrolidine (FP) **11** references were analyzed by cyclic voltammetry in CH_2_Cl_2_ (or a mixture with CS_2_ in the case of compound **11**), in *n*-Bu_4_NPF_6_ 0.1 M solution as the supporting electrolyte (Table 1). The voltammograms have been deconvoluted in order to obtain the number of electrons involved in the processes. Dyad **9** showed three reversible reduction waves (Figure 3). The first two-electron process at E^o^_red1_ = −1.22 V (vs Fc^+^/Fc) was assigned to the formation of the C_60_^−^˙-PDI^−^˙ species. This process suggests that the first reduction wave of fullerene and the first reduction wave of the PDI are overlapping. This observation confirms that the type of linkage functionality onto C_60_ and between the two electroactive units slightly influences the redox potentials. Indeed, we have previously shown that, for the same substituted PDI derivative but anchored to a methanofullerene using an aliphatic linkage, four distinguishable reduction waves were observed [45,46]. In that case, the first reduction wave was attributed to the C_60_^−^˙-PDI species with 0.14 V of difference with the second reduction attributed to the C_60_^−^˙-PDI^−^˙ species. The second one-electron reduction process of dyad **9** corresponding to the formation of the C_60_^−^˙-PDI^2−^ species was at E^o^_red2_ = −1.38 V. The third one-electron reduction wave appearing at E^o^_red3_ = −1.55 V resulted from the formation of C_60_^2−^-PDI^2−^. A first reversible two-electron oxidation process arises at *E^o^_ox1_* = +0.81 V. Moreover, comparison of these different values for dyad **9** with reference compounds **8** and **11** suggests that there is no significant interaction taking place between both electroactive moieties in the ground state.

The optical properties of PDI **7** and dyad **9** were analyzed in CH_2_Cl_2_ solutions by UV-vis absorption (*ca*. 10^−5^ M) and photoluminescence emission (*ca*. 5 × 10^−6^ M) spectroscopy (see Supplementary Materials). Reference PDI **7** and dyad **9** are characterized by the three characteristic absorption bands of the PDI core at around 450, 540, and 580 nm, and the maximum absorption of dyad **9** at 229 nm is ascribed to the fullerene moiety. The UV–vis spectrum of PDI-C_60_ dyad **9** corresponds to the linear combination of PDI and C_60_ absorption spectra suggesting the absence of electronic coupling in the ground state between the PDI and fullerene moieties. A quasi-quantitative quenching of fluorescence was observed for dyad **9**, due to the strong interaction, probably energy transfer according to results on our previous reported systems [46,49], between the PDI and C_60_ in the excited state (Table 2).

We propose here a new polymeric architecture involving together PDI and C_60_ units on a non-conductive side-chain polymethacrylate in which C_60_-PDI dyads are attached as pendant moieties onto the main chain of the polymer. From the literature, with the aim of fabricating well-ordered nanostructured BHJ photovoltaic devices presenting a large donor/acceptor interface in an all-polymers strategy, M. Thelakkat and coll. have synthesized unsymmetrical poly(PDI-acrylate) (PPDI-Acr) [98] and a series of PPDI-Acr-based diblock copolymers [99,100,101,102]. The PDI-Acr monomer was also used as a building block to prepare polymers and donor–acceptor diblock copolymers with PDI pendant groups for applications in organic field-effect transistors (OFETs) [103,104,105]. Poly(methacrylate)s labeled with PDI derivatives were also described as long-wavelength absorbing dyes [106]. It should be noted that pendant PDI-*tert*-butyl acrylate copolymers have shown efficient energy transfer within PDI luminophores in the polymer suggesting potential use in luminescent solar concentrators [107]. Concerning the C_60_ counterpart, only a few poly(methyl methacrylates) containing covalently attached fullerene C_60_ were synthesized [108,109,110]. In particular, it was shown that the fullerene attached to the branched poly(methyl methacrylate) could act as an inhibitor of the cross-linking radical polymerization of dimethacrylate.

**Scheme 14:**
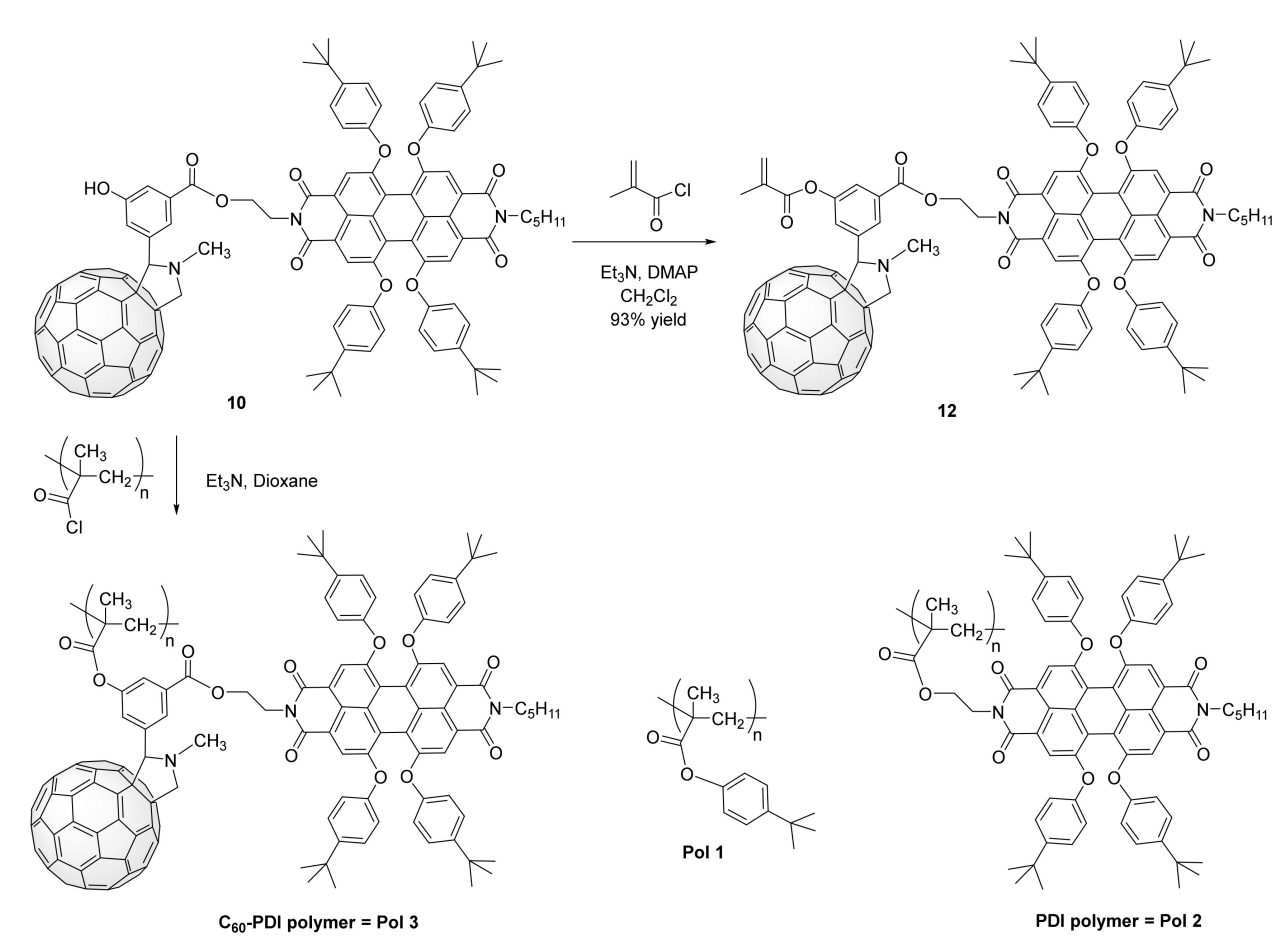
Synthesis of dyad **12**, C_60_-PDI polymer **Pol 3**, and PDI-based polymer **Pol 2**, *tert*-butylphenol based polymer **Pol 1** for references.

To demonstrate the ability of the phenolic group of platform **6** to be functionalized, the reaction of dyad **10** with methacryloyl chloride was successfully carried out in the presence of triethylamine and 4-dimethylaminopyridine (DMAP) in CH_2_Cl_2_ (Scheme 14). This result clearly demonstrated that the steric hindrance suspected around the platform due to the presence of both C_60_ and PDI was not a limiting factor to a post-functionalization process. This dyad **12** was characterized by ^1^H and ^13^C NMR and exhibited the molecular peak at *m*/*z* = 2059 by MALDI-TOF mass spectrometry.

For the synthesis of polymer 3 (**Pol 3**), we have opted for an addition-elimination reaction carried out on dyad **10** with commercial poly(methacryloyl chloride) (25% solution in dioxane, Polyscience) in dry dioxane in the presence of triethylamine. Indeed, although a co-polymerization of dyad **12** and methyl methacrylate could be considered [111], C_60_ acting as a radical scavenger during the free-radical polymerization [112], we thought that homopolymerization of dyad **12** could be difficult to occur due to the volume of the substituent of the methacrylate unit which determines the penultimate-unit effect during the free-radical polymerization or co-polymerization process [113].

Two other polymers were prepared as references, starting from PDI derivative **7** and *tert*-butylphenol giving **Pol 2** and **Pol 1**, respectively. Characterization by IR spectroscopy of the obtained polymers showed the total disappearance of the υ_OH_ vibration band of the aliphatic alcohol or phenolic function of starting materials. The characteristic signals of the ester vibrations υ_C=O_ = 1748 cm^−1^ and υ_C-O_ = 1204–1014 cm^−1^ were shown for reference polymer **Pol 1**. These vibration bands υ_C=O_ = 1759 cm^−1^ (ester) and υ_C-O_ = 1340–1100 cm^−1^ were also observed for **Pol 2** and **Pol 3**, with the additional vibration band at υ_C=O_ = 1654 cm^−1^ of the imide groups arising from the PDI skeleton.

**Figure 4:**
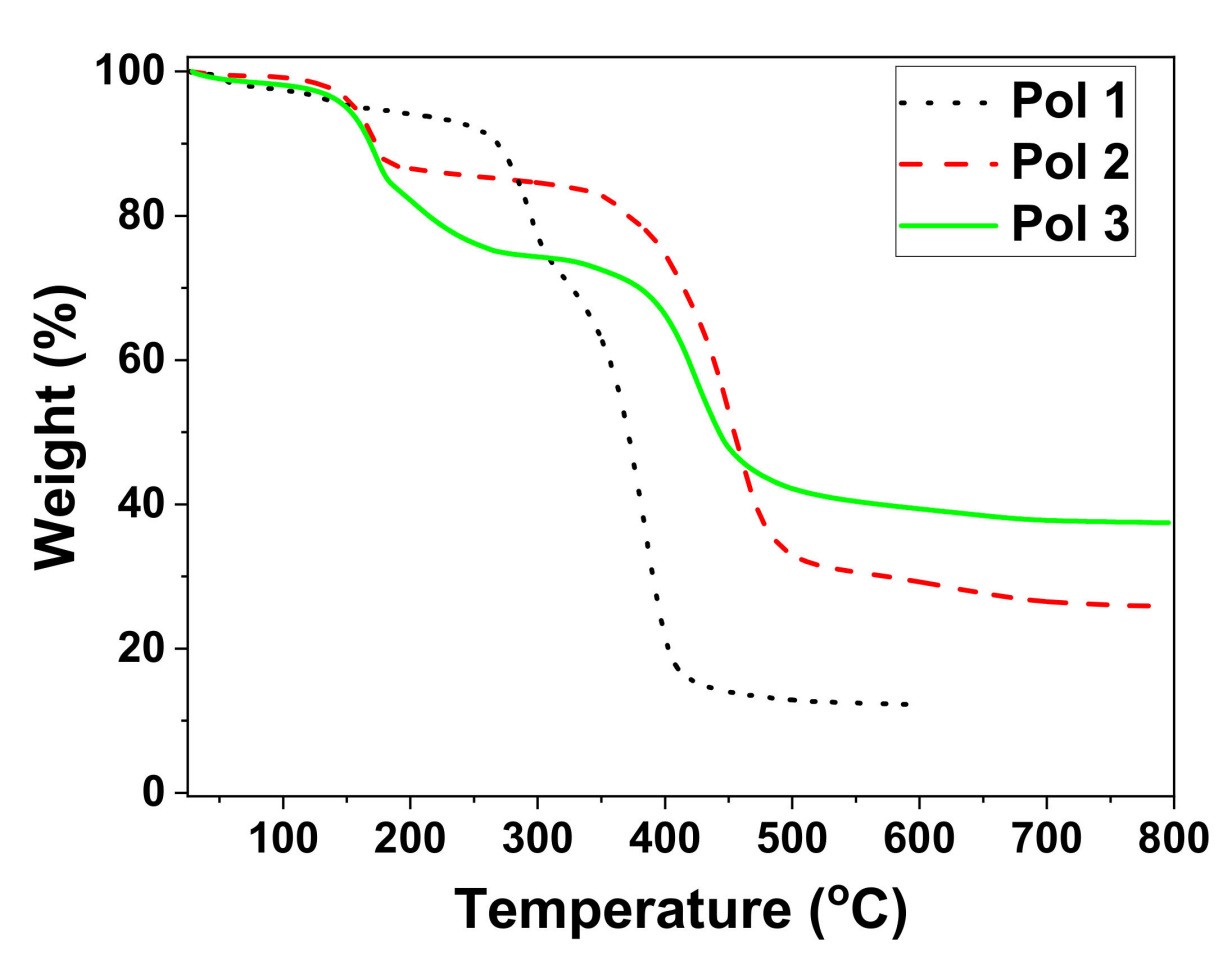
TGA spectra of **Pol 1** (dotted line), **Pol 2** (dashed line) and **Pol 3** (full line) recorded by a heating rate of 10 °C min^−1^ under N_2_ atmosphere.

Differential scanning calorimetry (DSC) was used for the determination of the glass transition temperature (T_g_) value of C_60_-PDI polymer **Pol 3** which is equal to 151 °C. This has to be compared with the T_g_ values of 59 °C and 105 °C for **Pol 1** and poly(methyl methacrylate) (PMMA), respectively. This higher T_g_ value can be explained by several interconnected factors such as the steric hindrance which could reduce the degree of substitution on the polymeric chain. Moreover, unreacted acyl chloride groups also undergo some hydrolysis in acid groups which leads to an increase in the T_g_ as a result of hydrogen bond interaction that reduces the mobility. Moreover, although the substituent corresponding to dyad **10** represents a bulky group, it can also form aggregates due to π-π stacking interactions which limit the mobility of the polymer segment chains, thus explaining the higher T_g_ value of **Pol 3** compared to PMMA. Moreover, the thermogravimetric analysis (TGA) clearly showed the higher stability of **Pol 3** composed of both PDI and C_60_ units (Figure 4).

The concentration of dye vs. dyad in the polymer samples was determined using a colorimetric technique employing Lambert Beers’ law. A calibration curve was obtained by plotting the absorbance against the concentration of dye solutions of known concentrations. The technique allowed the determination of a 73% (weight ratio, 18% molar ratio) content of perylene dye in the PDI polymer **Pol 2** used as a reference sample. The same approach was applied for C_60_-PDI dyad polymer **Pol 1** resulting in a 62% weight ratio, with a 6.5% molar ratio of dyad content (see Supplementary Materials).

## 4. Conclusions

In conclusion, our first objective was to establish the state-of-the-art of the different synthetic methodologies described to date to prepare C_60_ and PDI-based assemblies focusing on the two possibilities for attachment of C_60_ on the imide or bay PDI positions. Secondly, we report here a straightforward and versatile method to prepare an aromatic platform bearing three different phenol, aldehyde, and carboxylic acid functional anchors. This platform was employed for the attachment of C_60_ and PDI dye and then took advantage of the free phenol function to prepare original methacrylate monomer and pendant poly(methacrylate) macromolecules. A complete and detailed investigation of the photophysical properties of this polymer is currently in progress. This synthetic approach appears very promising for the preparation of different triads since functionalities such as π-donors for example can be introduced on the phenolic anchor site in the last synthetic step.

## 5. Experimental Procedures

*Dimethyl 5-hydroxyisophthalate 1:*

A solution of commercial 5-hydroxyisophthalic acid (6.34 g, 0.035 mol) and *p*-toluenesulfonic acid (1.21 g, 0.007 mol) in methanol (120 mL) was stirred at reflux for 24 h. The solvent was partially removed in vacuo and after the addition of diethyl ether, the solution was neutralized using an aqueous sodium hydroxide solution. The resulting organic layer was washed with water, dried over MgSO_4_, then concentrated in vacuo. The product (6.97 g) was obtained as a white powder in 96% yield. Spectroscopic data are in agreement with the literature [92]. ^1^H NMR (acetone-*d*_6_, 300 MHz) *δ*: 9.20 (s, 1H, OH), 8.10 (t, *J* = 1.5 Hz, 1H, PhH), 7.68 (d, *J* = 1.5 Hz, 2H, PhH), 3.90 ppm (s, 6H, OCH_3_).

*Dimethyl 5-(benzyloxy)isophthalate 2:*

A solution of dimethyl 5-hydroxyisophthalate **1** (5.8 g, 0.027 mol) and K_2_CO_3_ (5.7 g, 0.041 mol) in anhydrous acetone (120 mL) was degassed with N_2_ before the addition of benzyl bromide (3.4 mL, 0.027 mol). The reaction mixture was stirred at reflux under N_2_ for 24 h. Acetone was removed in vacuo and the remaining solid was dissolved in ethyl acetate and then washed with water. The aqueous layer was extracted with ethyl acetate, and the organic layer was washed with brine, dried over MgSO_4_, and concentrated in vacuo to give compound **2** as a white solid (8.25 g, 100%). Spectroscopic data are in agreement with the literature [92,93]. ^1^H NMR (acetone-*d*_6_, 300 MHz) *δ*: 8.20 (t, *J* = 1.5 Hz, 1H, PhH), 7.83 (d, *J* = 1.5 Hz, 2H, PhH), 7.55–7.35 (m, 5H, OPhH), 5.28 (s, 2H, OCH_2_), 3.92 ppm (s, 6H, OCH_3_).

*3-(Benzyloxy)-5-(methoxycarbonyl) benzoic acid 3:*

Potassium hydroxide, 85% in pellets (0.84 g, 0.013 mol) was added to a solution of dimethyl 5-(benzyloxy)isophthalate **2** (4 g, 0.013 mol) in a mixture of methanol (60 mL) and THF (30 mL). The reaction mixture was stirred at reflux under N_2_ overnight. The solvent was removed in vacuo, and the remaining slurry was dissolved in water and washed with CH_2_Cl_2_. Concentrated HCl was added to the aqueous layer to reach pH = 3. The resulting white precipitate was collected by vacuum filtration and dried to give compound **3** as a white solid (3.2 g, 84%). Spectroscopic data are in agreement with the literature [92]. ^1^H NMR (acetone-*d*_6_, 300 MHz) *δ*: 8.24 (m, 1H, PhH), 7.86 (m, 1H, PhH), 7.86 (m, 1H, PhH), 7.83 (m, 1H, PhH), 7.55–7.34 (m, 5H, OPhH), 5.28 (s, 2H, OCH_2_), 3.92 (s, 3H, OCH_3_).

*Methyl 3-(benzyloxy)-5-(hydroxymethyl)benzoate 4:*

BH_3_(CH_3_)_2_S complex (2.0 M in THF, 4.2 mL, 8 mmol) was added dropwise to a solution of compound **3** (2 g, 7 mmol) in anhydrous THF (25 mL) at 0 °C. The mixture was then stirred at reflux overnight. The reaction was quenched and neutralized by the addition of an H_2_O/CH_3_COOH (1:2) solution (6 mL). The solvents were removed in vacuo and the slurry was dissolved in ethyl acetate, washed successively with a 30% K_2_CO_3_ aqueous solution, then 1N HCl aqueous solution, saturated NaHCO_3_ aqueous solution, and brine. After drying over anhydrous Na_2_SO_4_, the solution was then concentrated. The product was purified by silica gel column chromatography using petroleum ether and ethyl acetate (4:1) as the mixture of eluents. The product was obtained as colorless oil (1.36 g, 72%) which crystallized by freezing. Spectroscopic data are in agreement with the literature [92]. ^1^H NMR (acetone-*d_6_*, 300 MHz) *δ*: 7.62 (m, 1H, PhH), 7.52–7.28 (m, 7H, PhH), 5.18 (s, 2H, OCH_2_), 4.68 (d, *J* = 5.7 Hz, 2H, OCH_2_), 4.40 (t, *J* = 5.7 Hz, 1H, OH), 3.86 (s, 3H, OCH_3_).

*Methyl 3-(benzyloxy)-5-formylbenzoate 5:*

A solution of pyridinium chlorochromate (2.77 g, 0.013 mol) in THF (25 mL) was added to a solution of compound **4** (2.8 g, 0.011 mol) in THF (25 mL). The reaction mixture was heated at reflux for 24 h. After dilution with CH_2_Cl_2_ (30 mL), the solution was filtered through celite, washed with a saturated NH_4_Cl solution and water, dried over MgSO_4_, and concentrated in vacuo. The residue was purified by silica gel column chromatography using petroleum ether and ethyl acetate (4:1) as the mixture of eluents and compound **5** was isolated as a white precipitate (2.41 g, 87%). Spectroscopic data are in agreement with the literature [92]. ^1^H NMR (acetone-*d_6_*, 300 MHz) *δ*: 10.09 (s, 1H, CHO), 8.12 (t, *J* = 1.5 Hz 1H, PhH), 7.88 (dd, *J* = 1.2 Hz and 1.5 Hz, 1H, PhH), 7.77 (dd, *J* = 1.2 Hz and 1.5 Hz, 1H, PhH), 7.55–7.35 (m, 5H, PhH), 5.31 (s, 2H, OCH_2_), 3.93 ppm (s, 3H, OCH_3_).

*3-(Benzyloxy)-5-formylbenzoic acid 6:*

To a solution of compound **5** (0.6 g, 2.2 mmol) in methanol and water (48 mL, 3:1) was added lithium hydroxide (0.1 g, 4.4 mmol). The mixture was stirred at room temperature overnight and most of the methanol was removed in vacuo. The aqueous solution was diluted and washed with CH_2_Cl_2_. The solution was acidified until pH = 3 using an HCl aqueous solution. The precipitate was collected by filtration and dried to give compound **6** as a white solid (0.48 g, 85%). Spectroscopic data are in agreement with the literature [92]. ^1^H NMR (acetone-*d_6_*, 300 MHz) *δ*: 10.09 (s, 1H, CHO), 8.15 (t, *J* = 1.5 Hz, 1H, PhH), 7.91 (dd, *J* = 1.5 Hz and 2.7 Hz, 1H, PhH), 7.77 (dd, *J* = 1.5 Hz and 2.7 Hz, 1H, PhH), 7.56–7.35 (m, 5H, PhH), 5.31 (s, 2H, OCH_2_). ^13^C NMR (Acetone-*d*_6_, 75 MHz) *δ*:193.2; 167.4; 161.4; 140.3; 138.6; 134.9; 130.4; 129.9; 129.6; 125.3; 123.41; 120.1; 72.1.

*Compound***7**:

A mixture of *N*-2′-Hydroxyethyl-*N*’-pentyl-1,6,7,12-tetrakischloroperylene- 3,4:9,10-*bis*(dicarboximide) [48] (860 mg, 1.4 mmol), *p*-tertiobutylphenol (2.1 g, 14 mmol) and K_2_CO_3_ (2 g, 14.5 mmol) in anhydrous *N*-methylpyrrolidone (NMP) (50 mL) was stirred at 130 °C under argon for 14 h. After being cooled to room temperature, the reaction mixture was poured into HCl solution (1N) (50 mL). The precipitate was collected by filtration, washed thoroughly with water until neutrality, and then purified by column chromatography on silica gel using successively CH_2_Cl_2_ and then CH_2_Cl_2_/acetone (30:1) as the mixture of eluents. Crystallization from CH_2_Cl_2_/methanol afforded compound **7** as a purple powder which was dried overnight in vacuum at 80 °C (975 mg, 64% yield). ^1^H NMR (CDCl_3_, 300 MHz) *δ*: 8.26 (s, 2H), 8.24 (s, 2H), 7.28–7.23 (m, 8H), 6.86–6.83 (m, 8H), 4.41 (t, ^3^*J* = 7 Hz, 2H), 4.12 (broad t, ^3^*J* = 7 Hz, 2H), 3.95 (t, ^3^*J* = 7 Hz, 2H), 2.2 (broad s, 1H), 1.70 (broad t, 2H), 1.40–1.35 (m, 4H), 1.31 (s, 36H), 0.90 (t, ^3^*J* = 7 Hz, 3H). ^13^C NMR (75 MHz, CDCl_3_) δ 164.4, 163.6, 156.3, 156.0, 153.0, 152.9, 147.5, 147.4, 133.1, 133.0, 126.8, 122.8, 122.1, 121.1, 120.4, 119.9, 119.7, 119.5, 119.4, 77.4, 61.9, 43.0, 40.8, 34.5, 31.6, 29.3, 27.9, 22.5, 14.1.

*Compound***8**:

To a degassed solution of 3-(benzyloxy)-5-formylbenzoic acid **6** (100 mg, 0.390 mmol) in anhydrous CH_2_Cl_2_ (30 mL) was added hydroxybenzotriazole (HOBt) (53 mg, 0.390 mmol), *N*-(3-dimethylaminopropyl)-*N*′-carbodiimide (EDC) (82 µL, 0.429 mmol), 4-dimethylaminopyridine (48 mg, 0.39 mmol) and compound **7** (428 mg, 0.39 mmol). The mixture was stirred at room temperature under argon for 5 days. The solvent was removed under reduced pressure and the residue was purified by silica gel column chromatography using CH_2_Cl_2_/acetone (30:1) as the mixture of solvents. Compound **8** was isolated as a purple powder (470 mg, 90%). ^1^H NMR (CDCl_3_, 500 MHz) *δ*: 9.89 (s, 1H), 8.23 (s, 2H), 8.20 (s, 2H), 7.99 (t, *J* = 1.5 Hz, 1H), 7.83 (dd, *J* = 1.5 Hz and 2.7 Hz, 1H, PhH), 7.61 (dd, *J* = 1.5 Hz and 2.7 Hz, 1H, PhH), 7.38–7.28 (m, 5H), 7.24 and 7.23 (2d, *J* = 9 Hz, 8H), 6.82 and 6.81 (2d, *J* = 8.7 Hz, 8H), 5.07 (s, 2H), 4.66–4.55 (br t, 4H), 4.12–4.07 (br t, 2H), 1.66 (br t, 2H), 1.35 (m, 4H), 1.29 (s, 18H), 1.28 (s, 18H), 0.87 (t, *J* = 7.2 Hz, 3H). ^13^C NMR (CDCl3, 125 MHz) δ:191.0; 165.1; 163.5; 163.4; 159.2; 156.1; 155.8; 152.9; 152.7; 147.3; 147.2; 137.8; 135.8; 133.0; 132.8; 128.6; 128.2; 127.5; 126.7; 122.6; 122.0; 121.0; 120.2; 119.4; 119.1; 77.2; 70.4; 34.6; 34.4; 31.4; 31.6; 29.2; 22.4; 13.9. MS (MALDI-TOF, pos. mode, dithranol): *m*/*z*: calcd for: 1334.59; found: 1334.6 [M]^+^ HRMS (ESI FAB+): calcd for C_86_H_82_N_2_O_12_: 1334.586777; found: 1334.5865. Elemental analysis for C_86_H_82_N_2_O_12_ (1335.58) calcd. C 77.34 H 6.19 N 2.10; found C 77.13, H 6.40, N 2.00.

*Dyad***9**:

To a solution of compound **8** (250 mg, 0.18 mmol) in anhydrous toluene (70 mL) were added C_60_ (200 mg, 0.27 mmol) and N-methylglycine (sarcosine) (60 mg, 0.67 mmol). The reaction mixture was refluxed under nitrogen for 24 h. After the solution was cooled to room temperature, the solvent was removed under reduced pressure and the residue was purified by silica gel column chromatography using CS_2_ as the eluent to remove the excess C_60_ with CS_2_, then toluene/ethyl acetate (100:1) as the mixture of solvents. Dyad **9** was isolated as a purple powder (225 mg, 60%). ^1^H NMR (CDCl_3_, 500 MHz, 298 K) *δ*: 8.26 (br s, 2H), 8.19 (s, 2H), 7.58 (br s, 1H), 7.38–7.28 (m, 4H), 7.21–7.17 (m,10H), 6.77 (m, 8H), 5.05 (br s, 2H), 4.90 (d, *J* = 10 Hz, 1H), 4.77 (s, 1H), 4.62 (br s, 2H), 4.41 (br s, 1H), 4.16 (br s, 1H), 4.12 (br t, 2H), 2.67 (s, 3H), 1.70 (br t, 2H), 1.35 (m, 4H), 1.27 (s, 18H), 1.26 (s, 18H), 0.89 (t, 3H). At 298 K, the signal corresponding to two protons of the PDI at 8.26 ppm is not well resolved. A temperature-dependent NMR experiment showed perfectly the singlet at 328 K. ^1^H NMR (CDCl_3_, 500 MHz, 328 K): δ = 8.26 (s, 2H), δ = 8.19 (s, 2H). ^13^C NMR (CDCl_3_, 125 MHz) *δ*: 166.2, 163.6, 163.5, 156.3, 155.6, 153.3, 153.0, 147.4, 147.3, 147.2, 146.4, 146.1, 146.0, 145.8, 145.4, 145.3, 145.1, 144.4, 142.7, 142.6, 142.2, 142.1, 142.0, 141.7, 139.4, 136.7, 136.5, 135.7, 133.1, 128.7, 128.2, 127.7, 126.8, 126.7, 122.7, 122.1, 121.5, 120.8, 120.6, 120.0, 119.5, 119.2, 83.0, 74.0, 70.0, 69.0, 40.8, 40.0, 34.5, 31.6, 29.4, 28.0, 22.6, 14.1. MS (MALDI-TOF, pos. mode, dithranol): *m*/*z*: calcd for: 2081.53; found: 2082.5 [M]^+^ HRMS (ESI FAB+): calcd for C_148_H_87_N_3_O_11_: 2081.634062; found: 2082.6468. Elemental analysis for C_148_H_87_N_3_O_11_ (2083.29) calcd. C 85.33 H 4.21 N 2.02; found C 82.99, H 4.27, N 1.98.

*Dyad***10**:

Experimental procedure using HBr/BMIM-BF_4_:

Dyad **9** (100 mg, 0.048 mmol) and concentrated hydrobromic acid (47%, 0.96 mmol) in 1-*n*-butyl-3-methylimidazolium tetrafluoroborate (BMIM-BF_4)_ (1.5 mL) were stirred at 115 °C for 36 h. The reaction mixture was extracted with CH_2_Cl_2_, washed with water, dried on MgSO_4_, and concentrated in vacuo. Purification of the residue by silica gel column chromatography using CH_2_Cl_2_/acetone (30:1) as the mixture of solvents furnished a purple powder (20 mg, 21%).

Experimental procedure using BCl_3_:

To a solution of dyad **9** (372 mg, 0,179 mmol) in anhydrous CH_2_Cl_2_ (40 mL), cooled to −78 °C under argon, was added BCl_3_ 1M in CH_2_Cl_2_ (3.5 mL). After stirring at −78 °C for 2 h, methanol (1 mL) was slowly added and the temperature was left to reach room temperature. The solvent was removed under reduced pressure and this cycle of methanol addition and evaporation was repeated 3 times. The mixture was then neutralized with 28% ammonia solution. After extraction with CH_2_Cl_2_ and concentration in vacuo, the residue was purified by silica gel column chromatography using CH_2_Cl_2_/acetone 30:1 as the mixture of solvents. Dyad **10** was precipitated using CH_2_Cl_2_/Petroleum ether and isolated as a purple powder (250 mg, 70%). ^1^H NMR (CDCl_3_, 500 MHz, 298 K) *δ*: 8.21 (br s, 2H, H perylene), 8.19 (s, 2H, H perylene), 7.39 (br s, 1H, Ha or Hb or Hc), 7.20 and 7.19 (2d, ^3^*J* = 8.5 Hz, 8H, H_D_ or H_E_), 6.78 and 6.76 (2d, ^3^*J* = 8.5 Hz, 8H, H_D_ or H_E_), 6.03 (br s, 1H, OH), 4.88 (d, *J* = 9.5 Hz, 1H, -NCH_2_CH_2_O-), 4.76 (s, 1H, CH pyrrolidine), 4.69 (br s, 1H, -NCH_2_CH_2_O-), 4.58 (br s, 2H, CH_2_ pyrrolidine), 4.45 (br s, 1H, -NCH_2_CH_2_O-), 4.16 (br s, 1H, -NCH_2_CH_2_O-), 4.10 (t, *J* = 7.5 Hz, 2H, NCH_2_(CH_2_)_3_CH_3_), 2.68 (s, 3H, N-CH_3_), 1.67 (m, 2H, -CH_2_-), 1.37 (m, 4H, -CH_2_CH_2_-), 1.28 (s, 36H, *t*Bu), 0.90 (t, *J* = 7 Hz, 3H, CH_3_).

**Figure 5:**
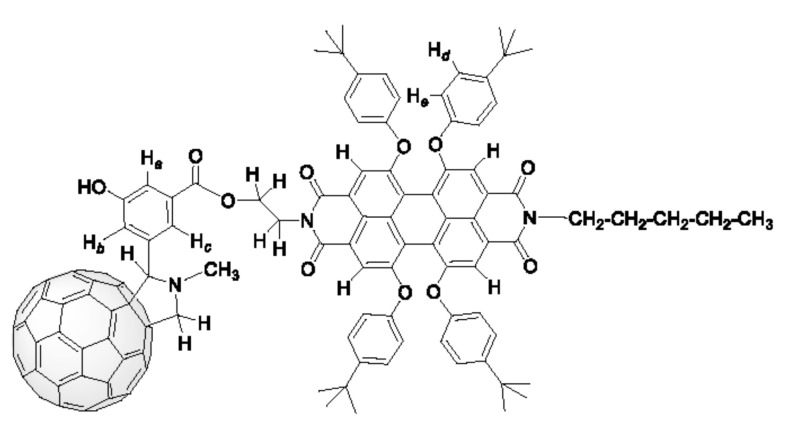
Formula of dyad **10** for ^1^H NMR spectrum analysis.

Two protons of the aromatic platform were not characterized on the spectrum at 298 K. This problem was solved by recording the ^1^H NMR spectrum at 328 K. ^1^H NMR (CDCl_3_, 300 MHz, 328 K) *δ*: 8.24 (s, 2H, H perylene), 8.20 (s, 2H, H perylene), 7.83 (br s, 1H, Ha or Hb or Hc), 7.47 (br s, 1H, Ha or Hb or Hc), 7.40 (dd, 1H, ^3^*J* = 1.5 and 2.4 Hz, Ha or Hb or Hc), 7.21 (d, ^3^*J* = 9 Hz, 8H, H_D_ or H_E_), 6.78 and 6.81 (2d, ^3^*J* = 9 Hz, 8H, H_D_ or H_E_), 5.57 (br s, 1H, OH), 4.89 (d, *J* = 9.3 Hz, 1H), 4.78 (s, 1H, CH pyrrolidine), 4.60 (m, 4H), 4.14 (m, 3H), 2.68 (s, 3H, N-CH_3_), 1.71 (m, 2H, -CH_2_-), 1.39 (m, 4H, -CH_2_CH_2_), 1.29 and 1,30 (2s, 36H, *t*Bu), 0.91 (t, *J* = 7 Hz, 3H, CH_3_) (Figure 5). ^13^C NMR (CDCl_3_, 75 MHz) *δ*: 165.9, 163.5, 163.4, 156.1, 155.7, 155.6, 153.0, 152.8, 147.2, 147.1, 146.2, 146.1, 146.0, 145.9, 145.8, 145.7, 145.6, 145.5, 145.3, 145.2, 145.1, 144.9, 144.4, 144.3, 144.2, 143.9, 142.7, 142.5, 142.4, 142.2, 142.1, 142.0, 141.9, 141.8, 141.75, 141.70, 141.6, 141.5, 141.4, 141.2, 139.9, 139.5, 139.2, 139.1, 136.6, 136.1, 135.6, 132.9, 132.8, 126.6, 122.5, 121.8, 121.3, 120.4, 120.3, 119.9, 119.6, 119.3, 119.1, 82.7, 69.8, 68.8, 40.6, 39.8, 39.2, 34.3, 31.4, 29.2, 27.8, 22.6, 22.4, 14.0. HRMS (MALDI, pos. mode, DCTB): calcd for [M-H]^+^ C_141_H_80_N_3_O_11_: 1990.579287; found: 1090.57874. Elemental analysis for C_141_H_81_N_3_O_11_ (1993.17) calcd. C 84.97 H 4.10 N 2.11; found C 82.05, H 4.18, N 2.07.

*Compound***11**:

To a solution of compound **5** (50 mg, 0.185 mmol) in anhydrous toluene (30 mL) were added C_60_ (220 mg, 0.3 mmol) and sarcosine (60 mg, 0.67 mmol). The reaction mixture was refluxed under nitrogen for 24 h. After the solution was cooled to room temperature, the solvent was removed under reduced pressure and the residue was purified by column chromatography on silica gel. A first column was used to remove the excess C_60_ with CS_2_, followed by a second one using CH_2_Cl_2_. The dyad was isolated as a brown powder (30 mg, 60%). ^1^H NMR (CDCl_3_/CS_2_, 300 MHz, 298 K): 8.00 (br m, 1H), 7.66 (br m, 1H), 7.59 (m, 1H), 7.34 (m, 5H), 5.11 (s, 2H), 5.01 (d, *J* = 9.3 Hz, 1H), 4.95 (s, 1H), 4.30 (d, *J* = 9.2 Hz, 1H), 3.90 (s, 3H), 2.80 (s, 3H). MS (MALDI-TOF, pos. mode, dithranol): *m*/*z*: calcd for: 1017.14; found: 1017.2 [M]^+^.

*Dyad***12**:

To a solution of compound **10** (10 mg, 5.10^−6^ mol) in anhydrous CH_2_Cl_2_ (2 mL) were added successively freshly distilled Et_3_N (7 μL, 5.10^−5^ mol), DMAP (6 mg, 5.10^−5^ mol), then methacryloyl chloride (5 μL, 5.10^−5^ mol). The reaction mixture was stirred at room temperature under argon for 48 h. The residue was purified by silica gel column chromatography using CH_2_Cl_2_ as the eluent. Dyad **12** was isolated as a purple powder (9.6 mg, 93%).

**Figure 6:**
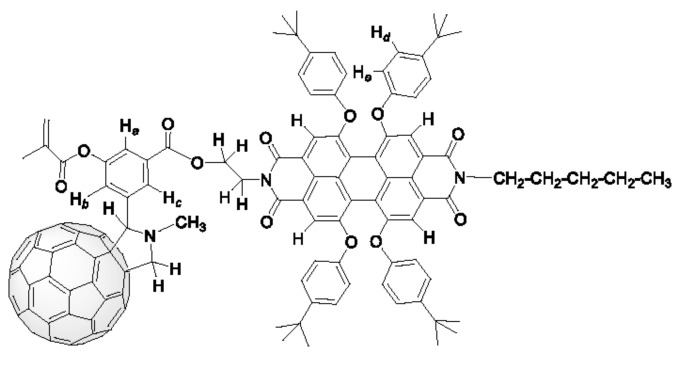
Formula of dyad **12** for ^1^H NMR spectrum analysis.

^1^H NMR (CDCl_3_, 300 MHz, 298 K) *δ*: 8.27 (br s, 2H, H perylene), 8.19 (s, 2H, H perylene), 7.72 (s, 1H, Ha or Hb or Hc), 7.20 and 7.19 (2m, 8H, H_D_ or H_E_), 6.80 and 6.75 (2d, ^3^*J* = 8.5 Hz, 8H, H_D_ or H_E_), 6.30 (s, 1H, *=*CH_2_*)*, 5.71 s, 1H, *=*CH_2_*)*, 4.91 (d, *J* = 9.5 Hz, 1H, -NCH_2_CH_2_O-), 4.84 (s, 1H, CH pyrrolidine), 4.75 (br s, 1H, -NCH_2_CH_2_O-), 4.63 (br s, 2H, CH_2_ pyrrolidine), 4.40 (br s, 1H, -NCH_2_CH_2_O-), 4.18 (br s, 1H, -NCH_2_CH_2_O-), 4.12 (t, *J* = 7.5 Hz, 2H, NCH_2_(CH_2_)_3_CH_3_), 2,71 (s, 3H, N-CH_3_), 2.01 (s, 3H, CH_3_-C=), 1,67 (m, 2H, -CH_2_), 1,37 (m, 4H, -CH_2_CH_2_-), 1,28 (s, 36H, *t*Bu), 0,90 (t, *J* = 7 Hz, 3H, CH_3_) (Figure 6).

In agreement with observations for dyad **10**, two protons of the aromatic platform (probably Hb and Hc) were not characterized on the spectrum at 298 K.

^13^C NMR (CDCl_3_, 75 MHz, 298 K) *δ*: 165.5, 163.6, 163.5, 156.3, 155.8, 153.3, 153.0, 152.3, 147.4, 147.2, 146.4, 146.2, 146.1, 146.0, 145.9, 145.7, 145.4, 144.6, 144.4, 142.7, 142.6, 142.2, 142.0, 140.2, 139.5, 139.3, 137.0, 135.8, 135.7, 133.2, 133.1, 127.8, 126.8, 122.7, 122.1, 121.6, 120.8, 120.5, 120.0, 119.6, 119.2, 82.6, 70.0, 69.0, 40.8, 40.0, 34.5, 31.6, 29.8, 29.4, 28.0, 22.6, 18.5, 14.2. HRMS (MALDI, pos. mode, DCTB): calcd for [M-H]^+^ C_145_H_84_N_3_O_12_: 2058.604495; found: 2058.6027.

*C_60_-PDI Polymer **Pol 3**:*

To a solution of dyad **10** (100 mg) in distilled dioxane (3.5 mL) under argon atmosphere, were added successively Et_3_N (10 µL) and poly(methacryloyl chloride) 25% solution in dioxane (Polyscience) (40 µL). The reaction was stirred at room temperature for 24 h before being refluxed for 2 h. The mixture was concentrated under reduced pressure. The product was purified by successive precipitation using CHCl_3_/CH_3_OH and CHCl_3_/hexane.

## Figures and Tables

**Table 1:** Oxidation and reduction potentials of dyad **9**, references PDI **8**, and fullerene **11**. (V vs. Fc^+^/Fc). Scan rate: 100 mV/s.

Compound	E^0^_red3_	E^0^_red2_	E^0^_red1_	E^0^_ox1_
PDI **8**	-	−1.40	−1.24	+0.77
Dyad **9**	−1.55	−1.38	−1.22 *	+0.81 *
FP **11**	−1.78	−1.55	−1.18	+0.98 **

* Two-electrons reduction or oxidation process; ** irreversible process.

**Table 2:** Absorption spectra in CH_2_Cl_2_ and toluene for PDI **7** and dyad **9** (C = 10^−5^ M): λ_max_ is underlined. Emission spectra in CH_2_Cl_2_ and toluene for PDI **7** and dyad **9** (λ_exc_ = 520 nm; C = 5 × 10^−6^ M): quantum yields were calculated using cresyl violet perchlorate as reference (φ_F_ = 0.54 in ethanol).

Compound	λ_abs_ (nm) (CH_2_Cl_2_)	λ_abs_ (nm) (toluene)	λ_em_ (nm) (CH_2_Cl_2_)	λ_em_ (nm) (toluene)	φ_F_ (CH_2_Cl_2_)	φ_F_ (Toluene)
PDI **7**	452, 540, 580	448, 535, 575	607	601	~1	~1
Dyad **9**	229, 452, 543, 579	283, 451, 535, 573	610	603	2.8 × 10^−3^	1.1 × 10^−2^

## Data Availability

Not applicable.

## Supplementary Materials

The following supporting information can be downloaded at: https://www.mdpi.com/article/10.3390/molecules27196522/s1, Figures S1–S22: 1H and 13C NMR spectra and mass spectra of compounds [114], Figure S23: cyclic voltammograms, Figures S24–S26: Absorption and fluorescence spectra [115], Figures S27 and S28: DSC-TGA spectra and absorption spectra of polymers; Figures S30–S33: 1H and 13C NMR spectra of polymers.
