# Supplementary material for: Fullerene-Perylenediimide (C60-PDI) Based Systems: An Overview and Synthesis of a Versatile Platform for Their Anchor Engineering
Source: Molecules. 2022 Oct 2;27(19):6522. doi: 10.3390/molecules27196522 (PMC9571100; doi:10.3390/molecules27196522)

SUPPORTING INFORMATION for

Fullerene-Perylenediimide (C_{60}-PDI) Systems: An Overview and Synthesis of a Versatile Platform for their Anchor Engineering

Aurel Diacon,[1,2] Oksana Krupka,[1,3] and Piétrick Hudhomme [1]*

[1] Univ Angers, CNRS, MOLTECH-Anjou, SFR MATRIX, Angers F-49000, France.

[2] Department of Bioresources and Polymer Science, Faculty of Chemical Engineering and Biotechnologies, University Politehnica of Bucharest, 1-7 Gh. Polizu Street, 011061 Bucharest, Romania

[3] Department of Chemistry, Taras Shevchenko National University of Kyiv, 60 Volodymyrska, 01033 Kyiv, Ukraine

* Correspondence: pietrick.hudhomme@univ-angers.fr; Tel.: +33-2-4173-5094

List of contents of the Supporting Information:

**Experimental methods**	
**^{1}H and ^{13}C NMR spectra of 3-(benzyloxy)-5-formylbenzoic acid 6**	**S1-S2**
**^{1}H, ^{13}C NMR spectra of compound 7**	**S3-S4**
**^{1}H, ^{13}C NMR spectra of compound 8**	**S5-S6**
**^{1}H, ^{13}C NMR spectra of dyad 9**	**S7-S9**
**^{1}H, ^{13}C NMR spectra of dyad 10**	**S10-S17**
**Mass spectrum of dyad 10**	**S18**
**^{1}H, ^{13}C NMR spectra of compound 11**	**S19**
**^{1}H, ^{13}C NMR spectra of dyad 12**	**S20-S21**
**Mass spectrum of dyad 12**	**S22**
**Electrochemical characterizations**	**S23**
**Photophysical characterizations**	**S24-S26**
**DSC-TGA analysis of polymers**	**S27-S28**
**UV-Vis spectra of polymers**	**S29**
**^{1}H, ^{13}C NMR spectra of Pol 3**	**S30-S31**
**^{1}H, ^{13}C NMR spectra of Pol 2**	**S32-S33**

Experimental methods:

The following chemicals were obtained commercially and were used without any purification.
For syntheses, THF, dioxane and toluene were distilled over sodium / benzophenone.
Poly(methacryloyl chloride) 25% solution in dioxane was purchased from Polyscience.
Thin-layer chromatography (TLC) was performed on aluminum sheets coated with silica gel 60 F_{254}. Column chromatography was carried out on silica gel 60A (40-60 μm). ^{1}H (300 MHz), ^{13}C (75 MHz) NMR spectra were recorded on a spectrometer Bruker Avance III 300. ^{1}H (500 MHz), ^{13}C (125 MHz) NMR spectra were recorded on spectrometer Bruker Avance DRX 500. Chemical shifts are reported as δ values in ppm using residual $CHCl_3$ or acetone as the reference. IR spectra were performed on a Bruker Vertex 70 spectrophotometer. Matrix-assisted laser-desorption/ionization mass spectrometry was performed on a Bruker Daltonics BIFLEX III spectrometer by using dithranol as the matrix. High resolution mass spectra (HRMS) were recorded with a LTQ Orbitrap (Thermo Scientific) under electrospray ionization (ESI) in positive ionization mode or with a JEOL JMS S3000, MALDI ionisation and SPIRAL TOF detector using DCTB as the matrix. Elemental analysis were carried out on a Thermo Scientific Flash 2000. Electronic absorption spectra were recorded with a Lambda 19 NIR model from Perkin-Elmer. The UV-Vis spectra were recorded using a Jasco V-500 spectrophotomether. The fluorescence spectra were registered using a Jasco FP-6500 Able Jasco spectrofluorimeter. The thermogravimetric analysis (TGA) was performed using a Thermal Analysis Q500 instrument, considering the next parameters: nitrogen flow; samples mass: ~4 mg; temperature range: room temperature -800°C; heating rate: 10°C/min. Differential scanning calorimetry (DSC) was used to analyze the glass transition temperature (T_g) of the different polymers. The measurements were performed at a 10°C/min heating rate on a DSC Q20 TA instruments equipment, under nitrogen flow. The tests were carried out on samples having around 8 mg, in at least two heating/cooling cycles, between room temperature and 160°C.

Figure S1: ^{1}H NMR spectrum (300 MHz, CDCl3) of 3-(benzyloxy)-5-formylbenzoic acid 6:

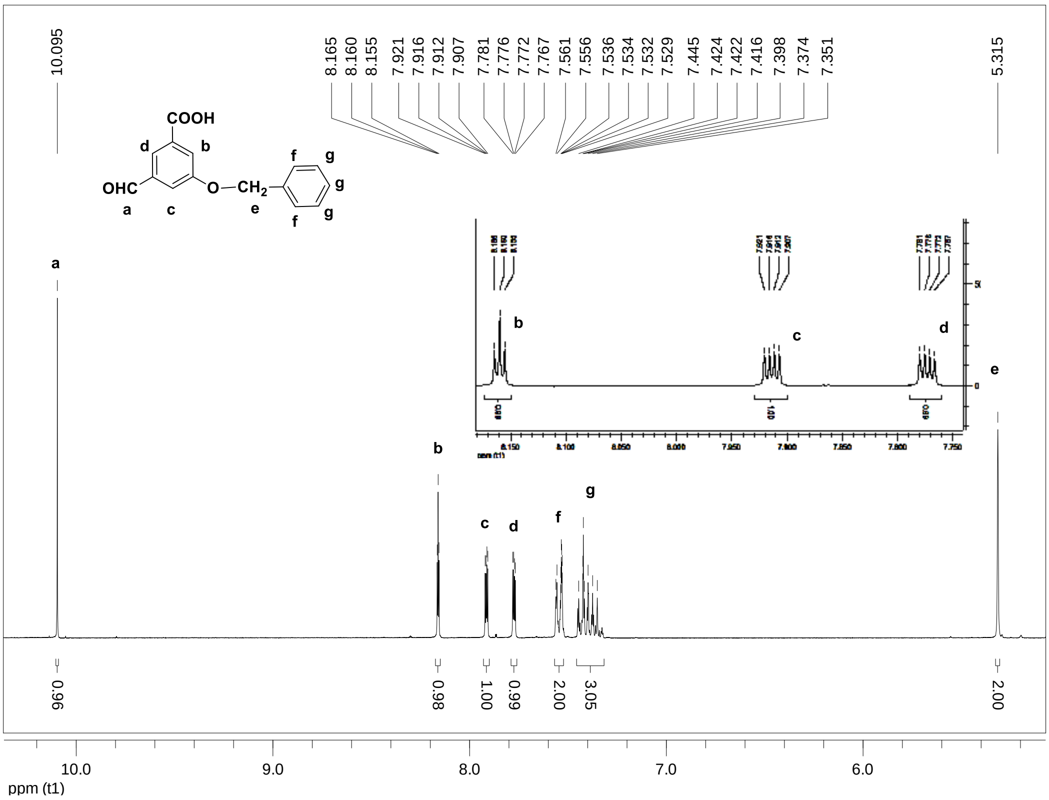

Figure S2: ^{13}C NMR spectrum (75 MHz, CDCl3) of 3-(benzyloxy)-5-formylbenzoic acid 6:

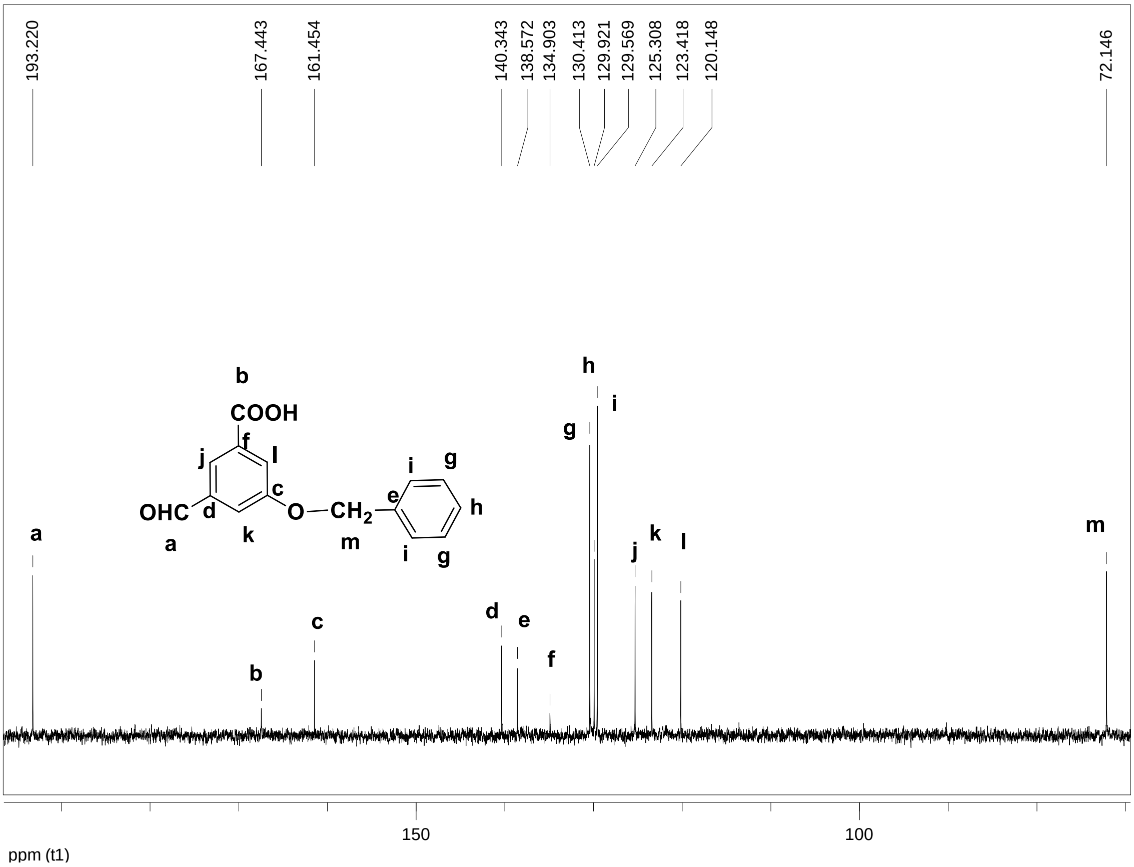

Figure S3: ^{1}H NMR spectrum (300 MHz, $CDCl_3$) of compound 7:

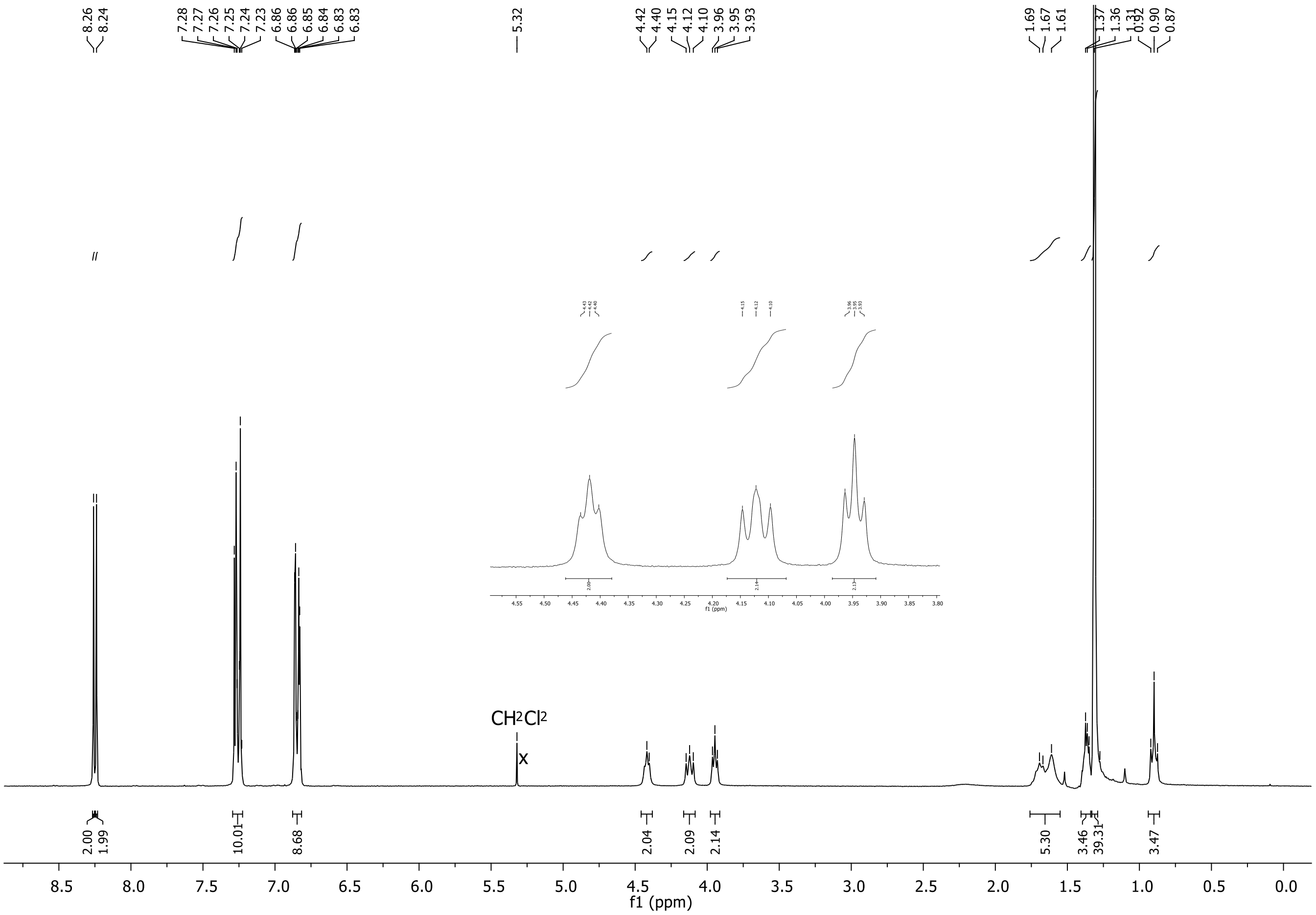

Figure S4: ^{13}C NMR spectrum (75 MHz, $CDCl_3$) of compound 7:

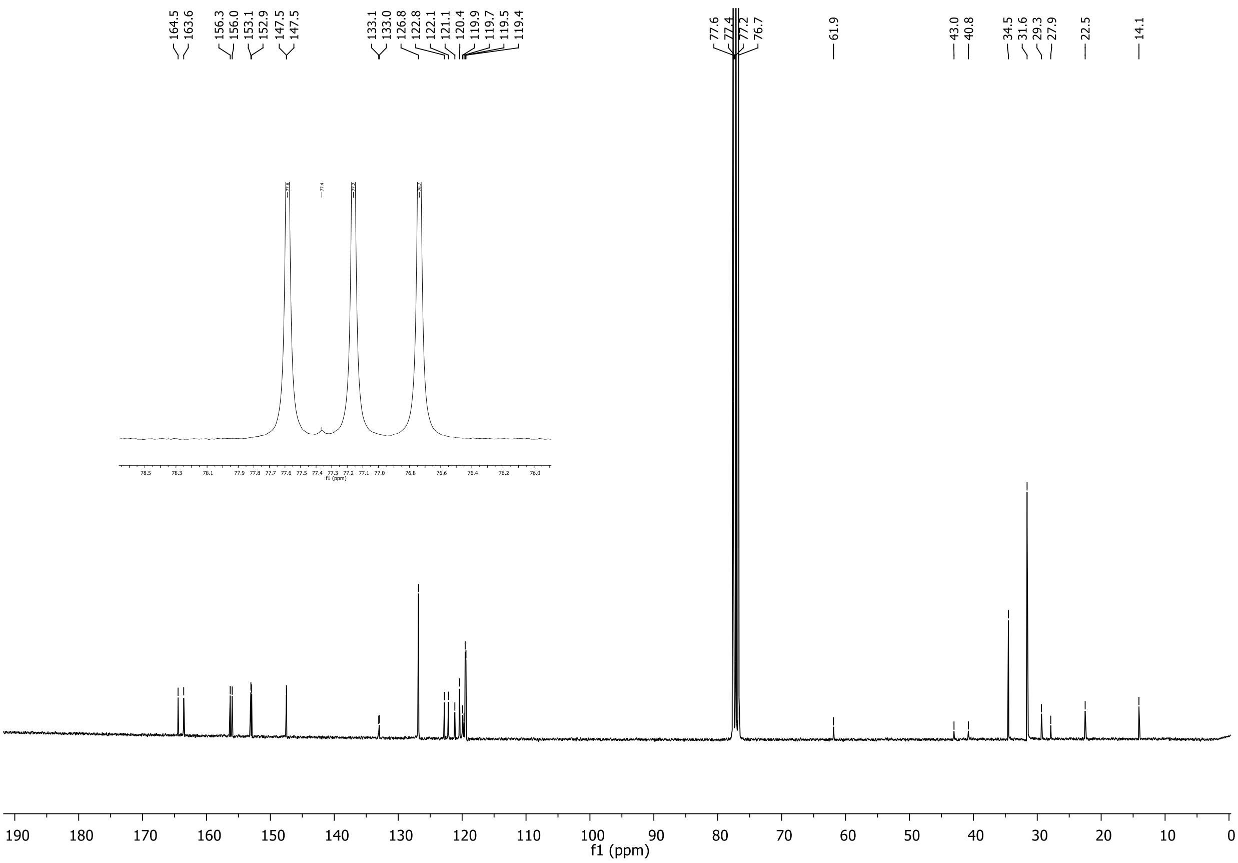

Figure S5: ¹H NMR spectrum (500 MHz, $CDCl_3$) of compound 8:

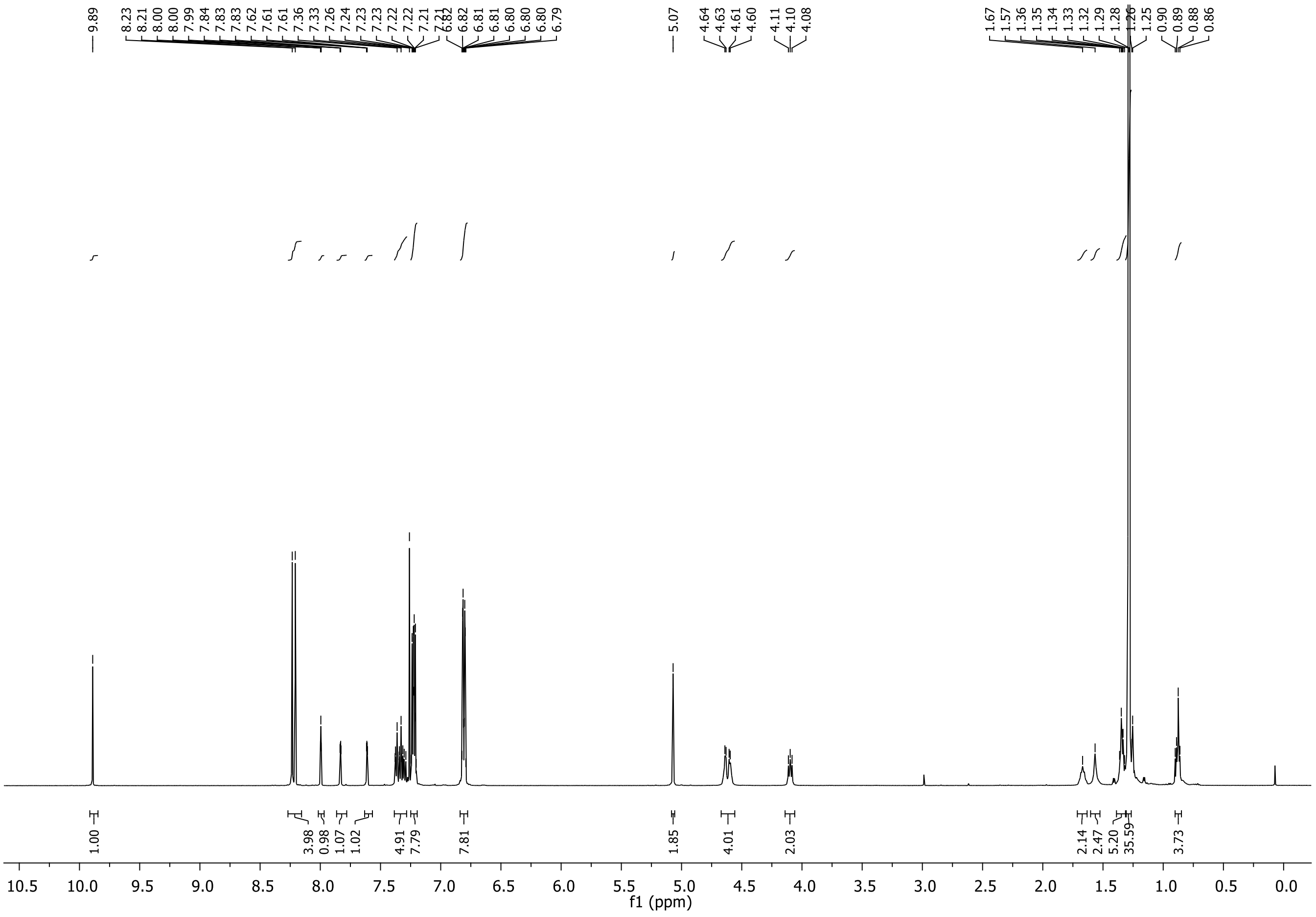

Figure S6: ¹³C NMR spectrum (125 MHz, $CDCl_3$) of compound 8:

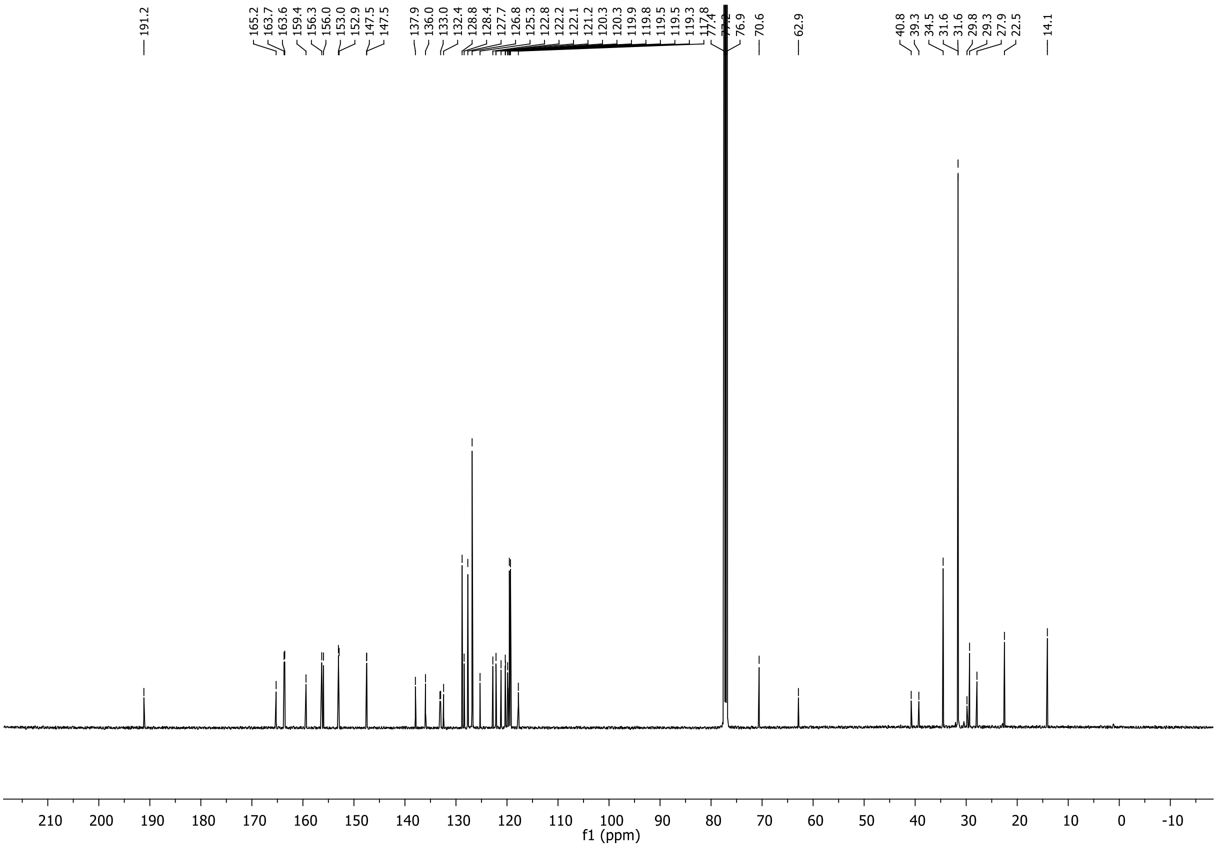

Figure S7: ^{1}H NMR of dyad 9 (500 MHz, $CDCl_3$, 298K) :

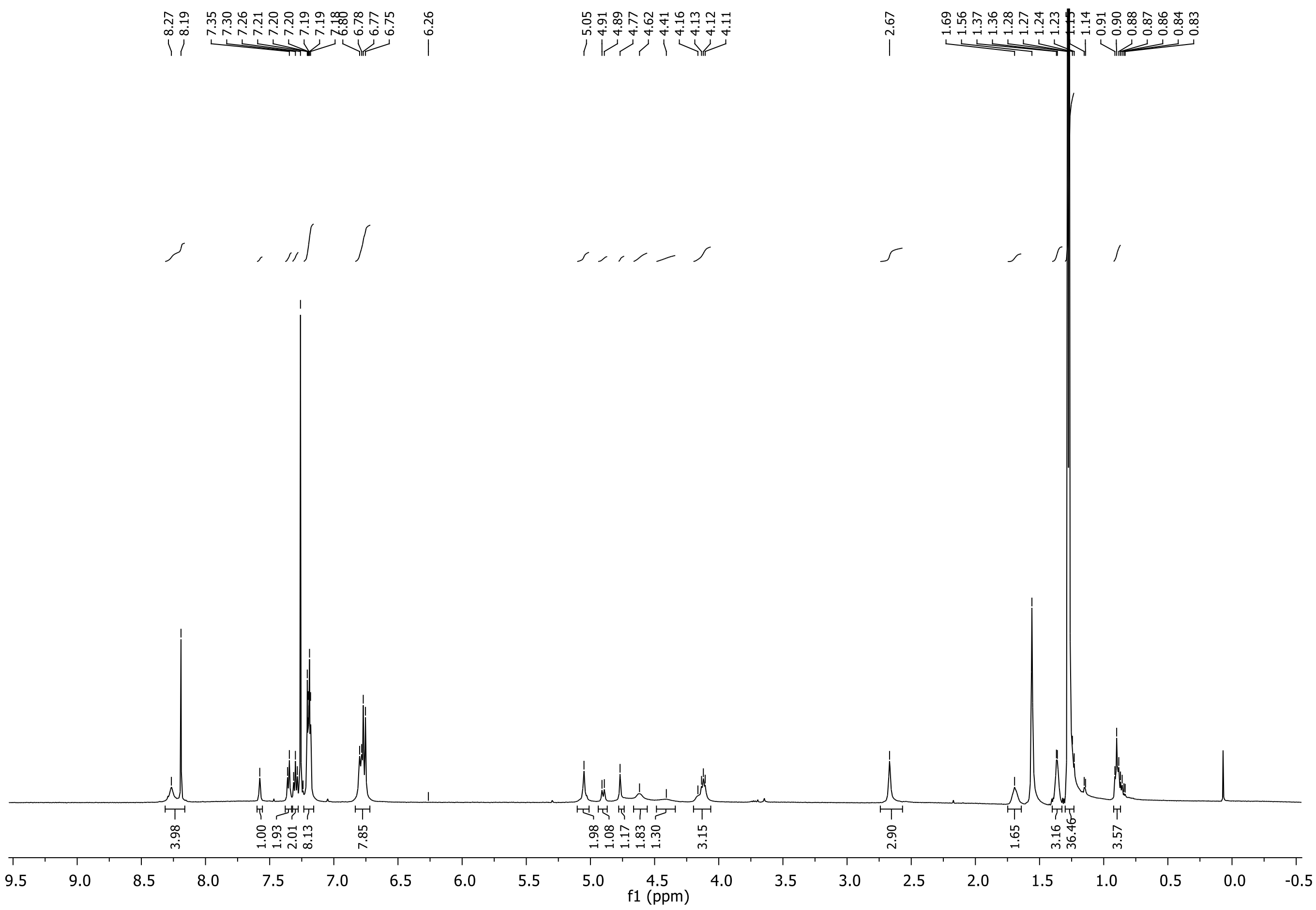

Figure S8: ^{1}H NMR of dyad 9 (500 MHz, $CDCl_3$) : extension of the spectrum corresponding to peaks of the PDI core at 298 K (top), 308 K (middle), 328 K (down) :

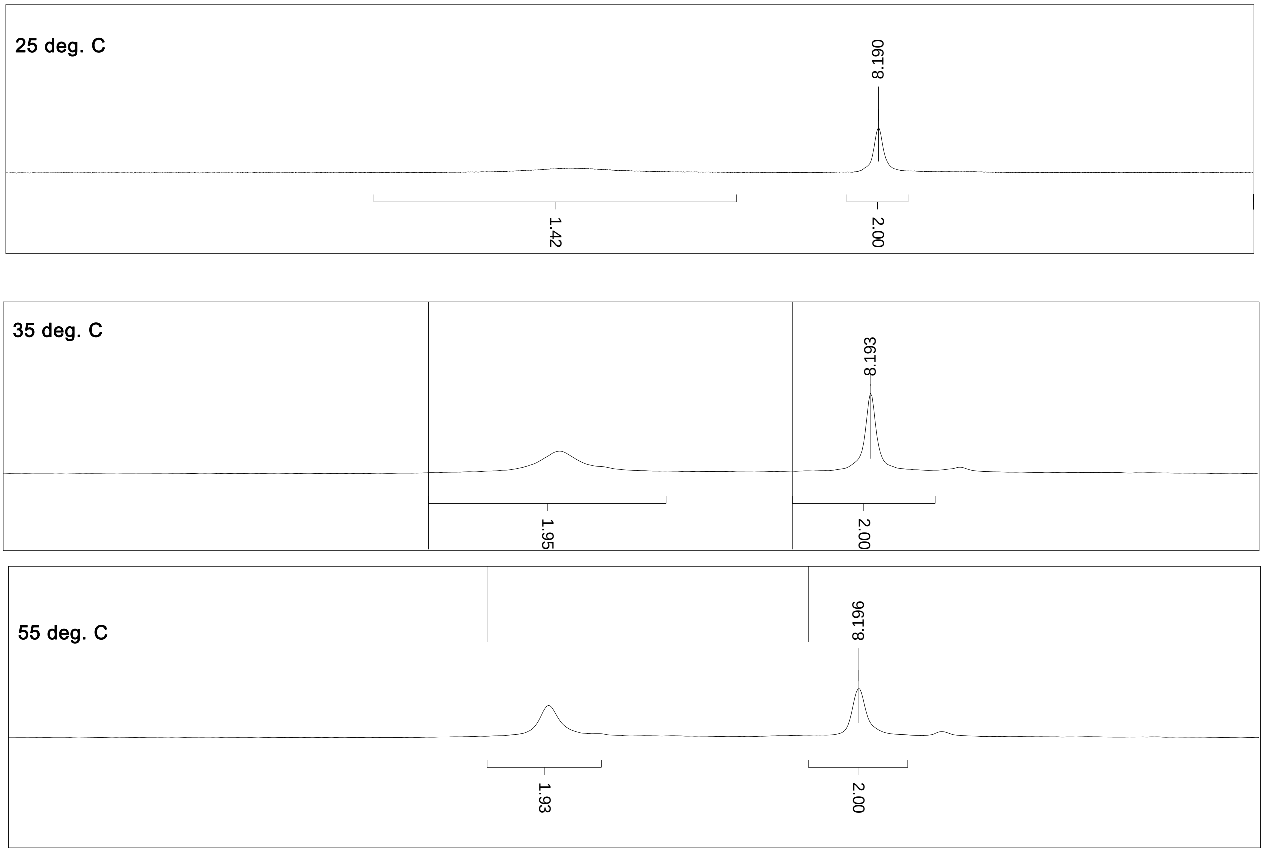

Figure S9: ^{13}C NMR spectrum (125 MHz, $CDCl_3$) of dyad 9:

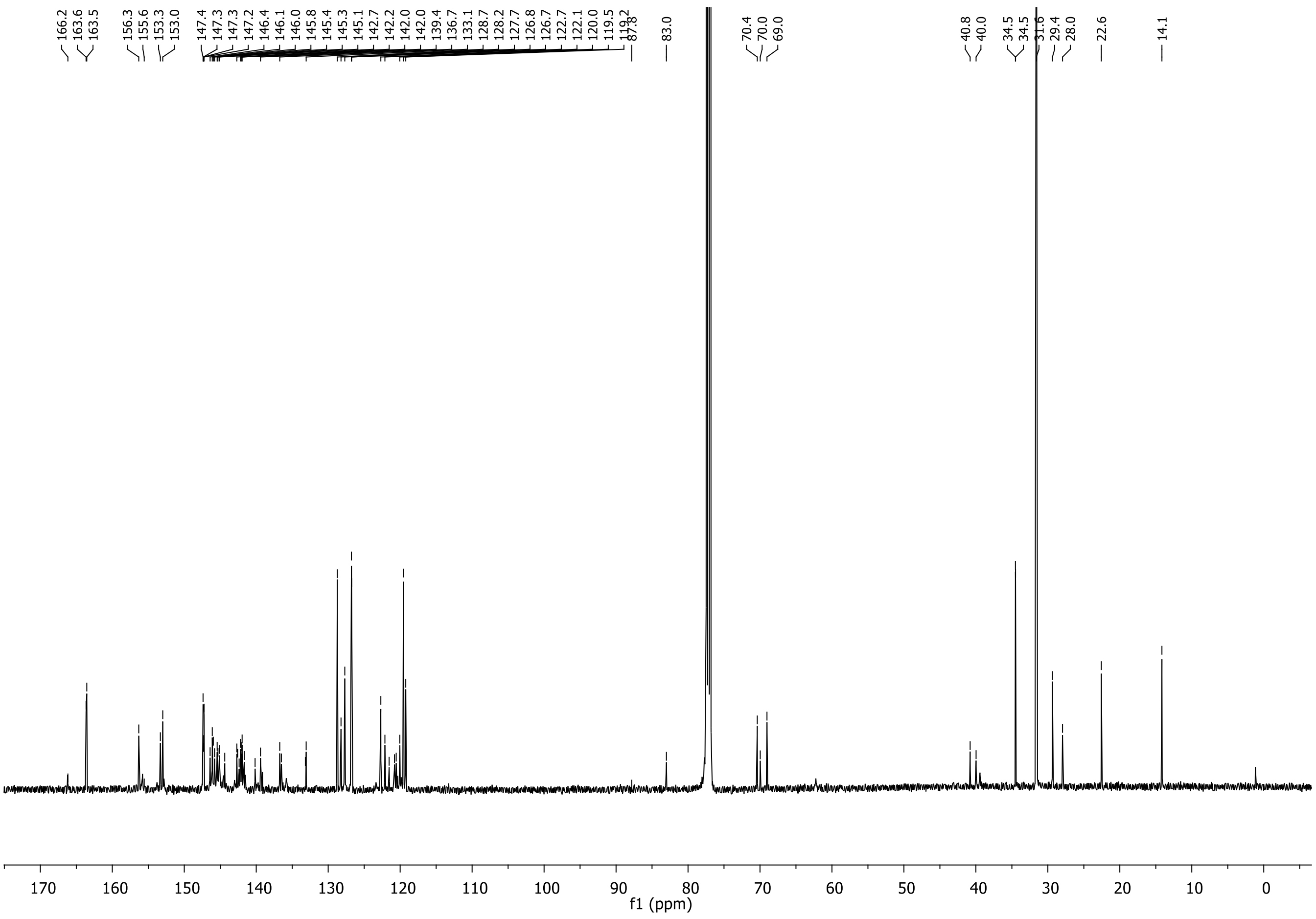

Figure S10: ^{1}H NMR of dyad 10 (300 MHz, $CDCl_3$) :

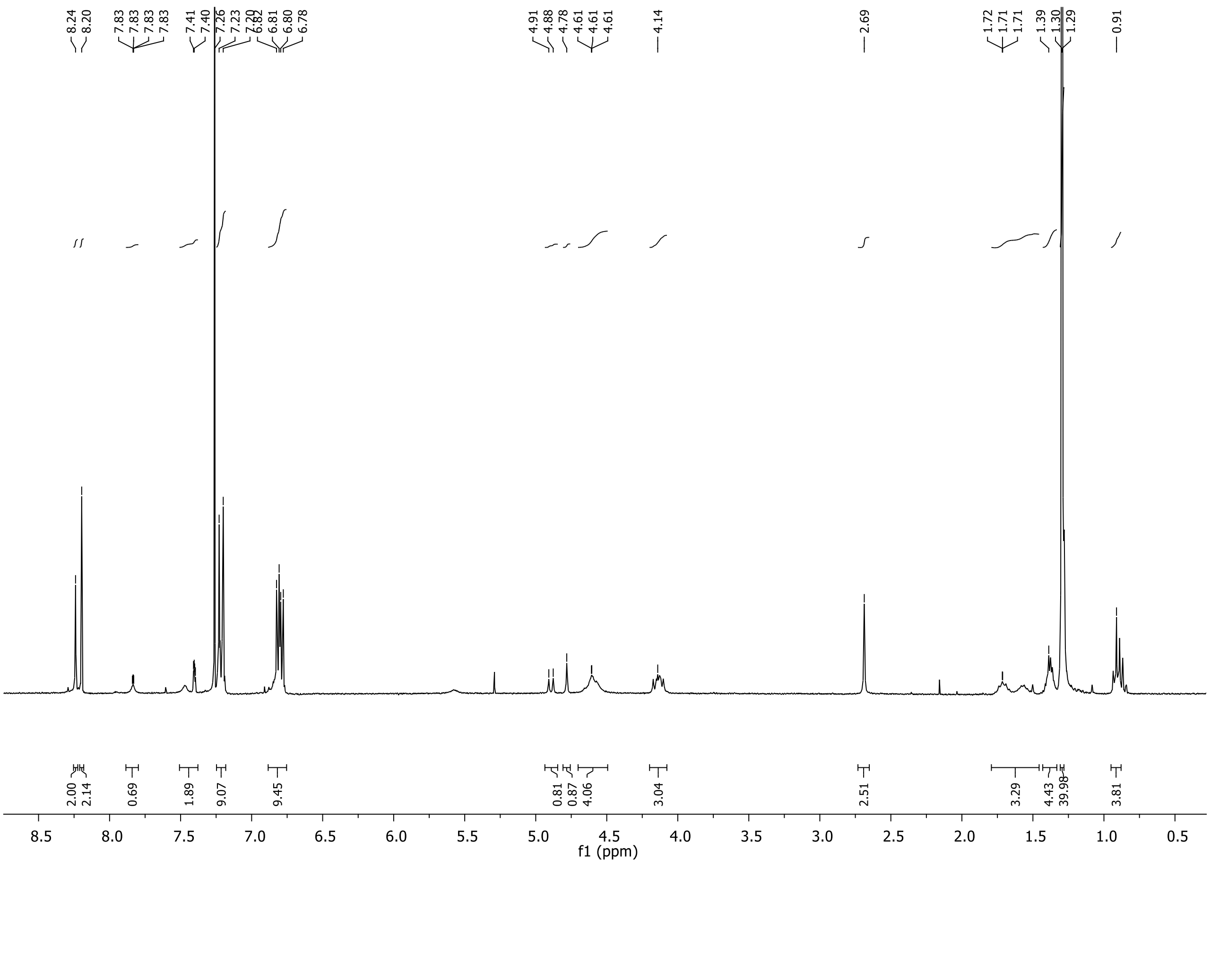

Figure S11: ^{1}H NMR of dyad 10 (500 MHz, CDCl$_3$, 328K):

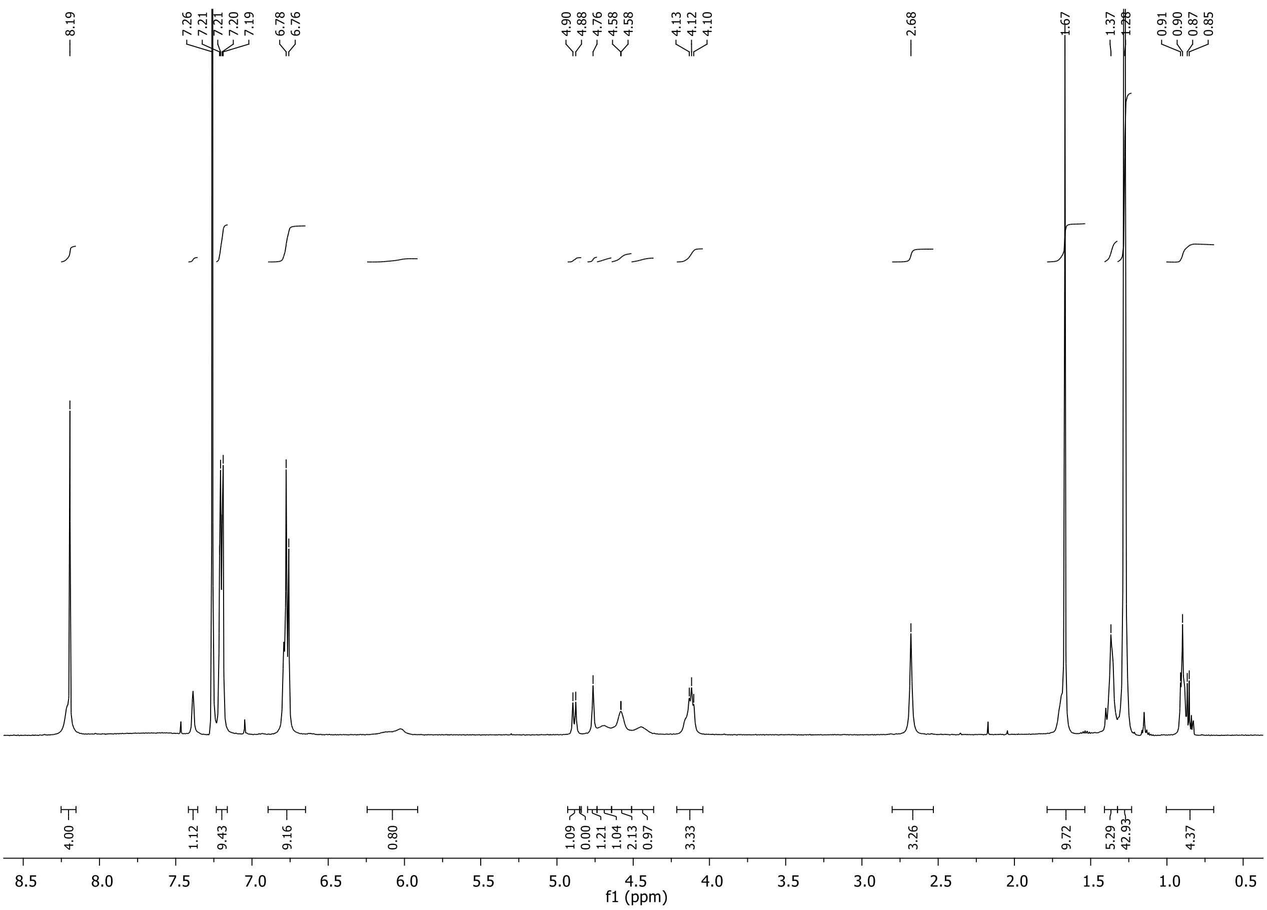

Figure S12: ^{1}H NMR of dyad 10 (500 MHz, CDCl$_3$, 298K): Temperature dependent NMR studies of dyad 10 : Protons of the perylene core and of the aromatic platform

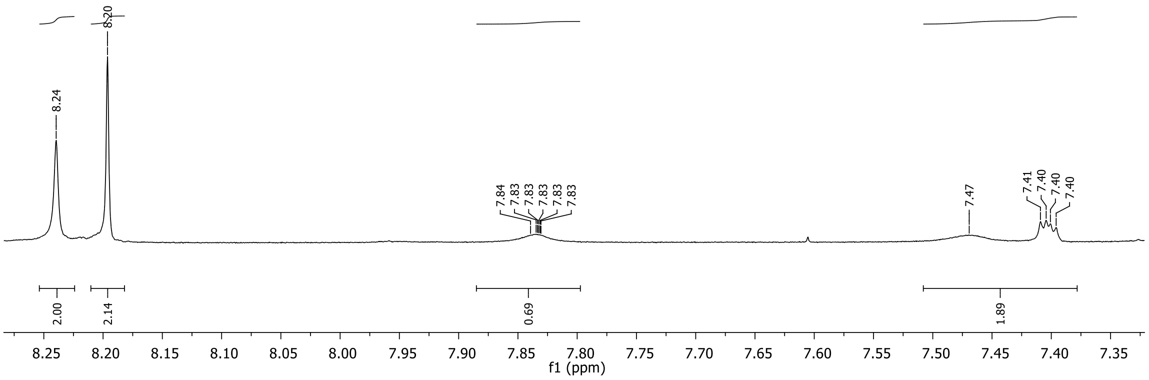

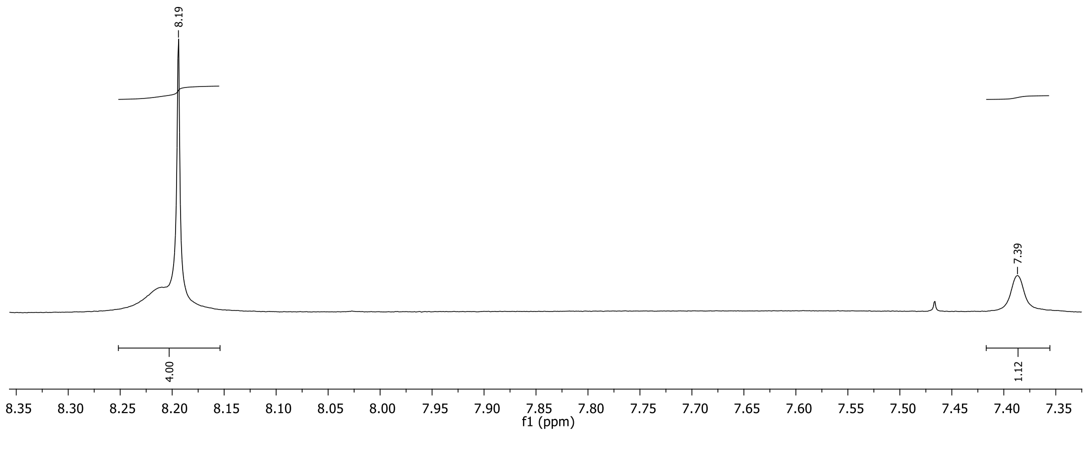

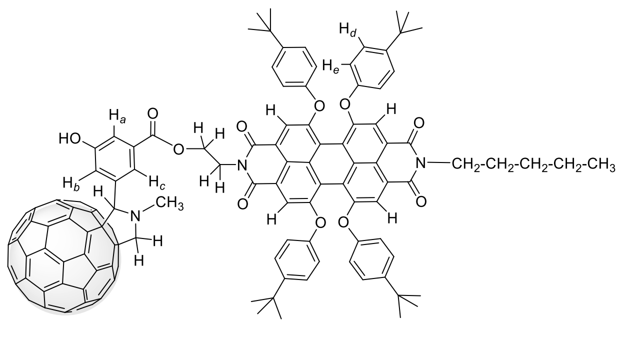

The ^{1}H NMR spectrum at 298 K showed for protons of the perylene core a well-defined singlet for two protons and a broad singlet for the two others. This suggested a problem of relaxation which was demonstrated by the obtention in the spectrum recorded at 328 K of the two expected singlets at 8.20 and 8.24 ppm.

Concerning the three protons of the aromatic platform, noted H_A, H_B and Hc, only one of these protons at 7.38 ppm is observed in the spectrum at 298 K. This problem of relaxation due to the presence of both fullerene and PDI substituents around this aromatic platform is partially solved at 328 K. In the latter case, a well defined doublet of doublet at 7.40 ppm (^3J = 1,5 and 2,4 Hz) is observed accompanied with two broad singlets at 7.47 and 7.83 ppm. For the attribution of these Ha, Hb and Hc protons of the aromatic platform, it should be reasonable to consider that both protons exhibiting higher problems of relaxation correspond to H_B and H_C protons which are closer to bulky C_{60} fullerene. Consequently, H_A could be attributed to the proton at 7.40 ppm. The other possibility considers the table of increments for substituted benzenes[114], following the formula: δ = 7.27 + ortho + meta + para

Nevertheless, this technique suffers from the lack of information in the table of increments about the influence of the pyrrolidine ring on the chemical shift. Using this table, the proton H_B should be the more shielded and the proton H_C the more deshielded.

For the attribution of H_D and H_E protons, calculated chemical shifts are in good agreement with those observed on the ^{1}H NMR spectrum. Consequently, protons H_D can be attributed to the two doublets at 7.20 ppm and protons H_E to the two doublets at 6.78 ppm.

Figure S13: ^{1}H NMR (300 MHz, $CDCl_3$, 328K (up) and 500 MHz, $CDCl_3$, 298K (down)
Temperature dependent NMR studies of dyad 10: Protons of the linkers –OCH_2CH_2N- and pyrrolidine ring :

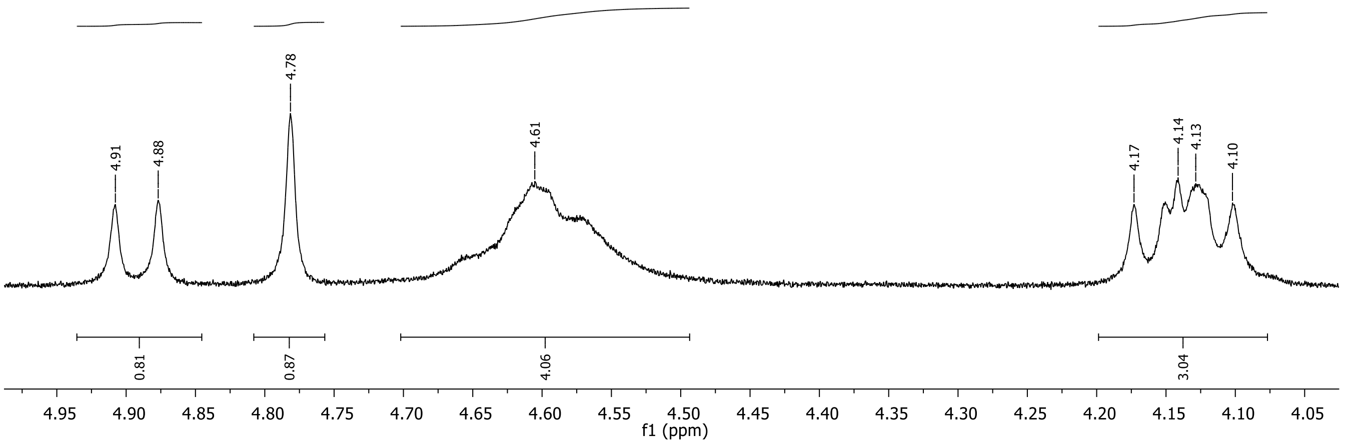

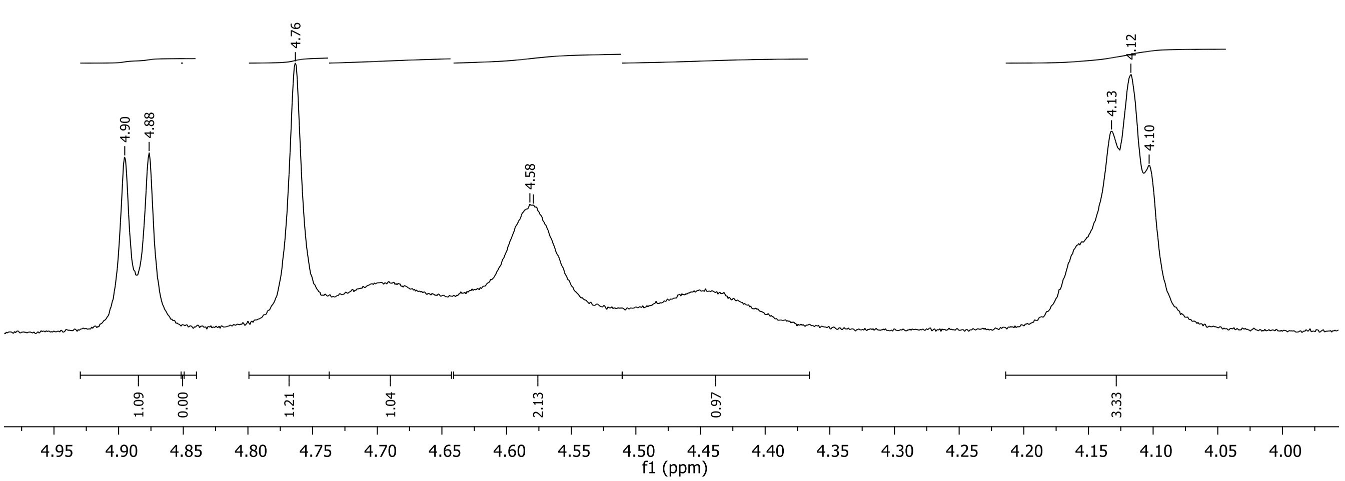

[114] Günther, H.; *NMR spectroscopy: an introduction*. Wiley Publishing: New York, United States,1980.

The ^{1}H NMR spectrum at 298 K presents signals in the range 4 – 5 ppm for which the better resolution allows an easier attribution :

4.88 (d, J = 9.5 Hz, 1H, $-NCH_2CH_2O-$ group)
4.76 (s, 1H, CH pyrrolidine)
4.69 (br s, 1H, $-NCH_2CH_2O-$ group)
4.58 (br s, 2H, CH_2 pyrrolidine)
4.45 (br s, 1H, $-NCH_2CH_2O-$ group)
4.16 (br s, 1H, $-NCH_2CH_2O-$ group)
4.10 (t, J = 7.5 Hz, 2H, $N\underline{CH_2}(CH_2)_3CH_3$)

Figure S14: COSY spectrum of dyad 10

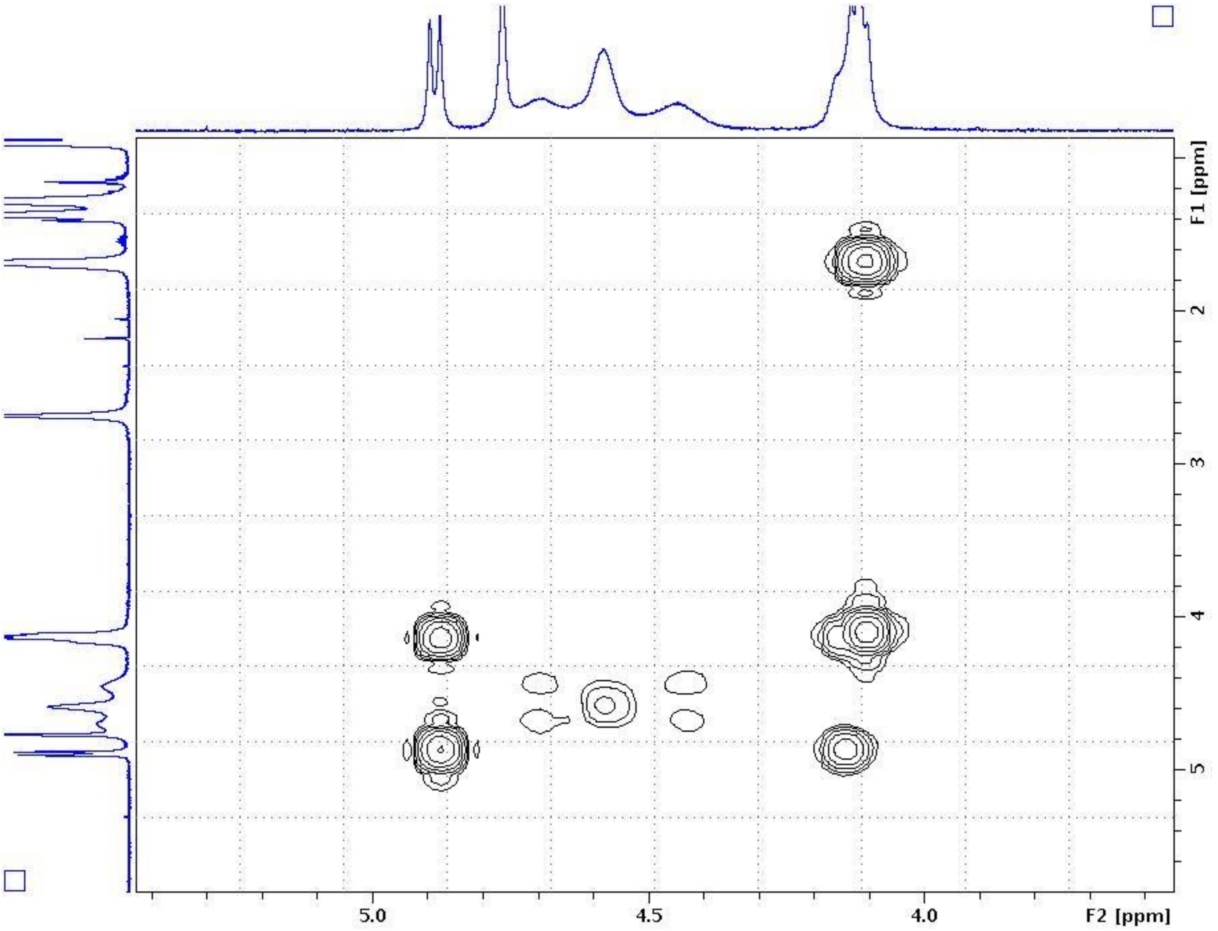

This attribution is conforted thanks to the COSY spectrum :

Protons at 4.10 ppm (t, J = 7.5 Hz, 2H) are correlated with the CH_2 groups at 1.7 ppm and can be attributed to the $–NCH_2$-group of the pentyl chain. Protons at 4.58 ppm (br s, 2H) present no correlation and can be attributed to the CH_2 group of the pyrrolidine ring.
Consequently, the doublet at 4.88 ppm and three broad singlets at 4.69, 4.45 and 4.16 ppm correspond to the $-NCH_2-CH_2O-$ group. Proton at 4.16 ppm (br s, 1H) is correlated with the proton appearing at 4.88 ppm (d, J = 9.5 Hz, 1H) whereas proton at 4.45 ppm (br s) is correlated to the proton at 4.69 ppm (br s). The coupling constant (J = 9.5 Hz) is in agreement with a ^{2}J coupling of a methylene group.

Figure S15: ^{13}C NMR spectrum (125 MHz, $CDCl_3$) of dyad 10 :

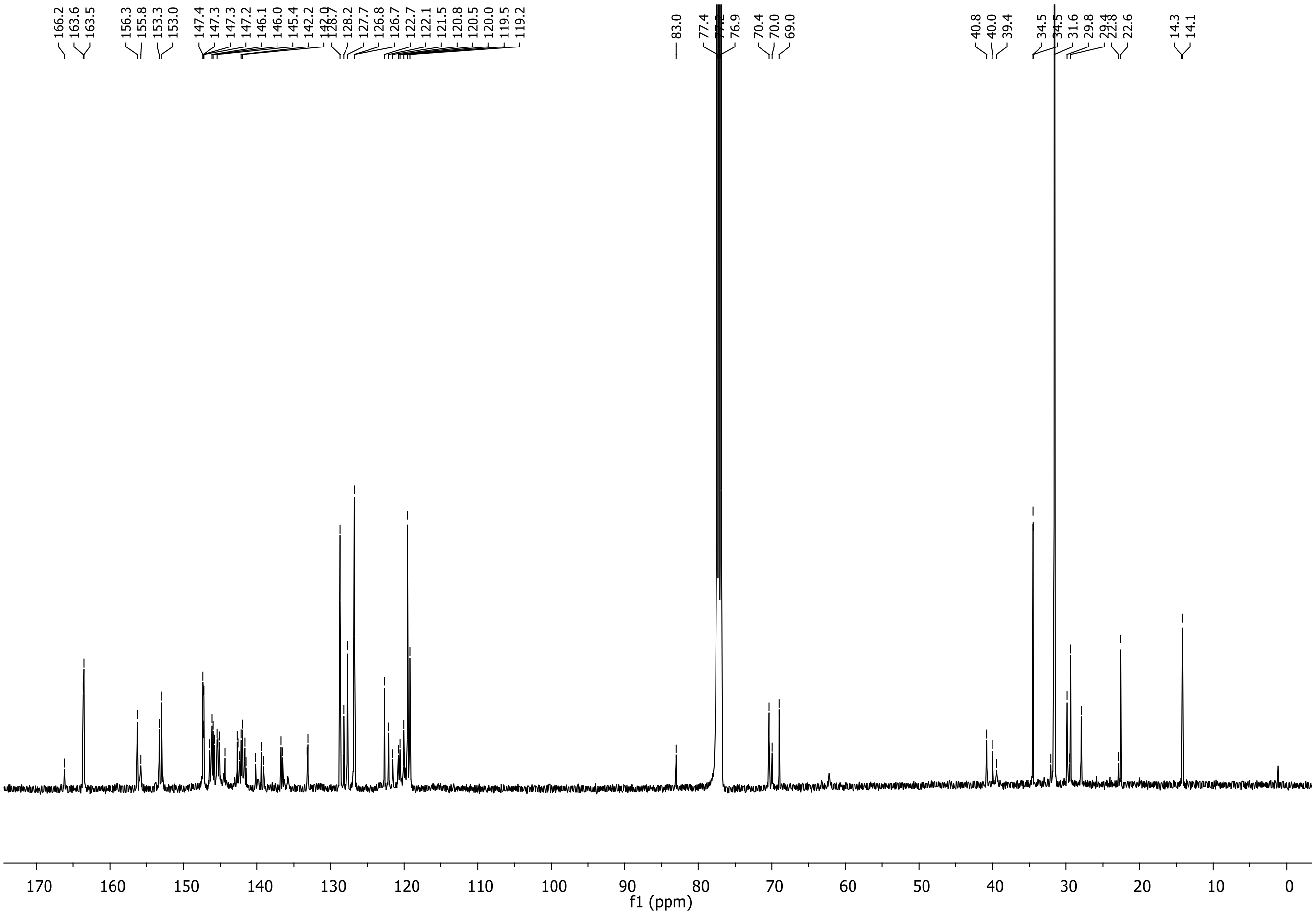

Figure S16: DEPT ^{13}C spectrum of dyad 10 :

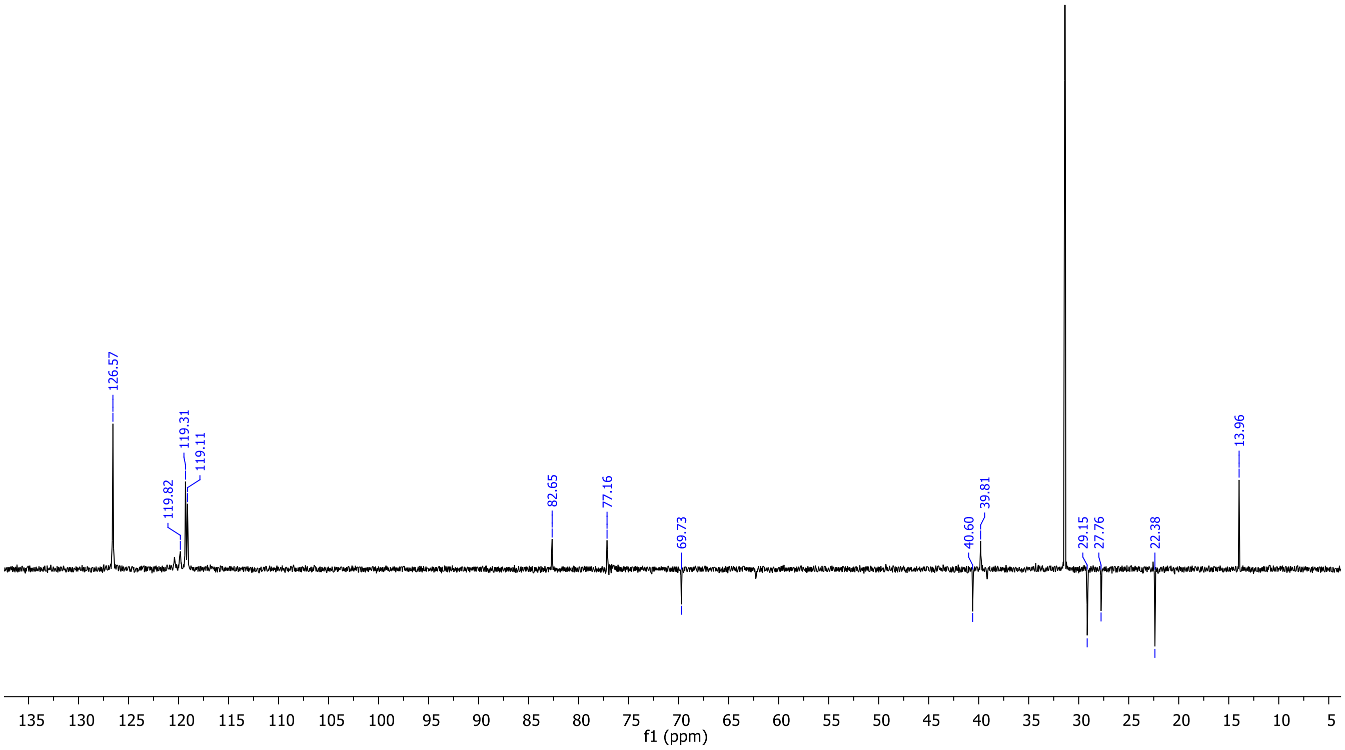

Figure S17: HMQC spectrum of dyad 10 :

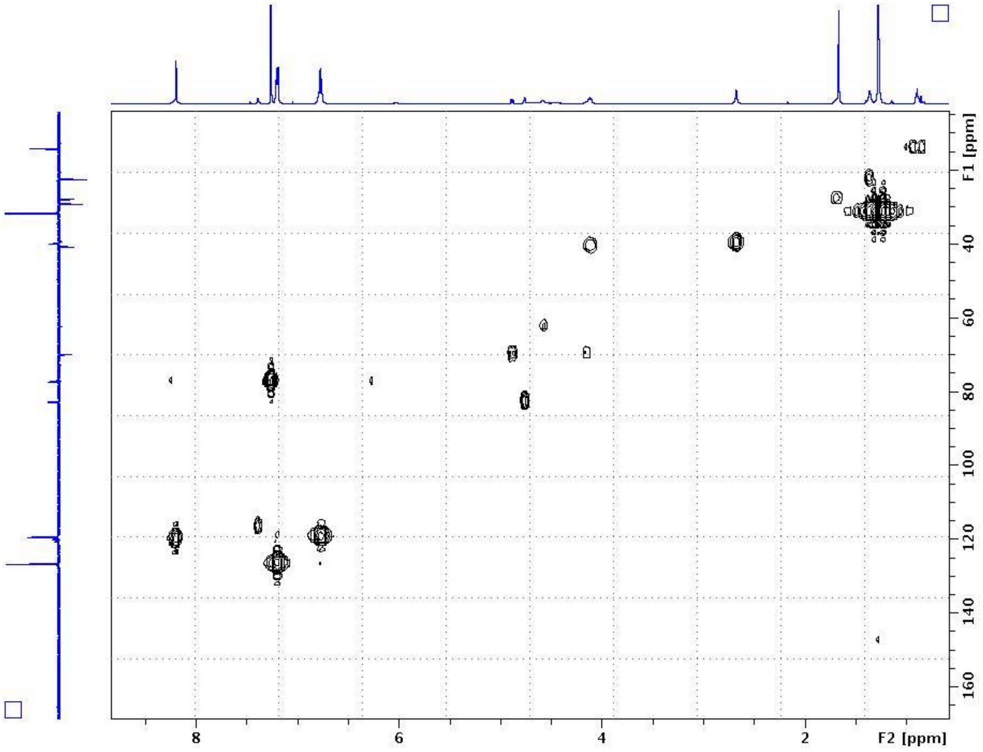

The proton signal at 2.68 ppm attributed to the N-CH_3 group of the pyrrolidine ring is correlated with the carbon at 39.8 ppm
The broad singlet at 4.58 ppm attributed to the CH_2 group of the pyrrolidine ring is correlated with the carbon at 40.6 ppm.

Figure S18: Experimental HRMS Spectrum of dyad 10 (MALDI, pos. mode, DCTB):

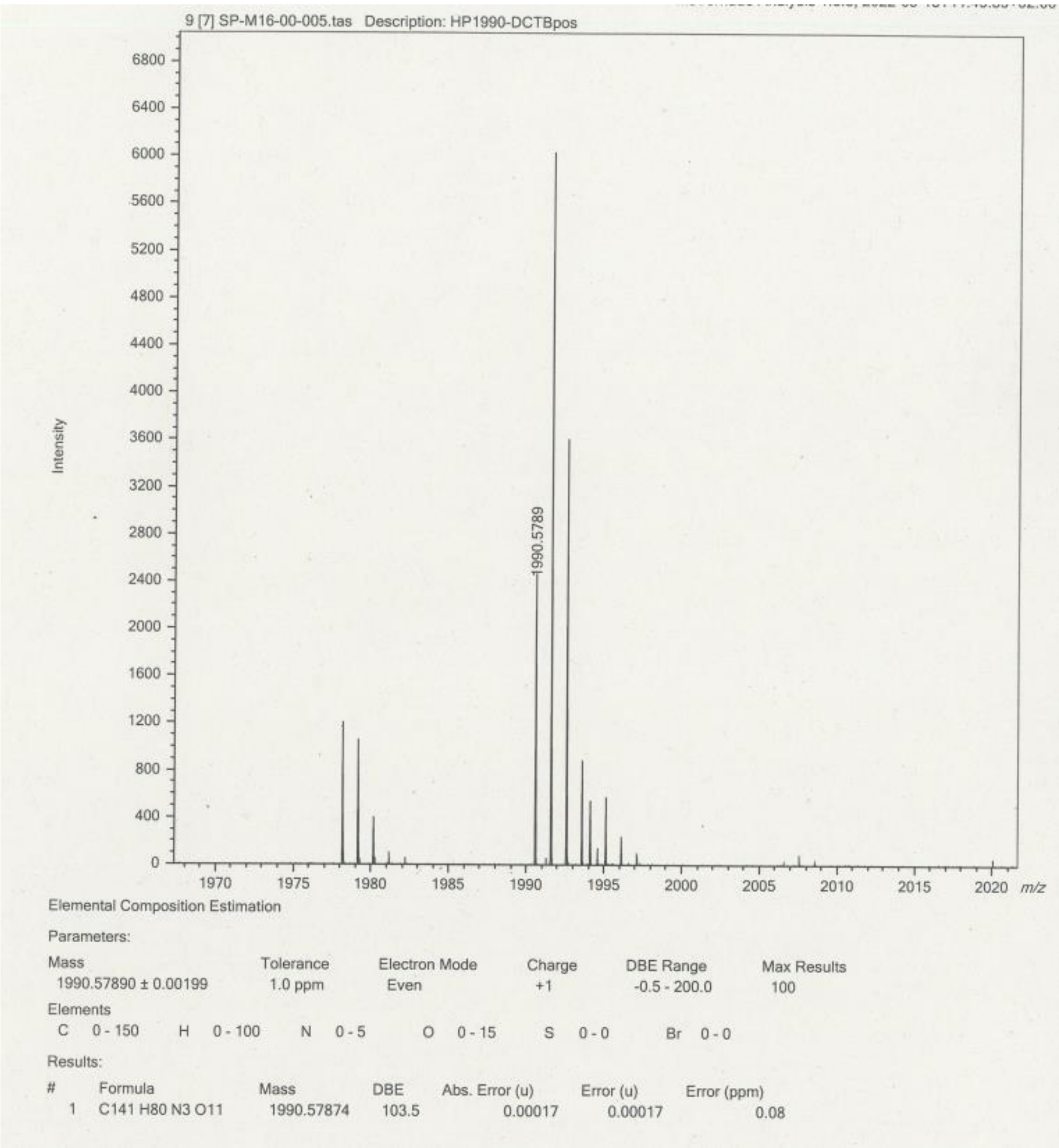

Theoretical spectrum for molecular peak [M-H]$^+$ for dyad 10:

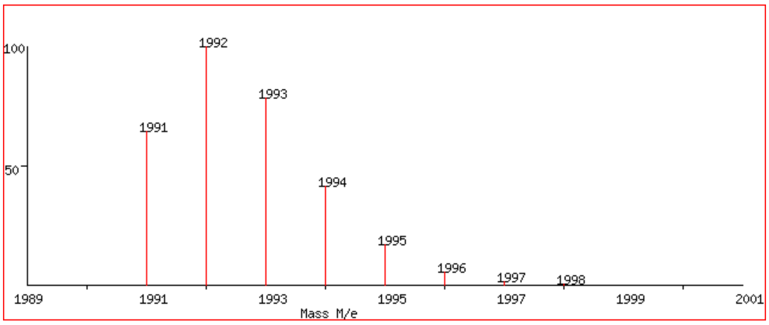

Figure S19: ^{1}H NMR of compound 11 (300 MHz, $CDCl_3$) :

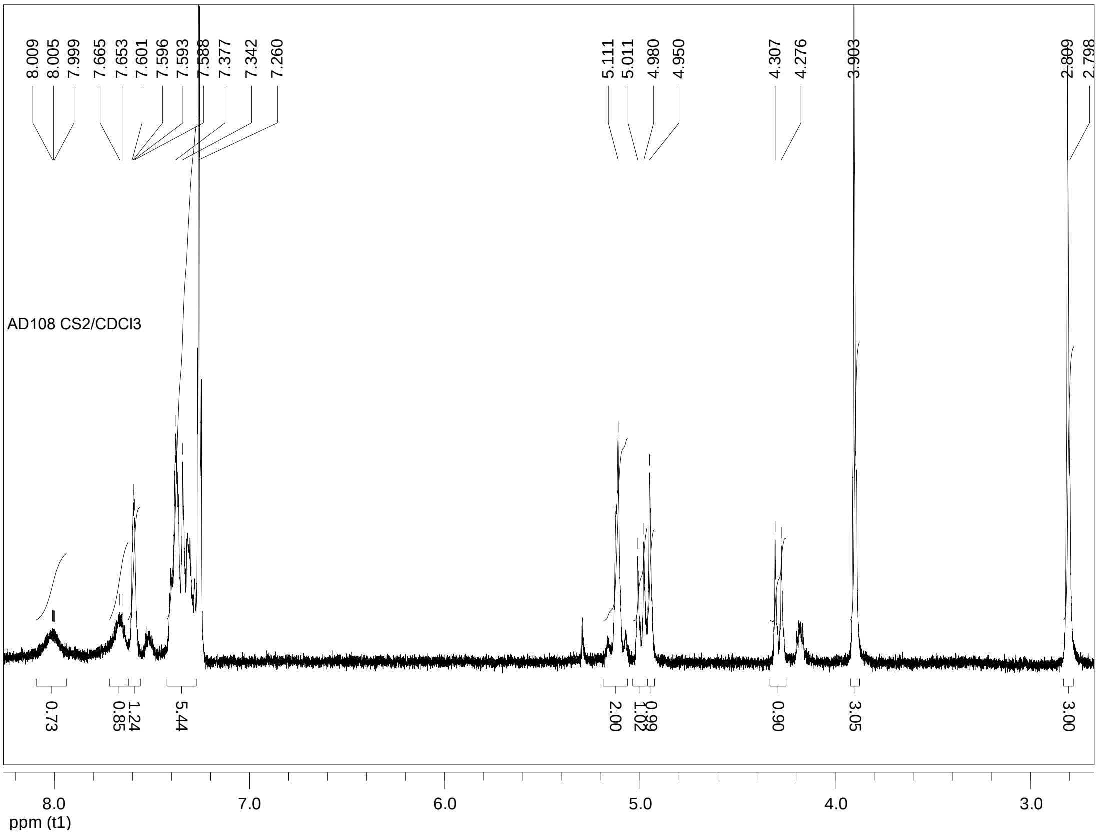

Figure S20: ^{1}H NMR of dyad 12 (500 MHz, 298K, $CDCl_3$) :

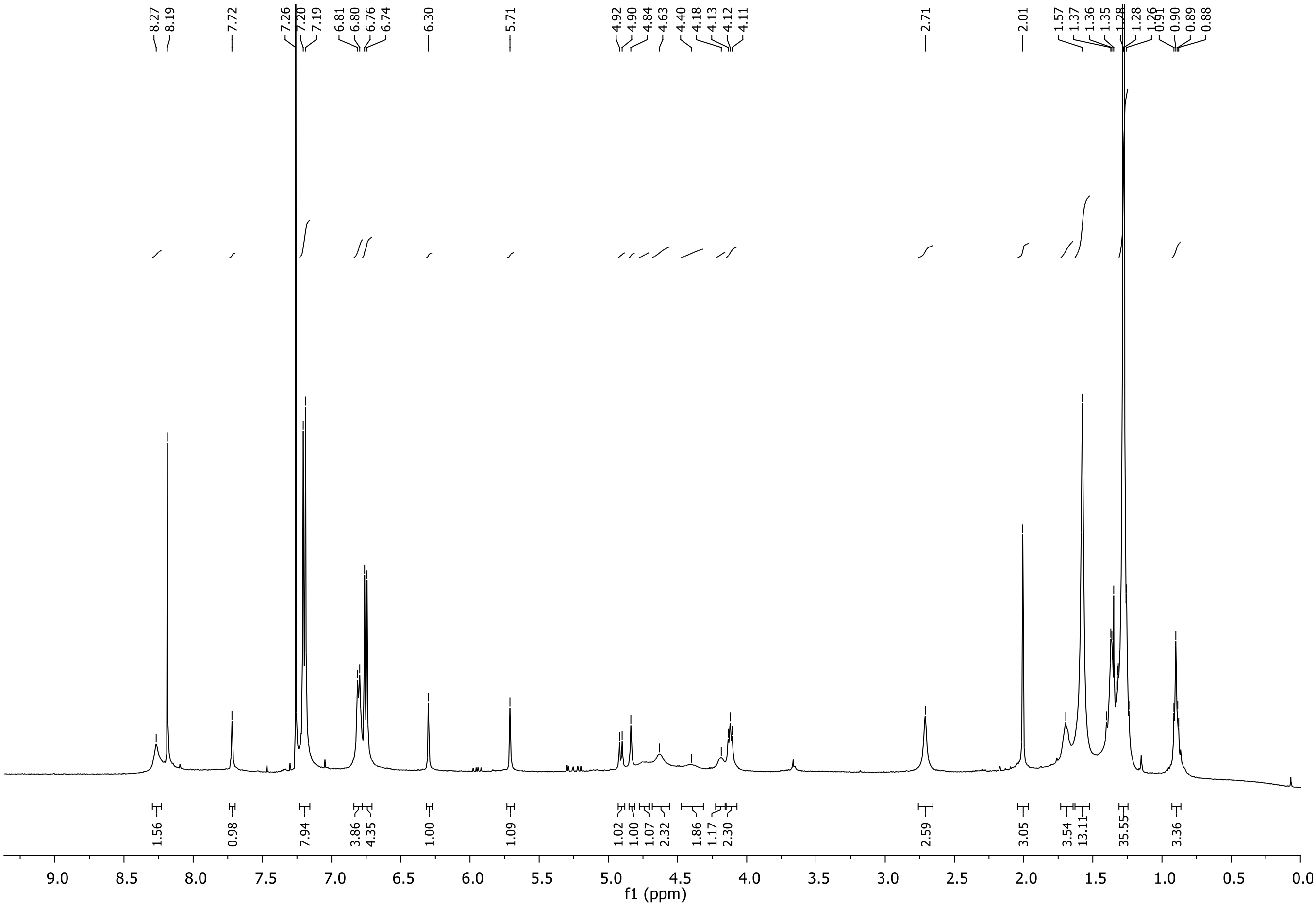

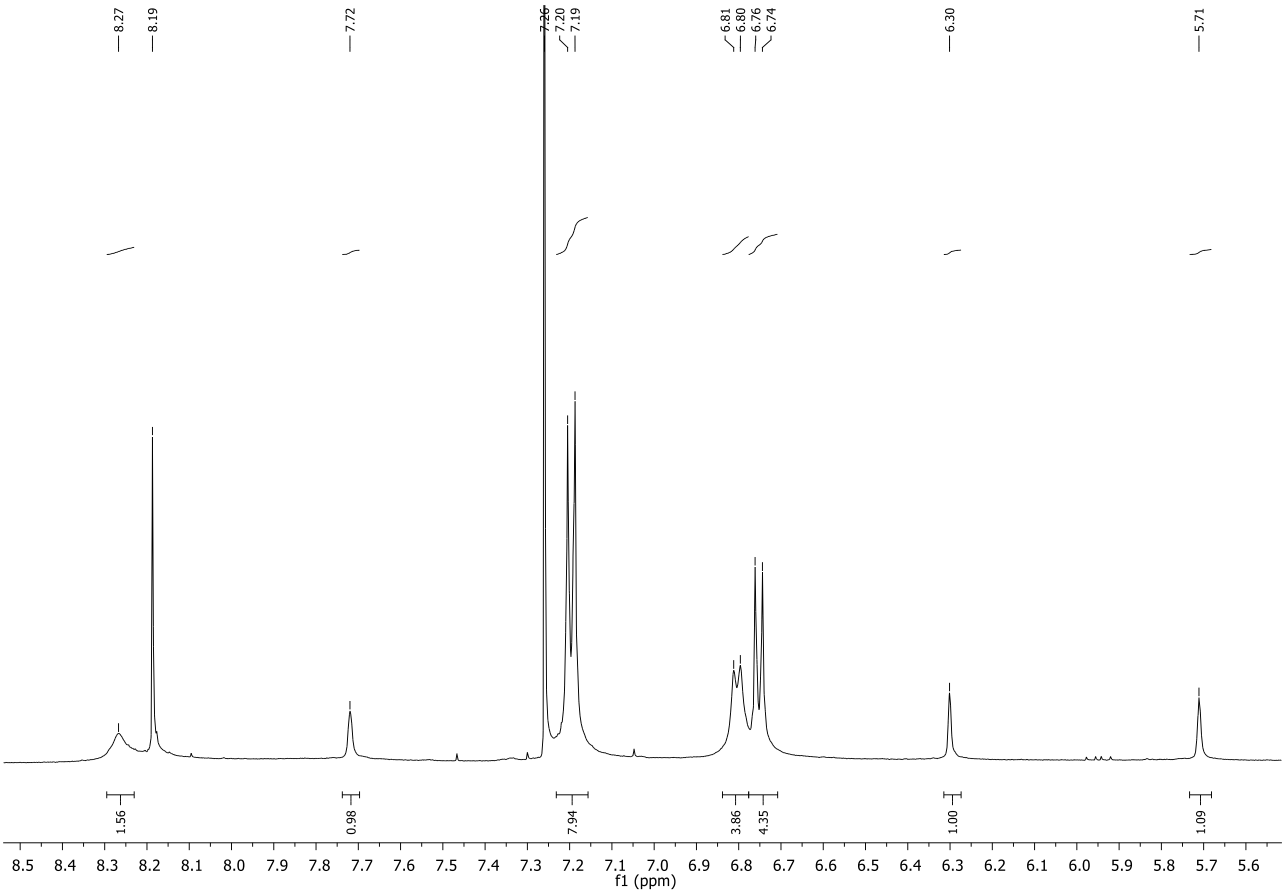

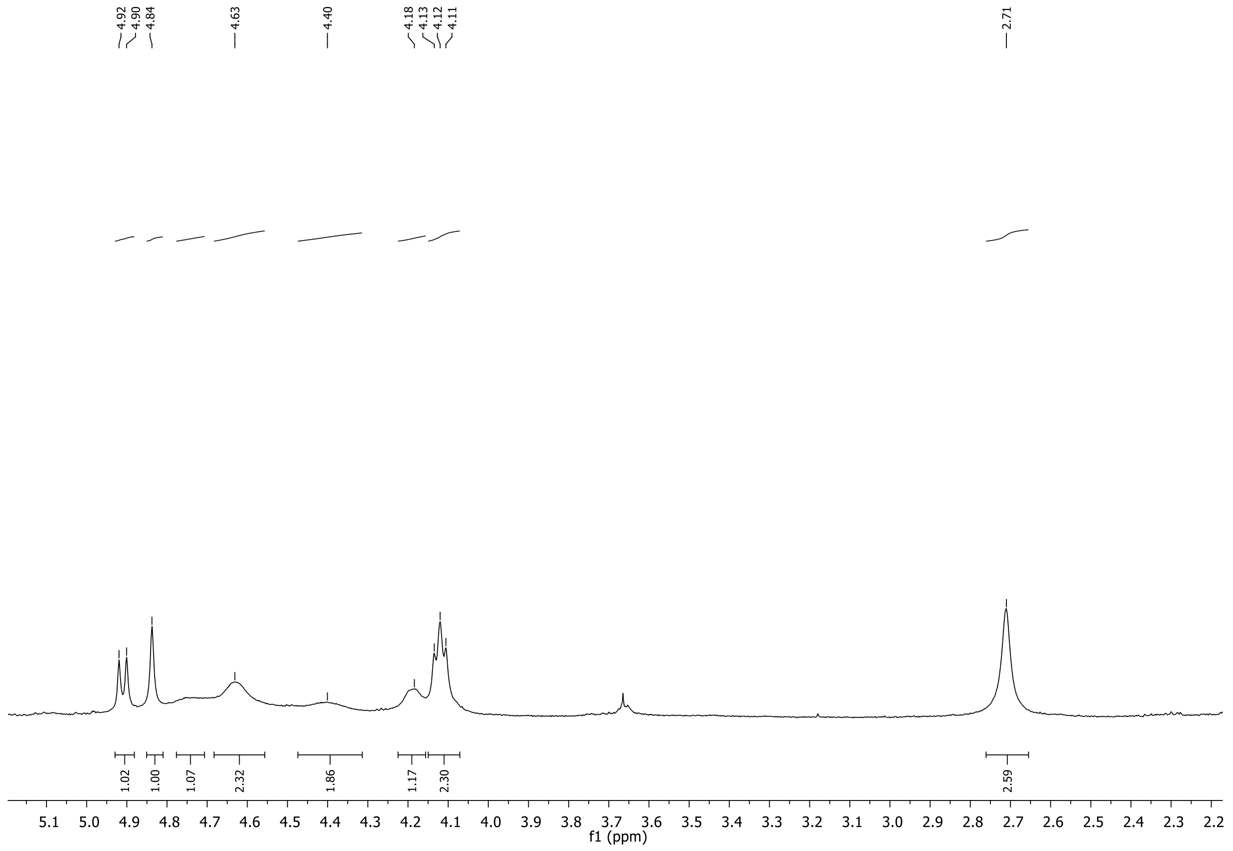

Figure S21: ^{13}C NMR of dyad 12 (500 MHz, 298 K, $CDCl_3$) :

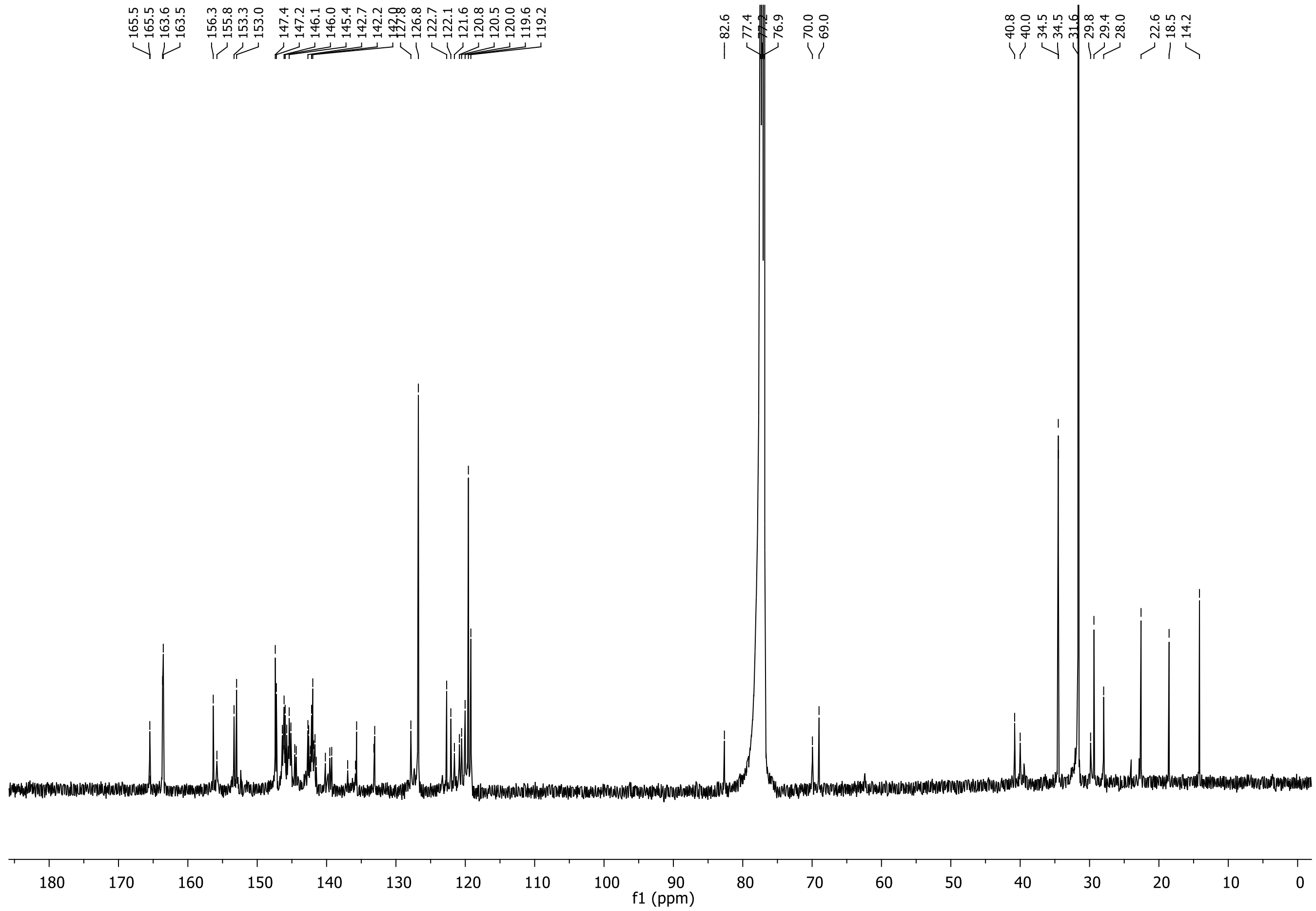

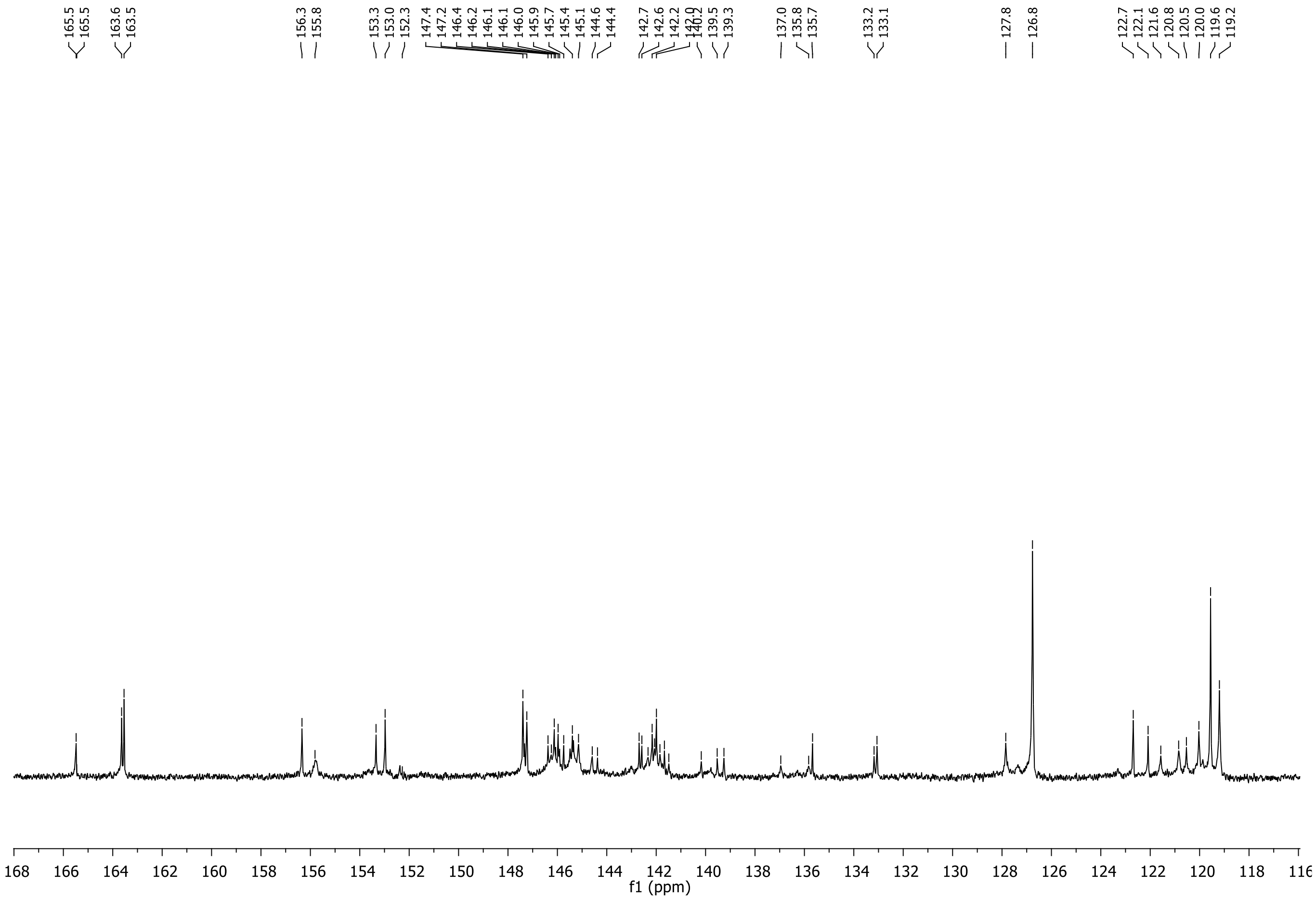
165.5
165.5
163.6
163.5
156.3
155.8
153.3
153.0
152.3
147.4
147.2
146.4
146.2
146.1
146.1
146.0
145.9
145.7
145.4
145.1
144.6
144.4
142.7
142.6
142.2
142.0
140.2
139.5
139.3
137.0
135.8
135.7
133.2
133.1
127.8
126.8
122.7
122.1
121.6
120.8
120.5
120.0
119.6
119.2
168 166 164 162 160 158 156 154 152 150 148 146 144 142 140 138 136 134 132 130 128 126 124 122 120 118 116
f1 (ppm)

Figure S22: Experimental HRMS Spectrum of dyad 12 (MALDI, pos. mode, DCTB):

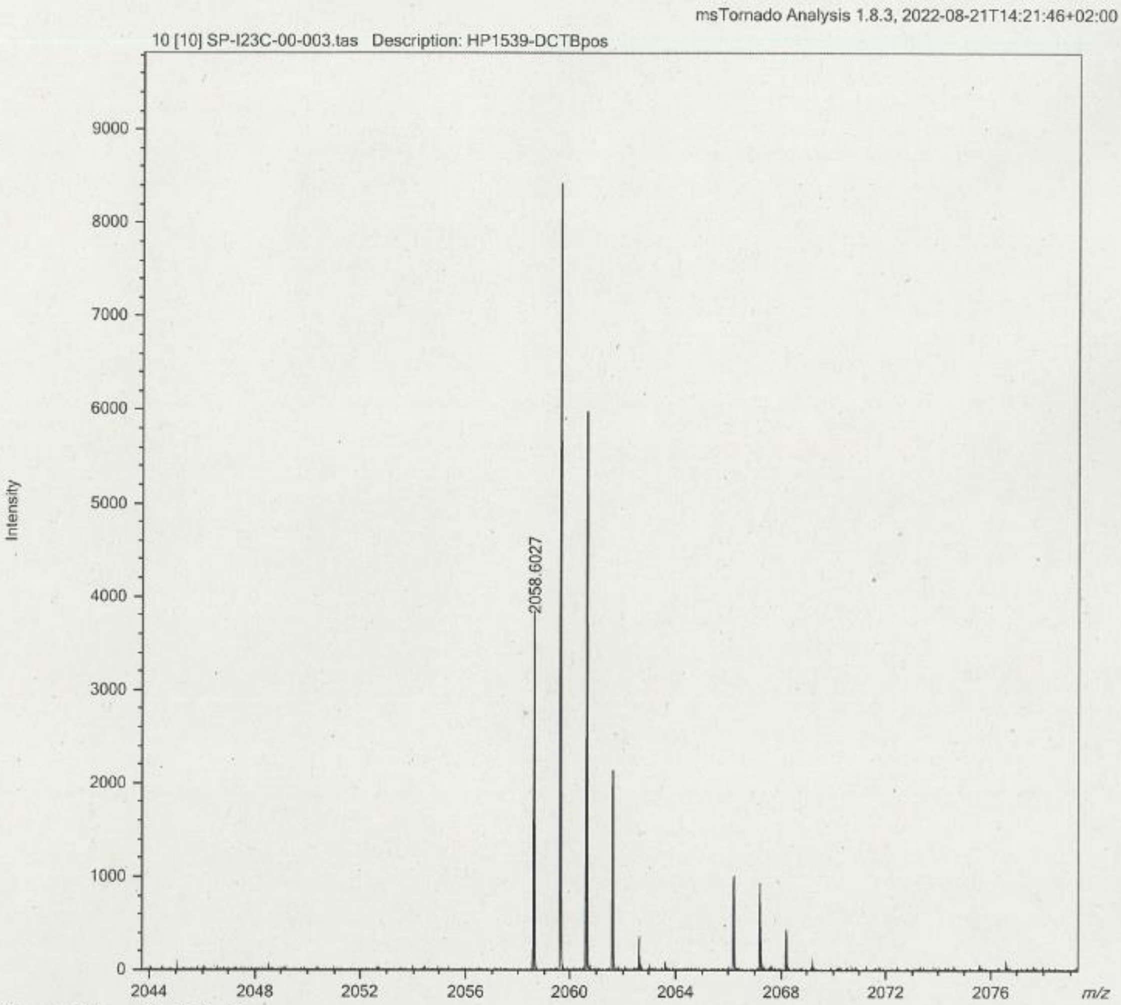

Elemental Composition Estimation

Parameters:

Mass	Tolerance	Electron Mode	Charge	DBE Range	Max Results
2058.60268 ± 0.00309	1.5 ppm	Even	+1	-0.5 - 200.0	100

Elements

C		H		N		O		S	
C	0 - 150	H	0 - 100	N	0 - 5	O	0 - 13	S	0 - 0

Results:

#	Formula	Mass	DBE	Abs. Error (u)	Error (u)	Error (ppm)
1	C145 H84 N3 O12	2058.60495	105.5	0.00227	-0.00227	-1.10

Theoretical spectrum for molecular peak $[M-H]^+$ for dyad 12:

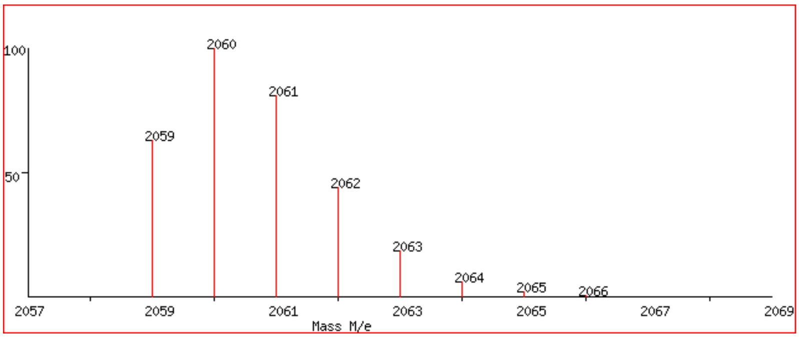

Electrochemical characterizations

Cyclic voltammetry were carried out in a three-electrode cell equipped with a platinum millielectrode as working electrode, a platinum wire counter electrode and a silver wire in a 0.01 M solution of $AgNO_3$ in CH_3CN as a reference electrode, in the presence of 0.10 M tetrabutylammonium hexafluorophosphate (TBAHP – puriss quality) in CH_2Cl_2 (or a mixture with 20% CS_2 in the case of compound **11**) as supporting electrolyte. The ferrocene/ferrocenium couple (Fc^+/Fc) was used as internal reference and the potentials were expressed versus Fc^+/Fc. All experiments were performed in a glove box containing dry, oxygen-free (<1 ppm) argon, at room temperature. Electrochemical experiments were carried out with an EGG PAR 273A potentiostat.

The electrochemical properties of **dyad 9** have been analyzed by cyclic voltammetry in CH_2Cl_2 (or a mixture with CS_2 in the case of compound **11**), Compounds **8** and **11** were used as model derivatives allowing the determination of the reduced species. The voltammograms have been deconvoluted in order to obtain the number of electrons involved in the processes.

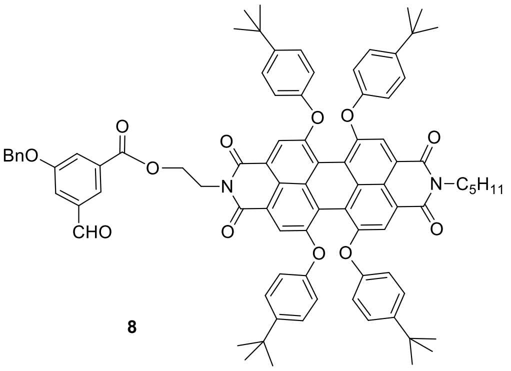

8

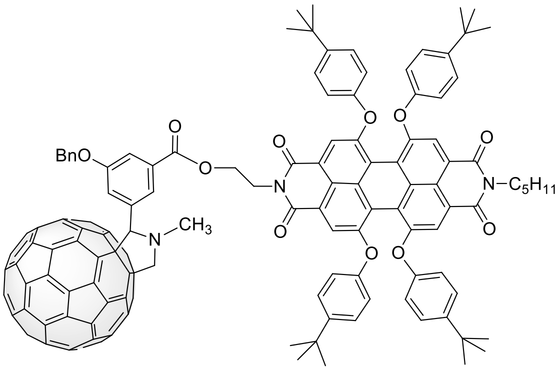

C60- PDI dyad 9

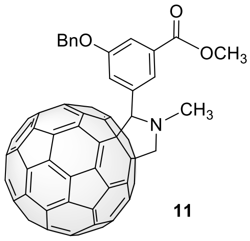

11

Figure S23: Cyclic voltammograms (left) and deconvoluted cyclic voltammograms (right) (C = 5.10^{-4}M, Pt electrodes, CH_2Cl_2 (20%CS_2 for 11) / *n*-Bu_4NPF_6 (0.1M) :

Compound 8 :

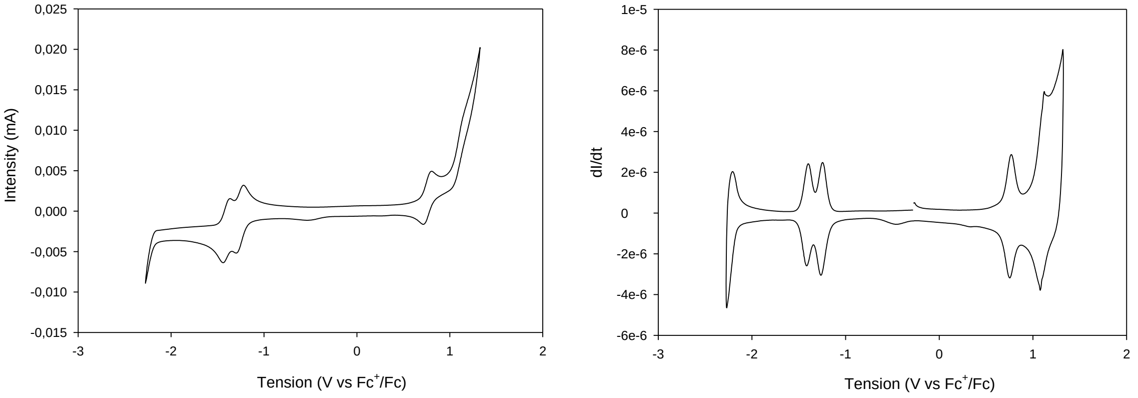

Compound 11 :

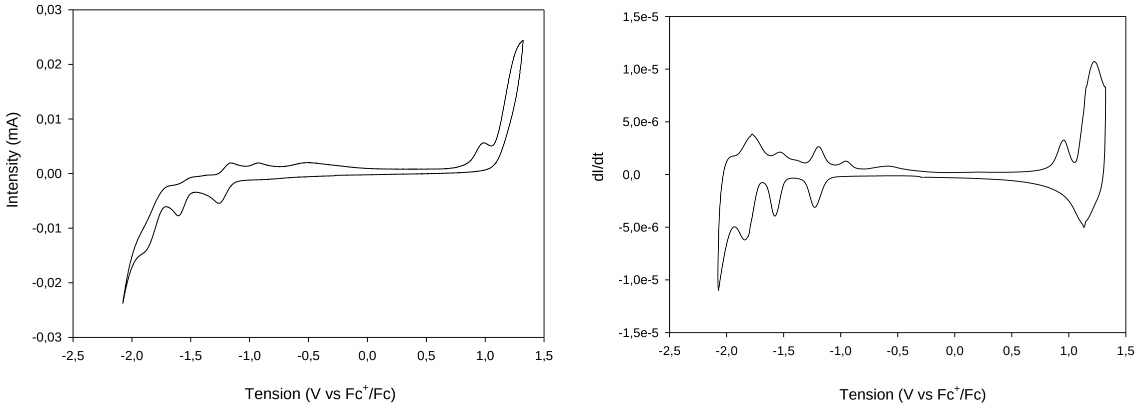

Dyad 9:

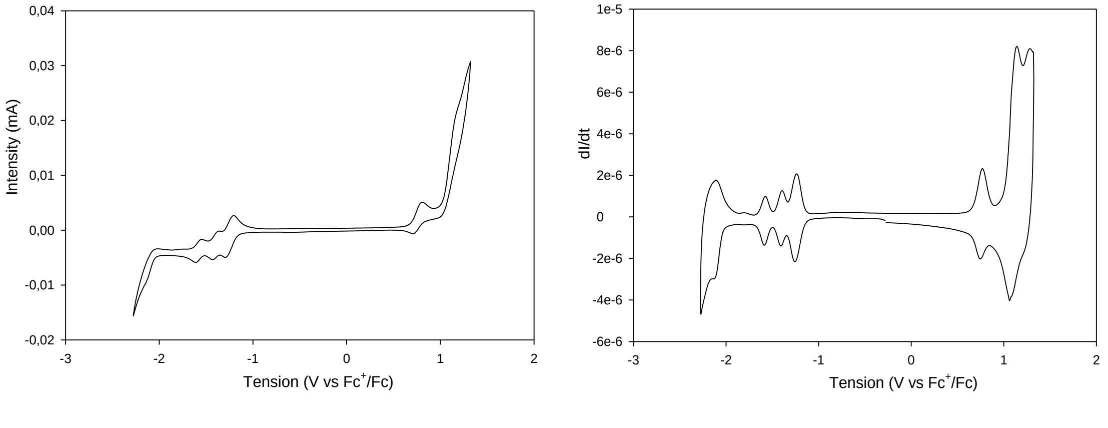

Photophysical characterizations

1) UV-Vis absorption properties

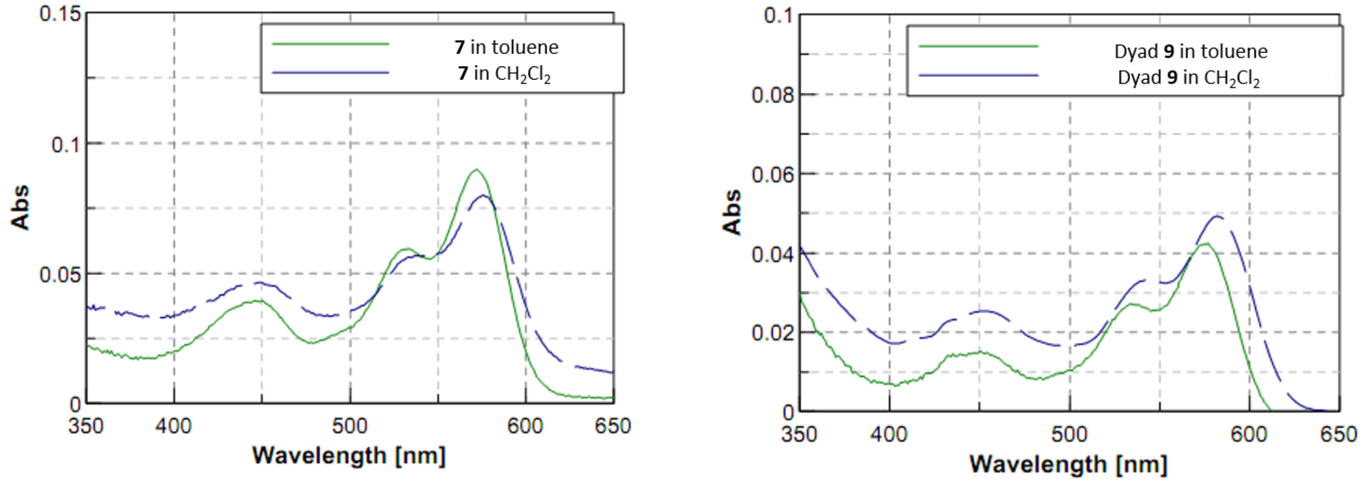

Figure S24: Absorption spectra in toluene and CH_2Cl_2

2) Fluorescence properties

Fluorescence emission spectra of **7** and **Dyad 9** were recorded in toluene and CH_2Cl_2.

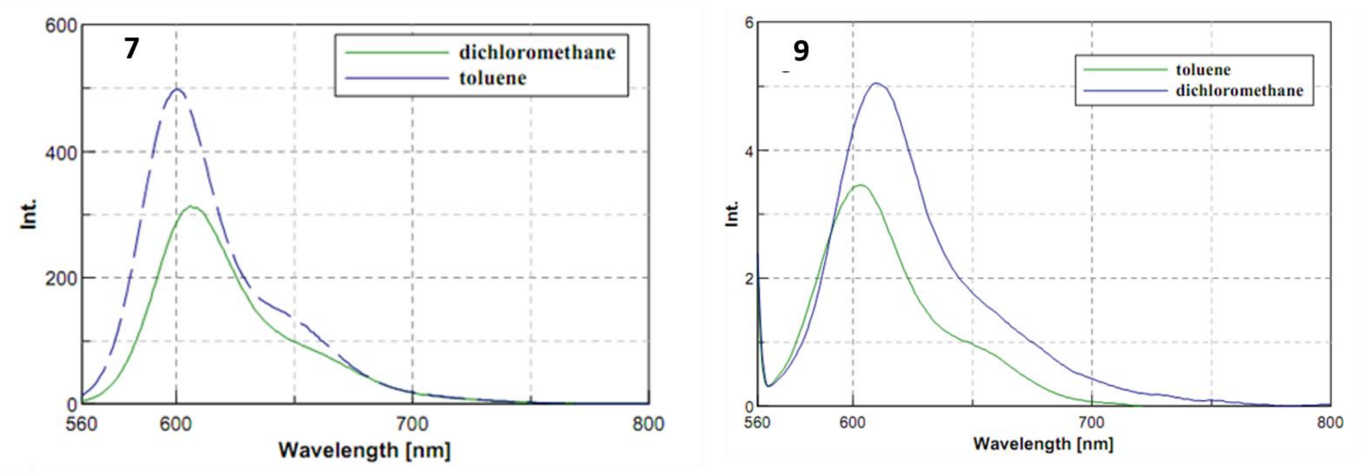

Figure S25: Emission spectra of compound 7 (left) for λ_{ex} = 520 nm and Dyad 9 (right) for λ_{ex} = 550 nm

A strong quenching was observed in the case of **Dyad 9**, this is due to the strong interaction between the PDI and C_{60} in the excited state. The quantum yields were calculated using cresyl violet perchlorate as reference (ϕ_F=0.54 in ethanol)[115] and the following equation:

$$\Phi_F = \Phi_{ref} \times \frac{I}{I_{ref}} \times \frac{OD_{ref}}{OD} \times \frac{n^2}{n_{ref}^2},$$ where

Φ_{ref} – quantum yield of the reference;

I - the integrated intensity of fluorescence spectra for the studied compound;

I_{ref} - the integrated intensity of fluorescence spectra for the reference compound;

OD – optical density of the studied compound at $\lambda_{excitation}$;

OD_{ref} – optical density of the reference compound at $\lambda_{excitation}$;

n – refractive index of solvent for the studied compound;

n_{ref} – refractive index of solvent for the reference compound.

For **Dyad 9** a fluorescence emission (in CH_2Cl_2) at 710 nm characteristic for fullerene C_{60} can be observed in the normalized spectrum above. This can be interpreted as a proof of the energy transfer that takes place between the two moieties.

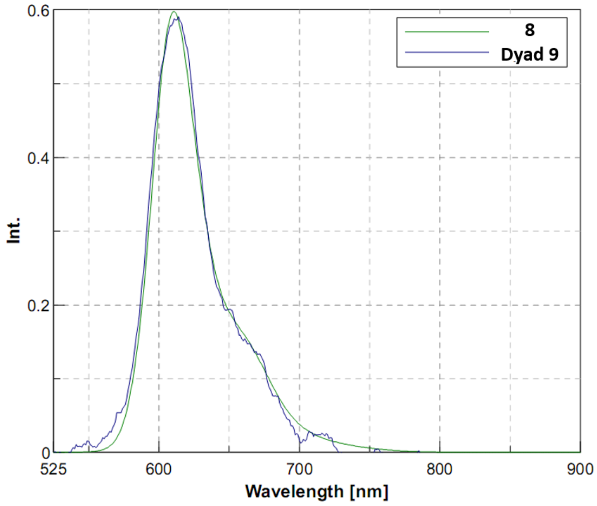

Figure S26: Normalized fluorescence intensity (ex. 520 nm) c = 5x10^{-6} M for compound 8 and dyad 9.

The obtained quantum yield for **Dyad 1** presents a strong quenching, around 99.7%. The high quenching is present both in dichloromethane and toluene; although a lower value of around 99% was obtained for toluene.

[115] H. Du, R.-C. A. Fuh, J. Li, L. A. Corkan and J. S. Lindsey, *Photochemistry and Photobiology*, Wiley Publishing: New York, United States,1998**,** *68*, 141-142.

Polymer derivatives (POL1, POL2, POL 3)

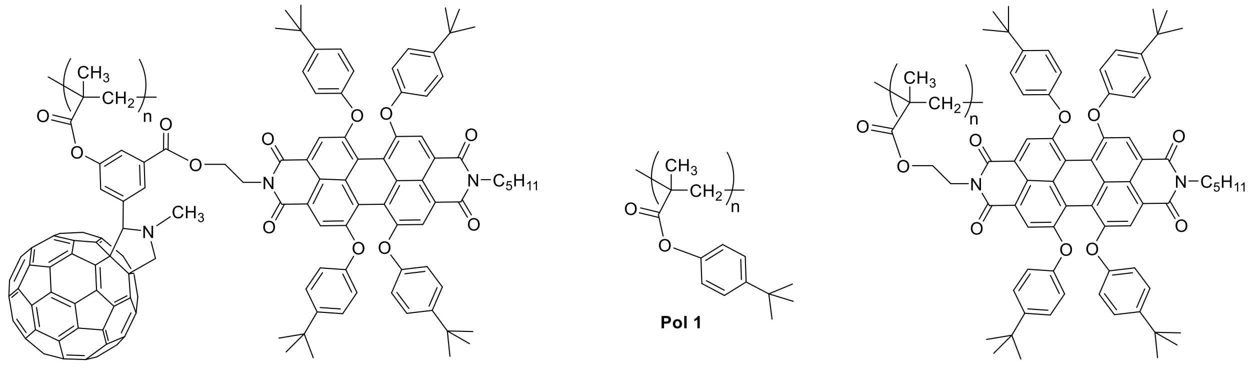

DSC and TGA analysis

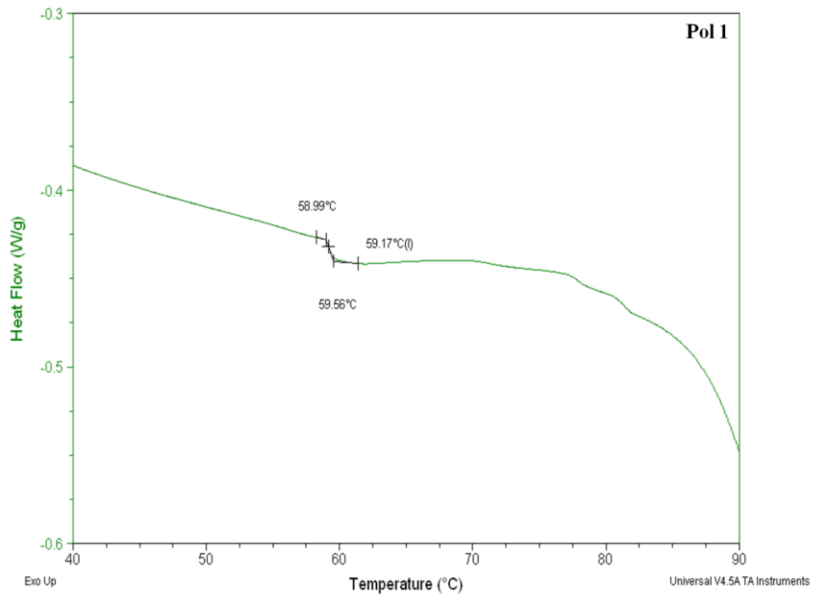

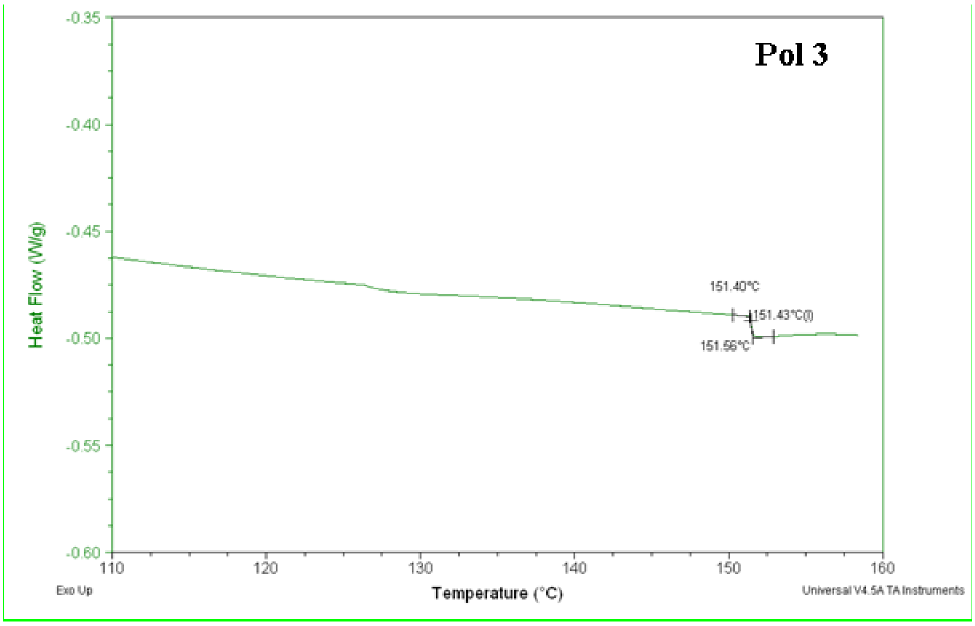

Figure S27: DSC analysis, determination of the T_g for Pol 1 and Pol 3

TGA (thermogravimetric analysis) has also been conducted on the three polymeric derivatives at a heating rate of 10°C min^{-1} under nitrogen atmosphere using a Q500 TA Instruments.

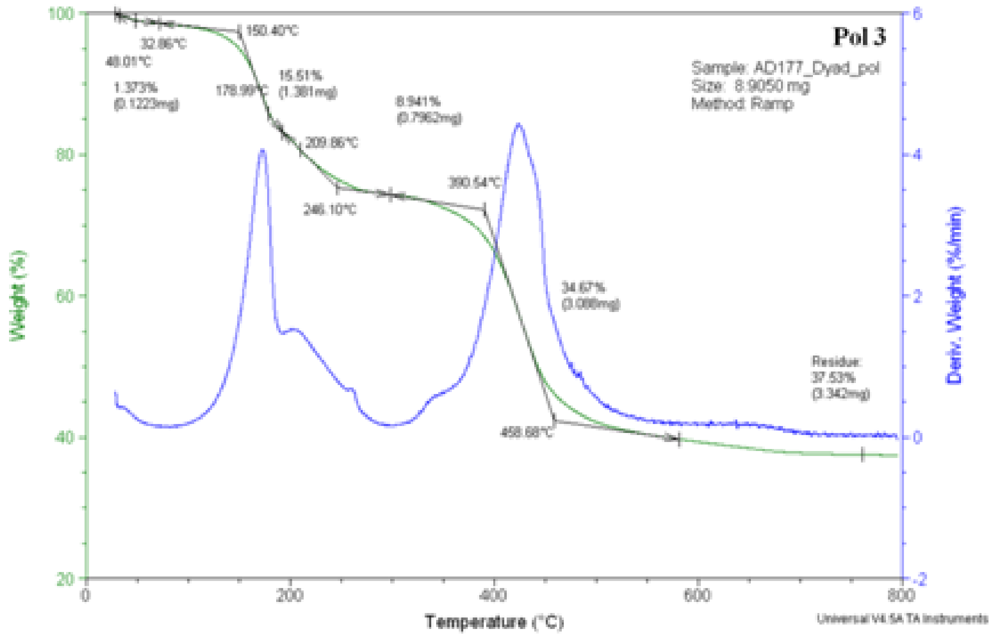

Figure S28: TGA and DTA analysis for Pol 3

UV-VIS spectra of polymers

The concentration of dye/dyad in the polymer samples was determined using a colorimetric technique employing Lambert Beers' law ($A = \varepsilon \cdot c \cdot l$). A calibration curve was obtained by plotting the absorbance against concentration of dye solutions of known concentrations.

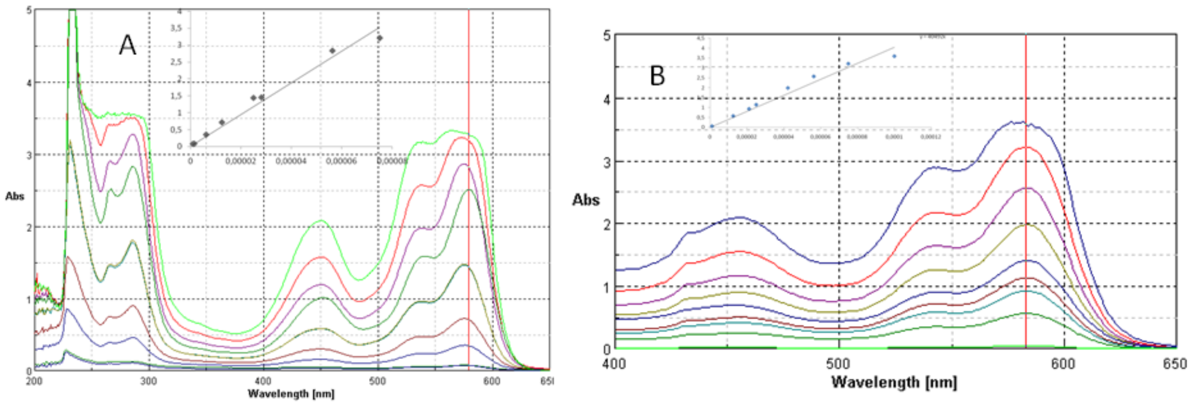

Figure S29: UV-VIS spectra and calibration curve for (A) POL 2 and (B) POL 3

Figure S30: ^{1}H NMR spectrum (300 MHz, $CDCl_3$) of C_{60}-PDI polymer Pol 3 :

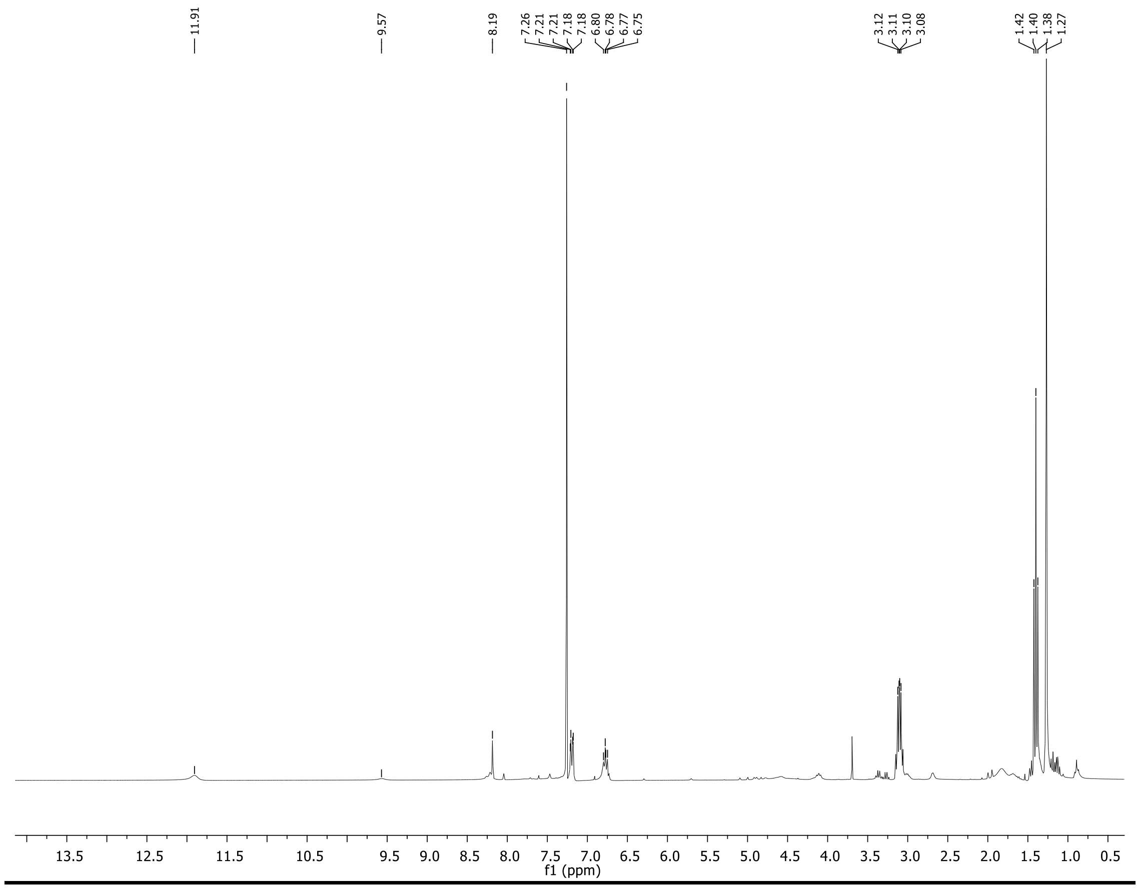

Figure S31: ^{13}C NMR spectrum (75 MHz, $CDCl_3$) of C_{60}-PDI polymer Pol 3 :

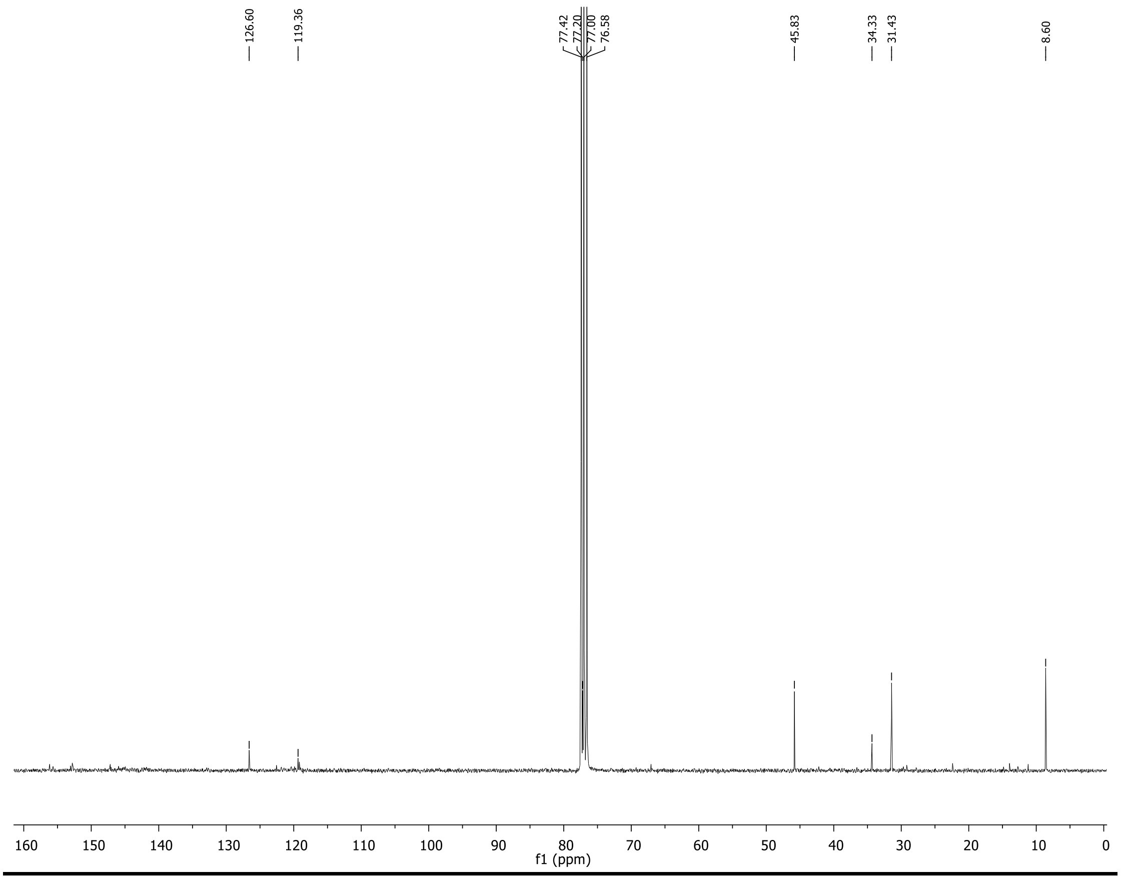

Figure S32: ^{1}H NMR spectrum (300 MHz, CDCl3) of PDI polymer Pol 2 :

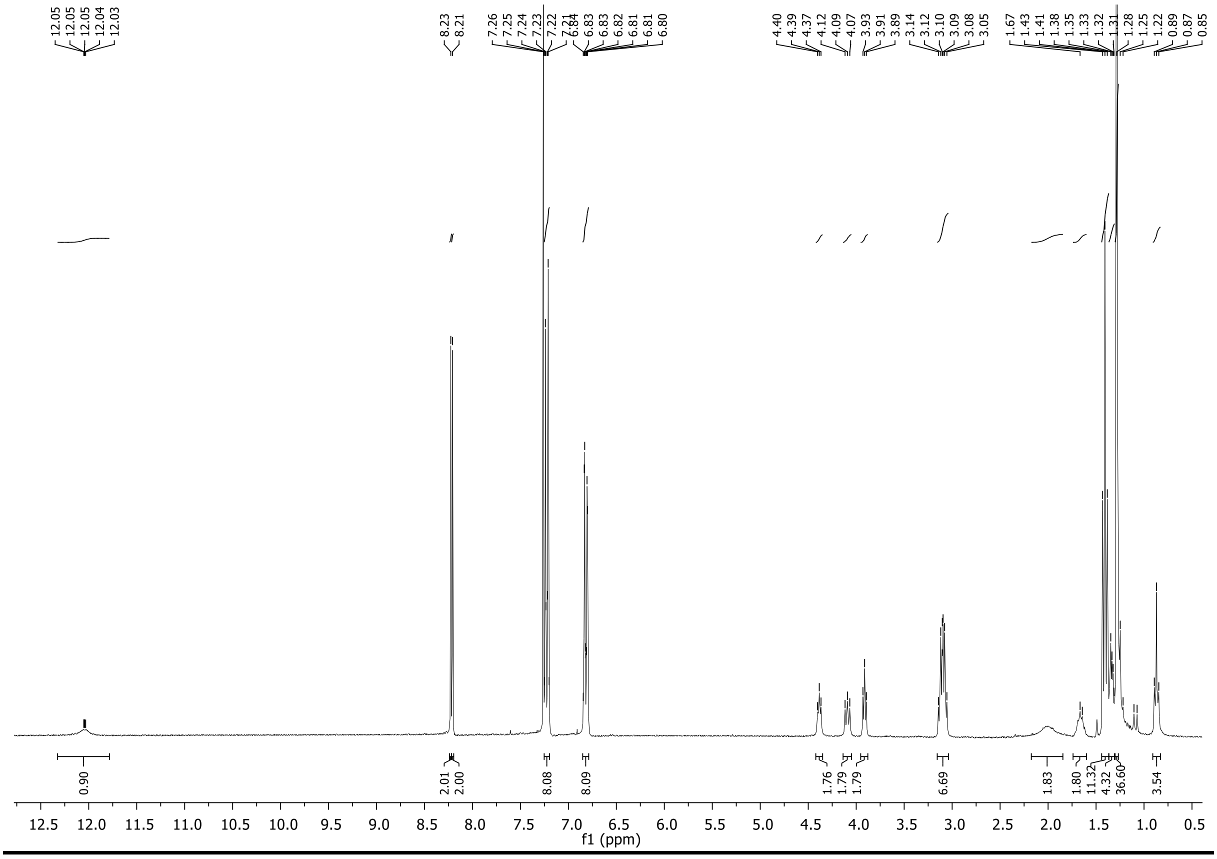

Figure S33: ^{13}C NMR spectrum (75 MHz, CDCl3) of PDI polymer Pol 2 :

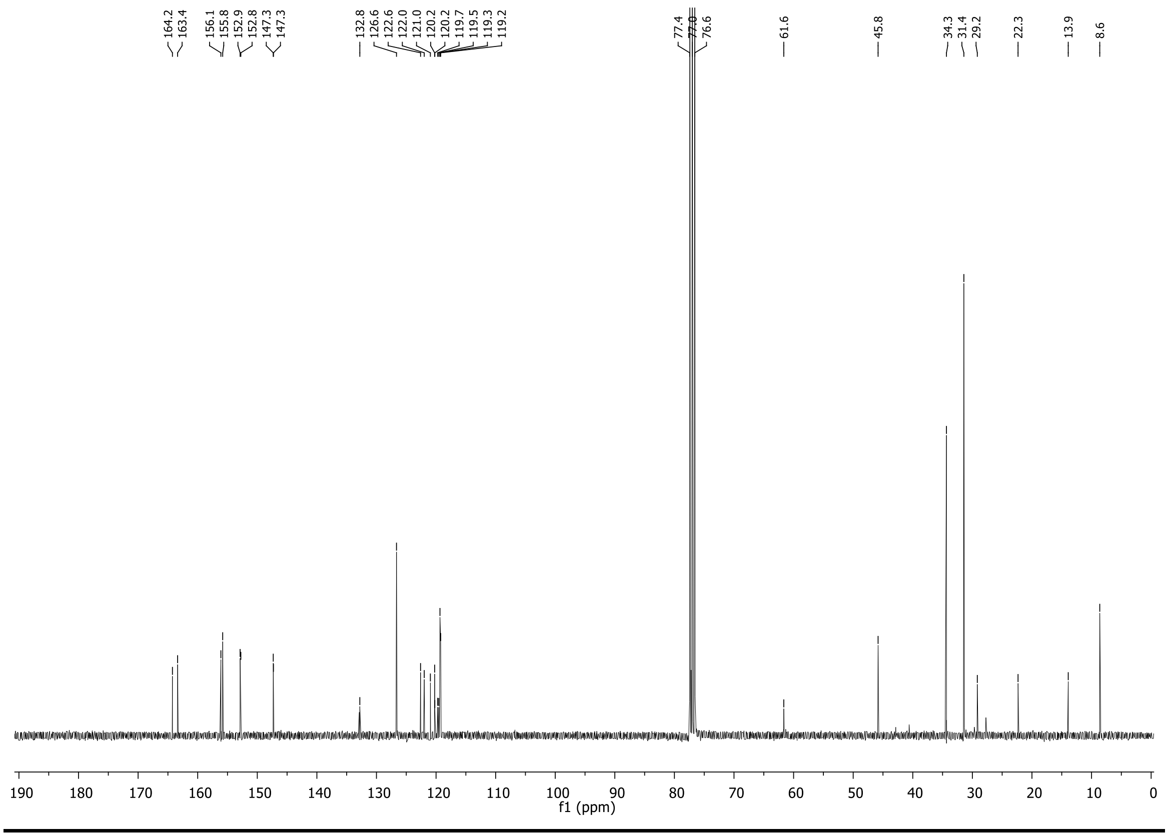

Supplement: Supplementary file 1 [file molecules-27-06522-s001.zip › molecules-1915171-supplementary.pdf]
